# Metabolic profiling of smoking, associations with type 2 diabetes and interaction with genetic susceptibility

**DOI:** 10.1007/s10654-024-01117-5

**Published:** 2024-03-31

**Authors:** Yuxia Wei, Sara Hägg, Jonathan K. L. Mak, Tiinamaija Tuomi, Yiqiang Zhan, Sofia Carlsson

**Affiliations:** 1 Institute of Environmental Medicine, Karolinska Institutet, Nobels väg 13, Stockholm, 17177 Sweden; 2 Department of Medical Epidemiology and Biostatistics, Karolinska Institutet, Stockholm, Sweden; 3 Department of Clinical Sciences in Malmö, Clinical Research Centre, Lund University, Malmö, Sweden; 4 Institute for Molecular Medicine Finland, Helsinki University, Helsinki, Finland; 5 Department of Endocrinology, Abdominal Center, Research Program for Diabetes and Obesity, Folkhälsan Research Center, Helsinki University Hospital, University of Helsinki, Helsinki, Finland; 6 School of Public Health (Shenzhen), Sun Yat-Sen University, Shenzhen, China; 7 Department of Pharmacology and Pharmacy, Li Ka Shing Faculty of Medicine, The University of Hong Kong, Hong Kong SAR, China

**Keywords:** Smoking, Metabolomics, Type 2 diabetes, Genetic risk score

## Abstract

**Background:**

Smokers are at increased risk of type 2 diabetes (T2D), but the underlying mechanisms are unclear. We investigated if the smoking-T2D association is mediated by alterations in the metabolome and assessed potential interaction with genetic susceptibility to diabetes or insulin resistance.

**Methods:**

In UK Biobank (*n* = 93,722), cross-sectional analyses identified 208 metabolites associated with smoking, of which 131 were confirmed in Mendelian Randomization analyses, including glycoprotein acetyls, fatty acids, and lipids. Elastic net regression was applied to create a smoking-related metabolic signature. We estimated hazard ratios (HR) of incident T2D in relation to baseline smoking/metabolic signature and calculated the proportion of the smoking-T2D association mediated by the signature. Additive interaction between the signature and genetic risk scores for T2D (GRS-T2D) and insulin resistance (GRS-IR) on incidence of T2D was assessed as relative excess risk due to interaction (RERI).

**Findings:**

The HR of T2D was 1·73 (95% confidence interval (CI) 1·54 − 1·94) for current versus never smoking, and 38·3% of the excess risk was mediated by the metabolic signature. The metabolic signature and its mediation role were replicated in TwinGene. The metabolic signature was associated with T2D (HR: 1·61, CI 1·46 − 1·77 for values above vs. below median), with evidence of interaction with GRS-T2D (RERI: 0·81, CI: 0·23 − 1·38) and GRS-IR (RERI 0·47, CI: 0·02 − 0·92).

**Interpretation:**

The increased risk of T2D in smokers may be mediated through effects on the metabolome, and the influence of such metabolic alterations on diabetes risk may be amplified in individuals with genetic susceptibility to T2D or insulin resistance.

**Supplementary Information:**

The online version contains supplementary material available at 10.1007/s10654-024-01117-5.

## Introduction

Cigarette smokers are at increased risk of developing type 2 diabetes: Observational studies show a 20-60% elevated risk in smokers [1, 2], and Mendelian randomization (MR) studies support a causal relationship [3]. Smokers differ from non-smokers in levels of several metabolites such as lipids and amino acids, according to recent studies using high-throughput metabolite data (metabolomics) [4–14]. Whether this reflects causal effects of smoking is not entirely clear, but a MR study indicated that smoking influences levels of glycoprotein acetyls (an inflammatory biomarker), free fatty acids, and lipids in high-density lipoproteins (HDL) [15]. Previous observational studies on metabolomic effects of smoking were hampered by small sample sizes (< 1500). To elucidate the full range of metabolites affected by smoking, there is a need for large-scale studies and additional MR studies which are less susceptible to reverse causation and confounding bias than observational studies for causal inference.

Adverse effects of smoking, and specifically nicotine, on insulin sensitivity have been documented in experimental studies [16]. Abnormal levels of some of the smoking associated metabolites are detrimental to insulin sensitivity according to animal and cell culture studies [17, 18]. In addition, several of the metabolites have been linked to type 2 diabetes [19]. Therefore, the mechanism linking smoking to type 2 diabetes and insulin resistance may involve modifications of the metabolome. People with genetic susceptibility to diabetes or insulin resistance may be more vulnerable to these alterations since smoking has been shown to exacerbate the genetic susceptibility to diabetes-related traits [20]. Whether this is the case remains to be investigated.

Leveraging metabolomics data in approximately 100,000 UK Biobank (UKB) participants, our aim was to identify metabolites affected by smoking, create a metabolic signature of smoking, and investigate whether this signature mediates the association between smoking and incidence of type 2 diabetes. We externally validated this signature in the TwinGene study. We also assessed potential interactions between the metabolic signature and genetic susceptibility to insulin resistance or type 2 diabetes on incidence of type 2 diabetes. The overarching goal was to provide new insights on how smoking affects the development of diabetes by modifying circulating metabolites.

## Methods

### Study population

**Fig. 1:**
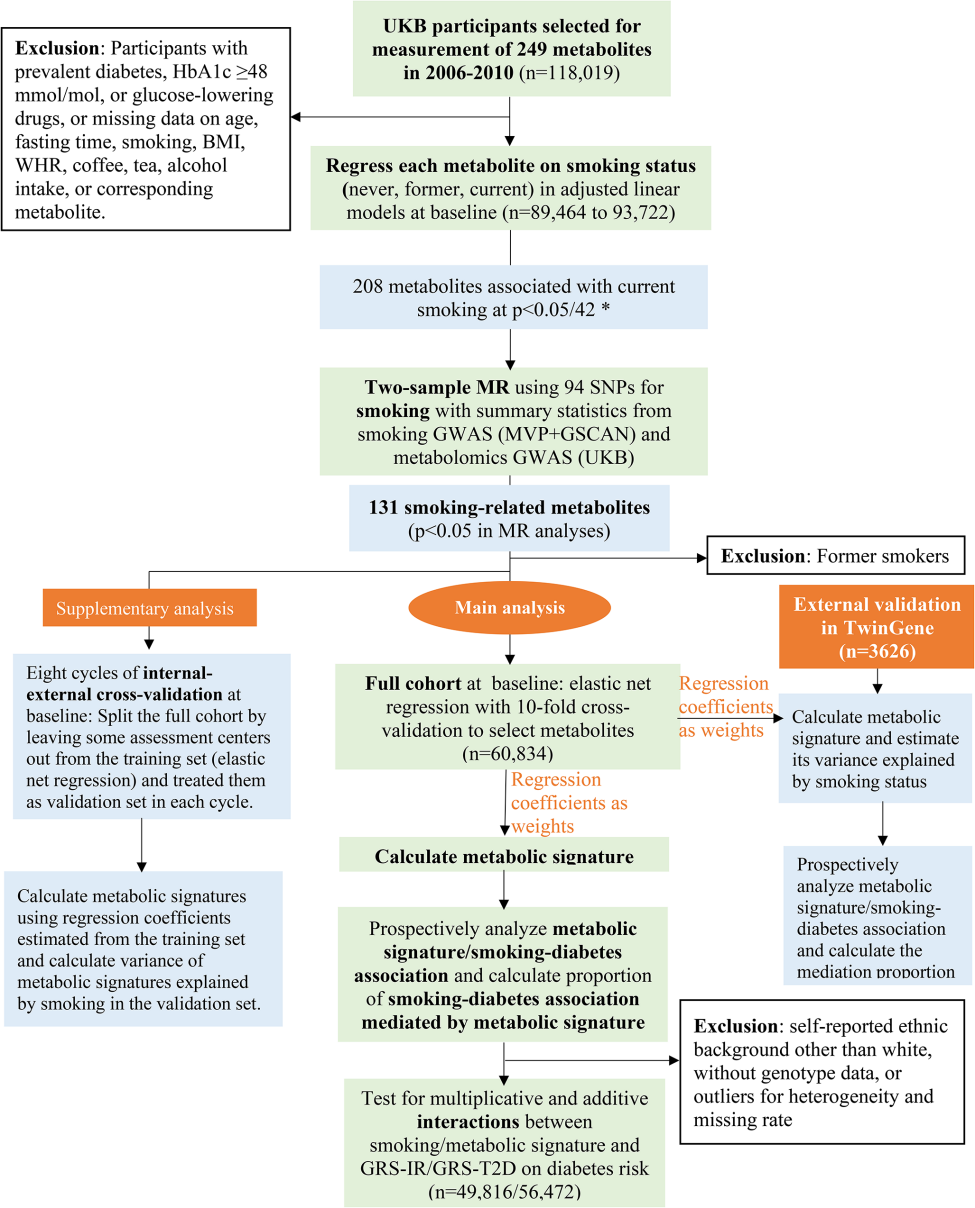
Flow chart of the study design. UKB: the UK Biobank study; BMI: body mass index; WHR: waist-to-hip ratio; MR: Mendelian randomization; SNP: single nucleotide polymorphism; GWAS: genome-wide association study; MVP: the Million Veteran Program; GSCAN: the GWAS & Sequencing Consortium of Alcohol and Nicotine use study; GRS-IR: genetic risk score for insulin resistance; GRS-T2D: genetic risk score for type 2 diabetes. Participants were followed for incidence of type 2 diabetes from baseline 2006–2010 until 2022. * 42 principal components explained > 99% of the variance in the 249 metabolites

The UKB study enrolled half a million participants aged 37–73 years from 22 assessment centers in the UK in 2006–2010 [21]. Eligible for the present study were the 118,019 participants who had metabolomics data collected at baseline. We excluded participants with diagnosed or undiagnosed (HbA1c ≥ 6·5% [48 mmol/mol] or taking glucose-lowering drugs) diabetes, taking lipid-lowering drugs, or with missing data on age, fasting time, smoking status, body mass index (BMI), waist-to-hip ratio (WHR), alcohol intake, coffee consumption, tea consumption, or corresponding metabolite for the analysis of smoking-metabolite associations (Fig. 1). The subsequent sample sizes ranged from 89,464 to 93,722 in the analyses of the available metabolites or derived measures (ratios or percentages, also called “metabolite” in the following sections for convenience) at baseline. Among them, 5,138 participants had metabolomics data collected again at a repeat assessment in 2012–2013 and the sample sizes for each metabolite ranged from 3,632 to 3,797. The UKB study was approved by the North West Multi-center Research Ethics Committee [22]. Our study was performed under the UK Biobank Project 84,778, with ethical approval from the Swedish ethical review board (2022-02293-01). All participants provided informed consent to participate.

### Smoking and other lifestyle factors at baseline and repeat assessment

Participants were asked about current and past smoking status, and classified as never, former, or current smokers. BMI was calculated from measured standing height and body weight, while WHR was calculated by dividing waist circumference by hip circumference. Consumption of different food items such as alcohol intake during the past 12 months was obtained from a short, touchscreen food frequency questionnaire (FFQ). Information on physical activity was also obtained through questionnaires.

### Metabolomic profiling and genetic data

The metabolomic data was analysed in 2019–2020, from non-fasting EDTA plasma samples collected at baseline or the repeat assessment, using a high-throughput nuclear magnetic resonance(NMR)-based platform developed by Nightingale Health Ltd [23]. The NMR platform measured 249 metabolites, including glycoprotein acetyls, lipids in 14 subclasses, fatty acids, amino acids, ketone bodies, and glycolysis metabolites [23]. We constructed genetic risk scores for insulin resistance (GRS-IR) and type 2 diabetes (GRS-T2D) based on 5 [24] and 38 [25] independent SNPs genotyped in UKB participants, respectively. Such SNPs have been used by previous studies to create genetic risk scores [24, 25], calculated as the weighted (by effect sizes) sum of the number of risk alleles (0, 1, 2) [25] (eTable 1). Participants were then categorized into low (quintile 1), intermediate (quintile 2–4), and high (quintile 5) genetic risk groups [25, 26].

### Type 2 diabetes

Incident cases of diabetes was identified during follow-up until 2022. Type 2 diabetes was defined as a corresponding diagnostic code (UKB data field 130,709) identified from hospital admissions, primary care, or death register, or a diabetes diagnosis collected through verbal interview at repeat assessments. The date of diagnosis is defined as the earliest date of diabetes diagnosis recorded through either self-report at repeat assessment, inpatient hospital data, primary care, or death register.

### Statistical analysis

### Smoking-related metabolites identified by cross-sectional and MR analyses

Metabolites deviating from normal distribution were log-transformed, and all metabolites were rescaled (divided by standard deviation [SD]). Each of the 249 metabolites measured at baseline was treated as the response variable with smoking status (current, former, or never) as the exposure in a linear model, with adjustment for age, sex, assessment center, education, ethnicity, Townsend deprivation index, fasting hours, BMI, WHR, physical activity, and consumption of different food items such as alcohol and coffee. From these models, we also obtained the variance (R^2^) explained by smoking status and covariates for each metabolite. We repeated the linear regression for each of the 249 metabolites using repeat assessment data.

The 249 metabolites are correlated with each other. We performed a principal component analysis and 42 principal components explained > 99% of variance in these metabolites. We therefore corrected for multiple tests by setting *p* < 0·05/42 (accounting for the 42 independent components which explained the most variance in the metabolites) as the statistical significance threshold to identify metabolites associated with current smoking at baseline [27, 28]. We then performed two-sample MR analyses for the metabolites identified at baseline (Fig. 1). The MR analyses were based on 94 independent SNPs as instrumental variables (IV) for smoking initiation in Europeans [29], and summary statistics for the SNP-metabolite associations from a recent genome-wide association study (GWAS) of metabolomics in UKB [30] (eMethod 1; eTable 2). A metabolite was considered potentially causally affected by smoking (referred as “smoking-related metabolite” below) if its association with smoking was in the same direction in the baseline (*p* < 0·05/42) and MR (*p* < 0·05) analyses.

### Metabolic signature of smoking

We derived a smoking-related metabolic signature based on the identified smoking-related metabolites. First, metabolites deviating from normal distribution were log-transformed. Second, we regressed each metabolite on variables (except smoking) which explained parts of its variance and obtained standardized residuals from those regressions [14, 31]. Third, an elastic net regression model was fitted with the standardized residuals as predictors and smoking status (current versus never) as the response variable at baseline, and the alpha and lambda parameters in the regression were chosen using a 10-fold cross-validation approach based on the minimum mean squared error [32]. The elastic net regression combines the Lasso and Ridge penalties and shows robust prediction performance in the existence of multicollinearity [33]. We calculated the smoking-related metabolic signature at baseline as the weighted sum of the metabolites selected by the elastic net regression using the corresponding regression coefficients as weights [32] (Fig. 1). We also calculated metabolic signature at repeat assessment using regression coefficients obtained from baseline. The variance of the metabolic signature explained by smoking status was estimated by regressing the signature on smoking status and covariates at baseline (Fig. 1) and repeat assessment (for internal replication), respectively.

To assess the validity of the metabolic signature, we performed an external validation in TwinGene (*n* = 3626), a cohort nested in the Swedish Twin Register [34], by calculating the signature with regression coefficients obtained from UKB analysis as the weights (Fig. 1, eMethod 2). We also applied eight cycles of internal-external cross-validation [35, 36] by splitting the full UKB cohort into two parts by assessment centers in each cycle, one part for training the model and the remaining part for external validation (Fig. 1, eMethod 3).

### Prospective analyses of smoking/metabolic signature and incidence of type 2 diabetes

We calculated the duration of follow-up from baseline (2006–2010) to the occurrence of diabetes, death, loss to follow-up, or March 1st, 2022, whichever came first. We fitted Cox proportional hazards regression models to estimate the hazard ratios (HRs) and 95% confidence intervals (CIs) for type 2 diabetes in relation to smoking status (current vs. never), each individual smoking-related metabolite (per SD increase), and the smoking-related metabolic signature (per SD increase, or above versus below the median). The models were fitted with attained age as the time scale and adjusted for age groups, sex, assessment center, education, ethnicity, Townsend deprivation index, BMI, physical activity, consumption of different food items, and family history of diabetes (main model). A Cox marginal structural model [37] was fitted to estimate the natural direct and indirect effect of smoking and the proportion of smoking-diabetes association mediated through the metabolic signature (eFigure 1). We also did the above-mentioned analyses in men and women separately. We repeated such analyses in TwinGene (eMethod 2).

### Interaction between smoking/metabolic signature and genetic susceptibility

We hypothesized that the coexistence of genetic susceptibility and high levels of metabolic signature (or smoking) enhance their overall impact on type 2 diabetes (eFigure 1). We wanted to assess additive interaction since it is more of public health relevance than multiplicative interaction [38]. Additive interaction implies that the absolute risk reduction [38] by intervening on the metabolic signature (or smoking) is larger in those with high genetic susceptibility than that in those with low genetic susceptibility. The existence of both additive and multiplicative interactions is the strongest form of interaction [38]. We therefore also assessed multiplicative interaction. We calculated the HR of diabetes in relation to combinations of metabolic signature levels (or smoking status) and GRS status (low, intermediate, or high) and estimated potential additive interaction as the relative excess risk due to interaction (RERI) [39]. We also did the analyses of smoking/metabolic signature and diabetes in different GRS-IR and GRS-T2D subgroups. Potential multiplicative interaction between the metabolic signature (or smoking) and GRS was tested by comparing models with and without the interaction term, using the Likelihood ratio test. We used the same covariates as in the main model, except that we did not adjust for ethnicity or family history of diabetes and included adjustment for genotyping batch and the first 10 genetic principal components. In a sensitivity analysis we excluded participants with at least one relative in UKB.

The elastic net regression and MR analyses were performed in R 4.0.4 and other analyses were performed using STATA 17.0. Significance threshold were set at *p* < 0·05, unless stated otherwise.

## Results

As compared to never smokers, current smokers were younger, more likely to be male, have lower educational attainment and have a family history of diabetes, and consumed less fruit and whole grain and more unprocessed meat, processed meat, refined grain and coffee (Table 1).

**Table 1:** Baseline characteristics of UK Biobank participants by smoking status

Baseline characteristics ^a^	Never smoking	Former smoking	Current smoking
Participants, n (%)	53,381 (56.9)	30,721 (32.7)	9,760 (10.4)
Age in years, mean	55.0 (8.1)	56.9 (7.9)	53.4 (8.0)
Men, %	38.9	45.6	51.2
Self-reported white background, %	94.1	96.7	94.6
Higher education, %	49.7	47.1	41.1
Current alcohol consumer, %	80.0	86.8	79.5
Ideal physical activity ^b^, %	61.2	62.5	59.5
Intake of vegetable oil, %	50.4	47.1	43.0
≥ 3 servings/day of fruit, %	38.6	38.0	23.9
≥ 3 servings/day of vegetables, %	8.0	9.8	8.3
Oily fish intake in times/week, mean	1.1	1.1	1.0
Non-oily fish intake in times/week, mean	1.2	1.2	1.1
≤ 2 servings/week of unprocessed meat, %	69.5	69.2	64.0
≤ 1 serving/week of processed meat, %	70.6	70.6	63.1
Never consuming sugar-sweetened beverages, %	14.0	15.6	14.0
≥ 3 servings/day of whole grain, %	97.2	97.0	94.5
Consuming refined grain at least weekly, %	54.0	53.1	65.5
BMI in kg/m^2^, mean	26.8	27.3	26.5
WHR, mean	0.85	0.87	0.87
Fasting hours, mean	3.7	3.7	4.3
Coffee intake in cups/day, mean	1.8	2.1	2.8
Tea intake in cups/week, mean	3.4	3.4	3.7
Family history of diabetes, %	32.5	32.5	35.5

BMI: body mass index; WHR: waist-to-hip ratio;

^a^ Means or proportions were estimated by adjusting for age and sex for all variables except the number of participants, age and sex

^b^ An ideal level of physical activity is defined as ≥ 150 min/week of moderate or ≥ 75 min/week of vigorous or ≥ 150 min/week of mixed moderate and vigorous activity

### Smoking and individual metabolites

Current smoking was positively associated with 107 metabolites and inversely associated with 101 metabolites as compared to never smoking at baseline (eTable 3–4). The MR analysis confirmed a possible causal relationship for 131 metabolites (“smoking-related metabolites”), including positive associations for 65 and inverse associations for 66 metabolites, respectively. The 65 metabolites increased by smoking included glycoprotein acetyls, fatty acids (total fatty acids, monounsaturated fatty acid [MUFA] and saturated fatty acid [SFA] and their ratios to total fatty acids), levels and average diameters of very-low-density lipoprotein (VLDL), levels of different lipids in VLDL, and triglycerides in different lipoprotein particles (eTable 3). The 66 metabolites decreased by smoking included histidine, fatty acids (degrees of unsaturation, docosahexaenoic acid (DHA), and the percentages of DHA, polyunsaturated fatty acids [PUFA], omega-6 fatty acids, omega-3 fatty acids, and linoleic acid to total fatty acids), levels and average diameters of high-density lipoprotein (HDL) particles, levels of different lipids in HDL, the percentages of different lipids (except triglycerides) to total lipids in VLDL particles, and so on (eTable 4). The metabolites increased by smoking were in general associated with increased diabetes risks in individual analyses while most of the metabolites decreased by smoking were inversely associated with diabetes (eTable 5–6).

As compared to never smokers, former smokers had higher levels of 33 and lower levels of 77 metabolites at baseline, zero and 2 of which were confirmed by repeat assessment, respectively (eTable 7–8). The differences in levels of metabolites between former and never smokers were much smaller than the differences between current and never smokers.

The variance in current smoking-related metabolites explained by smoking status ranged from 0·01% to 1·48% (eFigure 2). BMI, WHR, ethnicity, sex, age, alcohol and oily fish intake, and fasting time also contributed to the variance of these metabolites (eFigure 2). The smoking-metabolites were therefore regressed on these covariates before inclusion in the elastic net regression to create the smoking-related metabolic signature.

### Smoking-related metabolic signature

**Fig. 2:**
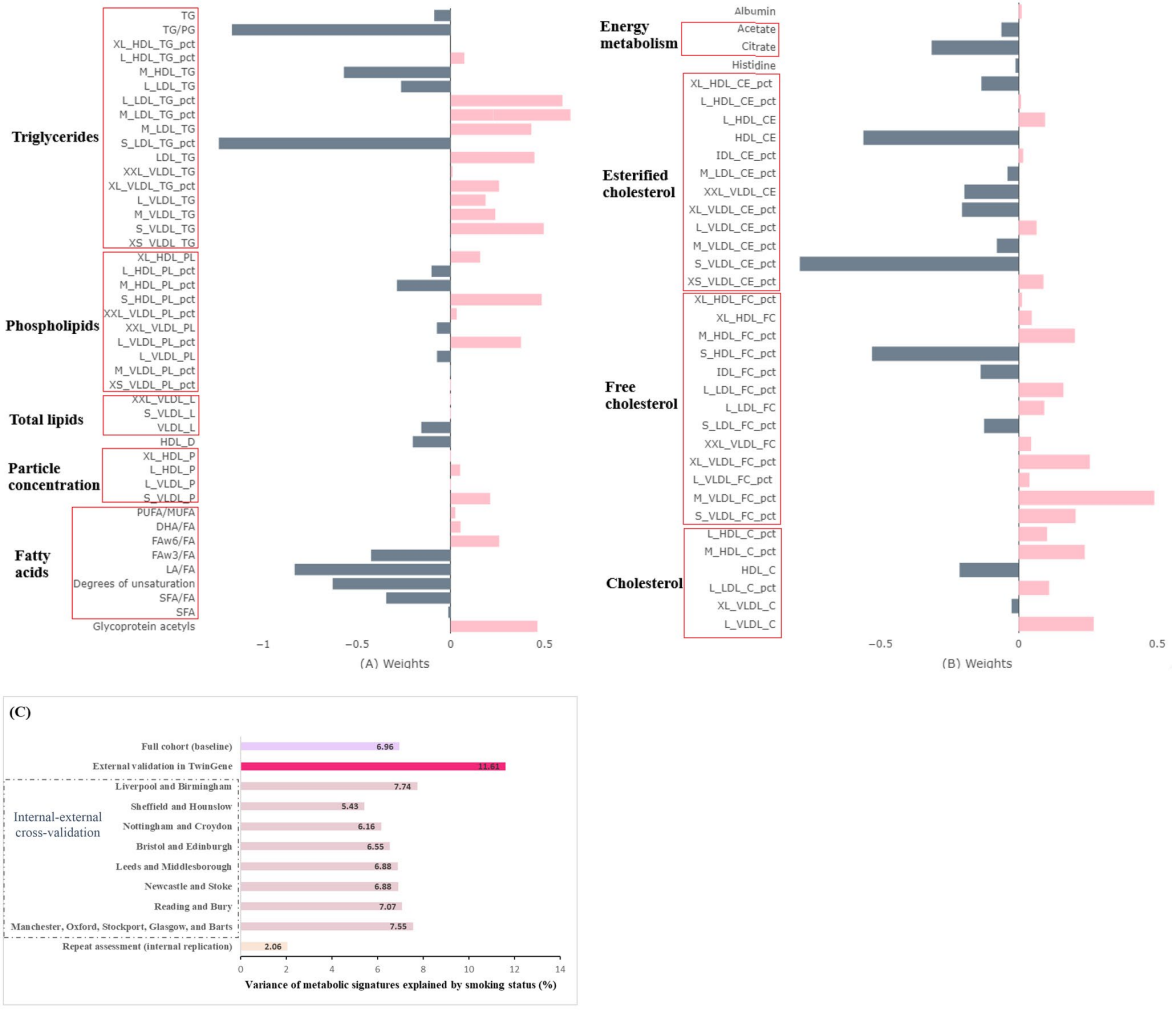
Identification of metabolic signature for current smoking. (**A**) and (**B**) Weights of metabolites contributing to the calculation of the smoking-related metabolic signature in the full cohort; (**C**) Variance of the metabolic signature explained by smoking status in the full cohort and different assessment canters (internal-external cross-validation) at baseline, at repeat assessment (internal replication), and in TwinGene (external validation). Metabolic signatures were calculated as the weighted sum of the metabolites selected by the elastic net regressions with 10-fold cross-validation, with regression coefficients as the weights for corresponding metabolites. DHA: docosahexaenoic acid; DHA/FA: docosahexaenoic acid to fatty acid ratio; FA: fatty acids; FAw3: omega − 3 fatty acids; FAw3/FA: omega − 3 fatty acid to total fatty acid ratio; FAw6: omega − 6 fatty acids; FAw6/FA: omega − 6 fatty acid to total fatty acid ratio; HDL: high-density lipoproteins; HDL_D: high density lipoprotein particle diameter; IDL: intermediate-density lipoproteins; L: large; LA: linoleic acid; LDL: low-density lipoproteins; M: medium; MUFA: monounsaturated fatty acids; PG: phosphoglyceride; PUFA: polyunsaturated fatty acids; PUFA/MUFA: polyunsaturated fatty acid to monounsaturated fatty acid ratio; S: small; SFA: saturated fatty acids; SFA/FA: saturated fatty acid to fatty acids; VLDL: very-low-density lipoproteins; XL: very large; XS: very small; XXL: extremely large; The suffix of “_P” means particle concentrations of lipoproteins; the suffix of “_L” means total lipids in lipoproteins; the suffix of “_PL” means phospholipids in lipoproteins; the suffix of “_TG” means triglycerides; the suffix of “_C” means cholesterol; the suffix of “_CE” means esterified cholesterol; the suffix of “_FC” means free cholesterol; the suffix of “_pct” means percentage of certain lipids to total lipids in lipoproteins

The elastic net regression selected 80 of the 131 smoking-related metabolites to create the smoking-related metabolic signature, including 41 current smoking-raised metabolites and 39 current smoking-decreased metabolites (Fig. 2A-B). Metabolites contributing the most to the metabolic signature included glycoprotein acetyls, free fatty acids (degree of unsaturation, the ratios of linoleic acid and omega-3 fatty acids to total fatty acids), citrate, and some lipids. Such lipids included phospholipids to total lipids percentage in large VLDL, phospholipids to total lipids percentage in small and medium HDL, triglycerides in different lipoproteins, free cholesterol to total lipid percentage in VLDL and HDL particles, and esterified cholesterol to total lipids percentage in VLDL and HDL particles. Smoking status explained 6·96% of the variance in the metabolic signature at baseline and 2·06% at repeat assessment (Fig. 2C). The metabolic signature was externally validated, with 11·61% of variance explained by smoking in TwinGene (Fig. 2C). Across the eight cycles of internal-external cross-validation, smoking explained 5·43% to 7·55% of the variances in the metabolic signatures in assessment centers left out for validation (eTable 9; Fig. 2C).

### Smoking, metabolic signature and incidence of type 2 diabetes

**Fig. 3:**
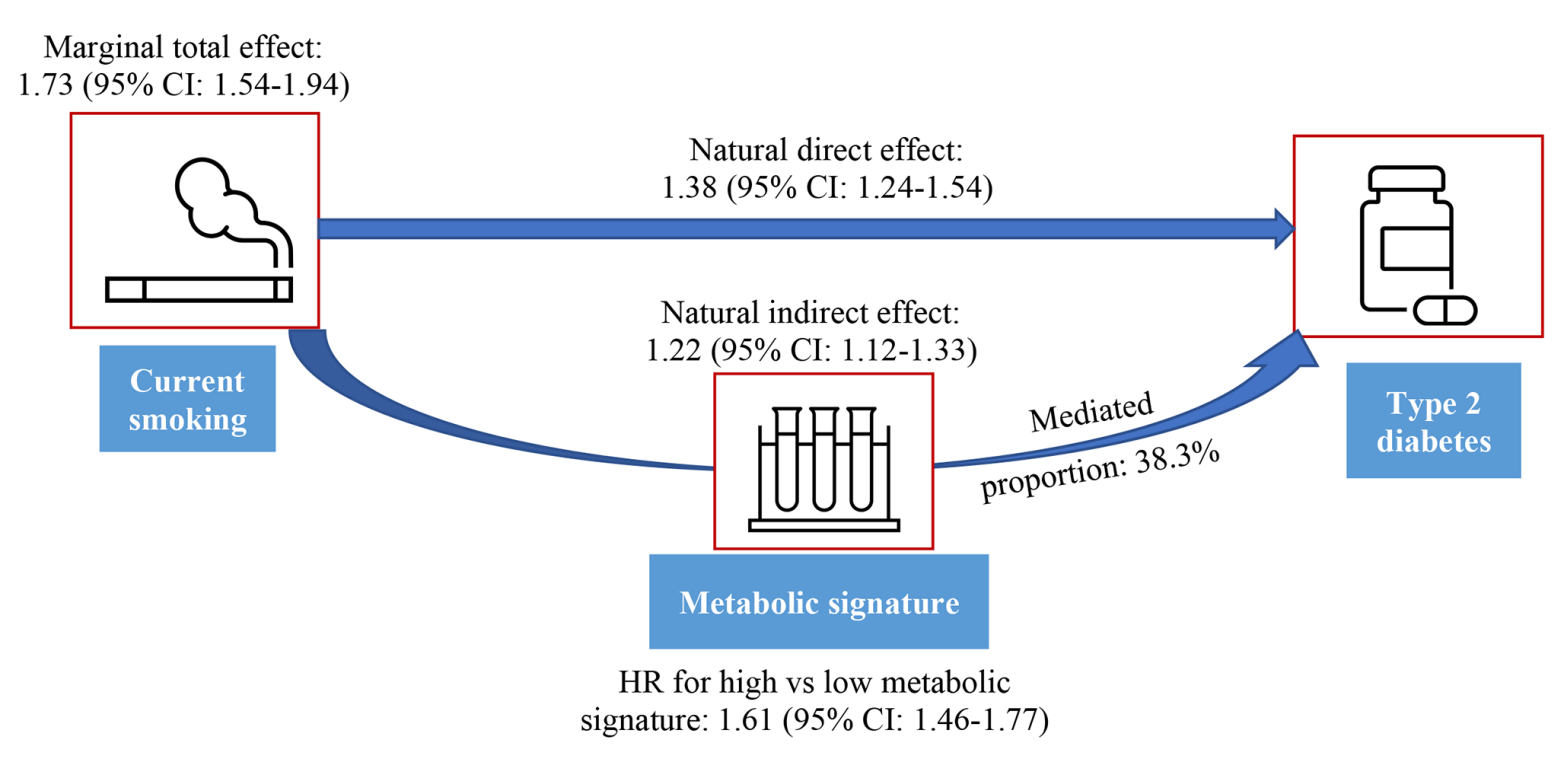
Smoking-type 2 diabetes association mediated by the smoking-related metabolic signature in UK Biobank. HR: hazard ratio; CI: confidence interval. High vs. low metabolic signature: metabolic score > median value vs. metabolic score ≤ median value; Cox models were fitted with attained age as the time scale, and with adjustment for age groups (through stratification), sex (through stratification), assessment center, education, ethnicity, Townsend deprivation index, body mass index, alcohol intake, physical activity, consumption of vegetable oil, oily fish, non-oily fish, coffee, tea, fruits, vegetable, unprocessed meat, processed meat, sugar or foods/drinks containing sugar, whole grain, refined grain, and family history of diabetes

During a median follow-up of 13·0 years, we identified 1869 type 2 diabetes cases. The smoking-related metabolic signature was positively associated with type 2 diabetes, with an HR of 1·31 (95% CI: 1·26 − 1·37) per SD increase in the metabolic signature and an HR of 1·61 (1·46 − 1·77) for high (above the median value) vs. low levels (Fig. 3; eTable 10). Current smokers had a higher incidence of diabetes than never smokers (HR: 1·73, 95% CI: 1·54 − 1·94), and 38·3% of the increased risk was mediated through the metabolic signature (43·6% in men and 30·4% in women (eTable 10). The HR estimated for the natural direct effect of smoking on diabetes was 1·38 (95% CI: 1·24 − 1·54; Fig. 3). In the validation analyses in TwinGene, per SD increase in the metabolic signature was associated with 26% increased incidence of type 2 diabetes, mediating 55·1% of the adverse effect of smoking (eTable 11).

### Interaction with genetic susceptibility

Individuals with the combination of a high level on the metabolic signature and a high GRS-T2D had a HR of 3·18 (95% CI: 2·46 − 4·12) compared to those with low levels of the metabolic signature and the GRS-T2D, with evidence of additive interaction (RERI 0·81, 95% CI: 0·23 − 1·38) (Table 2). There was also additive interaction between the metabolic signature and the GRS-IR (RERI 0·47, 95% CI: 0·02 − 0·92). Additive interaction was also observed between smoking status and the GRS-T2D but not with the GRS-IR (eFigure 3). Both smoking and the metabolic signature were associated with type 2 diabetes across strata of the GRS-IR and GRS-T2D, without evidence of multiplicative interaction (eFigure 4–5). The results were similar after excluding participants with at least one relative in the UKB study (eFigure 6–7).

**Table 2:** Joint analysis of type 2 diabetes in relation to different combinations of smoking-related metabolic signature and genetic susceptibility in UK Biobank

Genetic risk scores	Levels of metabolic signature	Cases	HR (95% CI)	RERI (95% CI)
**GRS-IR**				
Low	low	111	Reference	
Intermediate	low	363	1.06 (0.85–1.31)	
High	low	125	1.22 (0.95–1.58)	
Low	high	172	1.48 (1.16–1.88)	
Intermediate	high	609	1.79 (1.46–2.19)	0.25 (-0.06, 0.57)
High	high	226	2.18 (1.74–2.75)	0.47 (0.02, 0.92)
**GRS-T2D**				
Low	low	79	Reference	
Intermediate	low	316	1.31 (1.02–1.67)	
High	low	138	1.82 (1.38–2.40)	
Low	high	129	1.52 (1.14–2.01)	
Intermediate	high	509	2.12 (1.67–2.69)	0.31 (-0.08, 0.69)
High	high	241	3.18 (2.46–4.12)	0.81 (0.23, 1.38)

GRS_IR: genetic risk score for insulin resistance; GRS_T2D: genetic risk score for type 2 diabetes; HR: hazard ratio; CI: confidence interval; RERI: relative excess risk due to interaction (additive)

Cox models were fitted with attained age as the time scale, with adjustment for age groups (through stratification), sex (through stratification). genetic batch, the first 10 genetic principal components, assessment center, education, Townsend deprivation index, body mass index, alcohol intake, physical activity, and consumption of vegetable oil, oily fish, non-oily fish, coffee, tea, fruits, vegetable, unprocessed meat, processed meat, sugar or foods/drinks containing sugar, whole grain, refined grain

## Discussion

### Main findings

Based on large-scale metabolomics data in UKB, we identified a wide range of metabolites causally affected by smoking, including an inflammatory biomarker, fatty acids, and different lipids subclasses. Most of these metabolites were associated with type 2 diabetes, and when we aggregated them into a smoking-related metabolic signature, this signature mediated more than one third of the association between smoking and type 2 diabetes. The metabolic signature was confirmed by external validation, and it also mediated part of the smoking-diabetes association in the TwinGene study. We also observed additive interaction between the metabolic signature and genetic susceptibility to type 2 diabetes or insulin resistance. This implies that smoking increases the risk of type 2 diabetes in part through effects on the metabolome and suggests that such effects are more pronounced in individuals with genetic risk factors for diabetes.

### Comparison with previous studies

Our finding that smoking affects a variety of metabolites confirms those of previous, smaller observational studies [4–13] (eTable 12) and a one-sample MR-study [15]. The observation that most of them also associate with incidence of type 2 diabetes is consistent with previous prospective studies [19, 40, 41]. For free fatty acids, we observed a general pattern where smoking seemed to decrease the degrees of unsaturation, leading to higher levels of saturated (SFA) and monounsaturated fatty acids (MUFA), and lower percentages of polyunsaturated ones such as DHA, omega-6 fatty acids, and omega-3 fatty acids. Previous studies find that smoking is positively associated with triglycerides and LDL cholesterol [42], and inversely associated with HDL cholesterol [43] and HDL particle sizes. We extend these observations by showing that smoking was associated with larger VLDL particle sizes, higher levels of VLDL regardless different particle sizes, higher levels of triglycerides regardless of types/particle sizes of lipoproteins, lower levels of all forms of cholesterols (total cholesterol, free cholesterol, or esterified cholesterol) in HDL regardless of particle sizes, and lower levels of different forms of cholesterols in IDL. We and others [5, 12, 44] observed associations between smoking and some amino acids but since our MR analyses did not confirm most of them, they are probably not causal. Importantly, our findings in former smokers indicated that most smoking-related metabolic changes are reversible after smoking cessation. The exact biological pathways linking smoking to metabolic changes warrant deeper exploration. Notably, the effect of smoking on gut microbiota has been observed [45] and gut microbiota is an important determinant of metabolite levels [14, 46].

Our study is the first to quantify the overall mediation role of smoking-related alterations of the metabolome in the association between smoking and type 2 diabetes. We did this by integrating multiple metabolites influenced by smoking into one metabolic signature. Our findings suggest that variation in this signature explains 38·3% of the excess risk of type 2 diabetes conferred by smoking. More than half of the smoking-diabetes association was not mediated by the smoking-related metabolic signature, indicating that other pathophysiological consequences of smoking play a role in diabetes development. Such mechanisms may include direct adverse effects of smoking on pancreatic tissue and β-cell function [47]. Part of the effects of smoking on type 2 diabetes may also be mediated by metabolites not measured in the NMR platform which primarily targeted lipid-related metabolites. This remains to be investigated. Nevertheless, non-biological factors such as misclassification of smoking status and measurement errors of the metabolites may also affect the estimated proportion and therefore the exact proportion should be interpreted with caution.

Diabetes is a heterogenous metabolic disorder typically caused by the combination of insulin resistance and insulin deficiency [48]. Several of the smoking associated metabolites are known to be associated with insulin resistance. As an example, glycoprotein acetyls is an inflammatory biomarker and inflammation is an important promotor of insulin resistance [49]. Inflammation and insulin resistance may exacerbate each other [18]. Triglycerides, HDL cholesterol, and free fatty acids are also closely linked to insulin resistance [17, 50]. Metabolites can regulate insulin sensitivity directly by modulating components of the insulin signaling pathway, indirectly by altering the flux of substrates through multiple metabolic pathways such as lipogenesis and lipid oxidation and protein synthesis, and though post-translational modification of proteins [17]. The mediating role of the metabolome in the association between smoking and type 2 diabetes thus seems to involve insulin resistance-related pathways. This is consistent with experimental studies showing that smoking causes insulin resistance [16]. Interestingly, there was additive interaction between the metabolic signature and GRS-IR but not between smoking and GRS-IR. Given the close relationship between smoking-related metabolites and insulin resistance, it is possible that GRS-IR interacts specifically with the metabolic alterations caused by smoking (the metabolic signature) and not with other effects of smoking. The interaction between metabolic signature and GR-IR may reflect synergistic effects of inherited and acquired insulin resistance in the development of type 2 diabetes. Regarding GRS-T2D, we observed additive interaction with both smoking and the metabolic signature. For smoking, previous studies either did not investigate interaction on the additive scale [20] or did not find evidence of additive interaction [51, 52], which might be due to limited statistical power. GRS-T2D primarily captures β-cell function and insulin secretion [53, 54] while the metabolic signature mainly encompassed indicators of insulin resistance. Therefore, our findings indicated that individuals with inherited tendency towards dysfunctional insulin secretion may be more susceptible to adverse effects of acquired insulin resistance. Of note, interaction was detected solely on the additive scale and not on both additive and multiplicative scales, which is considered the strongest form of interaction. Further studies on the topic are clearly warranted.

### Strengths and limitations

The strengths of this study include the use of large-scale metabolomics data and the integration of both observational and MR analyses which allowed us to identify metabolites influenced by smoking. We also had access to genomic information and could, for the first time, investigate if smoking induced alterations of the metabolome interacts with genetic susceptibility on diabetes incidence. We used elastic net regression to derive an overall smoking-related metabolic signature for mediation analysis. Such a model works well for data with high collinearity and has been used by previous metabolomics studies [32, 55]. The robustness and generalizability of the metabolic signature was supported by external validation in TwinGene and internal cross-validation in UKB. A further strength was the ability to adjust for potential confounding from a wide range of lifestyle factors including diet. Both smoking status and metabolite levels may change during the follow-up, but this is most likely to underestimate the associations of smoking and the metabolite signature with diabetes. Furthermore, we confirmed the metabolites identified at baseline by MR analyses and many of the smoking-related metabolites were also associated with current smoking at repeat assessment. MR analyses assume no pleiotropy. We accounted for this issue by applying the MR pleiotropy residual sum and outlier approach (MR-PRESSO) estimator to detect and correct for potential pleiotropy. This study was conducted in people of primarily European origin, and it remains to be explored if our findings are generalizable to non-European populations.

## Conclusions

We find that smoking affects a wide range of metabolites, and these metabolic changes seem to mediate part of the excess risk of type 2 diabetes observed in smokers. It also appears that individuals with genetic susceptibility to insulin resistance or type 2 diabetes are particularly susceptible to such metabolic alterations. Our findings provide insights into how smoking impacts the development of diabetes and emphasizes the significance of refraining from or quitting smoking to prevent diabetes, particularly for individuals with a high genetic risk. Further studies are needed to determine to what extent the metabolic effects we observed in relation to smoking also contributes to other adverse health effects resulting from smoking.

### Electronic supplementary material

Below is the link to the electronic supplementary material.

Supplementary Material 1

## Declarations

### Data sharing statement

UK Biobank data are available through application to the database https://www.ukbiobank.ac.uk/. Data from the Swedish Twin Register is an international resource and can be applied for at https://ki.se/en/research/swedish-twin-registry-for-researchers.
