# Supplementary material for: Metabolic profiling of smoking, associations with type 2 diabetes and interaction with genetic susceptibility
Source: Eur J Epidemiol. 2024 Mar 31;39(6):667–78. doi: 10.1007/s10654-024-01117-5 (PMC11249521; doi:10.1007/s10654-024-01117-5)
Supplement: Supplementary file 1 — Supplementary Material 1 [file 10654_2024_1117_MOESM1_ESM.docx]

**Table of Contents**

**eMethods**

eMethod 1. Two-sample Mendelian randomization (MR) analysis

eMethod 2. External validation of smoking-related metabolic signature in the TwinGene study

eMethod 3. Internal-external cross-validation of smoking-related metabolic signature in UK Biobank

**eTables**

eTable 1. SNPs and weights used in the calculation of genetic susceptibility to insulin resistance or type 2 diabetes.

eTable 2. Detailed information about the 94 SNPs for smoking in the two-sample MR analysis

eTable 3. Metabolites positively associated with current smoking at baseline.

eTable 4. Metabolites inversely associated with current smoking at baseline.

eTable 5. Associations of individual metabolites increased by smoking with type 2 diabetes.

eTable 6. Associations of individual metabolites decreased by smoking with type 2 diabetes.

eTable 7. Metabolites positively associated with former smoking at baseline.

eTable 8. Metabolites inversely associated with former smoking at baseline.

eTable 9. Metabolites and corresponding coefficients estimated by elastic net regression in the full cohort and internal-external cross-validation.

eTable 10. HR (95% CI) of type 2 diabetes in relation to smoking and smoking-related metabolic signature in UK Biobank

eTable 11. HR (95% CI) of type 2 diabetes in relation to smoking and smoking-related metabolic signature in TwinGene.

eTable 12. Summary of previous observational studies on smoking and metabolites based on metabolomics data.

**eFigures**

eFigure 1. Conceptual figure for the role of smoking, metabolic signature on the risk of type 2 diabetes and the interaction with genetic susceptibility

eFigure 2. Variance in 131 smoking-related metabolites explained by smoking status and other factors

eFigure 3. Joint analysis of type 2 diabetes in relation to different combinations of smoking status and genetic susceptibility in UK Biobank

eFigure 4. Smoking status and type 2 diabetes incidence by genetic susceptibility subgroups in UK Biobank

eFigure 5. Smoking-related metabolic signature and type 2 diabetes incidence by genetic susceptibility subgroups in UK Biobank

eFigure 6. Joint analysis of type 2 diabetes in relation to different combinations of metabolic signature and genetic susceptibility in unrelated participants in UK Biobank

eFigure 7. Smoking-related metabolic signature and type 2 diabetes incidence by genetic susceptibility subgroups in unrelated participants in UK Biobank

**eMethod 1. Two-sample Mendelian randomization (MR) analysis**

A two-sample MR study uses genetic variants as instrumental variables (IVs) for the exposure to make cause inference, based on summary statistics for both the exposure and the outcome from genome-wide association studies (GWAS). A previous European-ancestry meta-analysis identified 99 loci (*p*≤5×10^-8^) for smoking initiation (ever versus never smoking) based on 842,717 participants from the Million Veteran Program and the GWAS & Sequencing Consortium of Alcohol and Nicotine use^[1]^. We removed 5 SNPs in linkage disequilibrium (LD) (r^2^≥0.01 within 10,000 kb window) with other SNPs through LD clumping, leading to 94 remaining SNPs as instrumental variable (IVs) for smoking initiation in the MR analysis. Summary statistics for the associations between the 94 SNPs and metabolites obtained from a recent genome-wide association study (GWAS) based on metabolomics data in UK Biobank. The inverse- variance weighted (IVW)^[2]^ method, which assumes that all the IVs are valid, was used as the main MR estimator. The fundamental MR assumption is the ‘no horizontal pleiotropy’ assumption, requiring that the IVs should not affect the outcome directly or through a pathway not involving the exposure^[3]^. The MR pleiotropy residual sum and outlier approach (MR-PRESSO)^[3]^ helps to detect and correct for such potential pleiotropy. We therefore also used the MR-PRESSO estimator. The estimate from the IVW methods was reported if no outlier (pleiotropy) was reported, otherwise outlier-corrected estimate from the MR-PRESSO method was reported.

**eMethod 2. External validation of smoking-related metabolic signature in the TwinGene study.**

The TwinGene study is a cohort nested within the Swedish Twin Register, a nationwide register including over 194,000 twins in Sweden^[4]^. In TwinGene, more than 12,000 individuals born before 1958 participated in a telephone screening in 1998-2002, with information on smoking, alcohol, BMI, and physical activity collected. Smoking status was recorded as never smoking (n=2305), occasional smoking (n=3054), previously regular smoking (n=3334), or current regular smoking (n=1517)^[5]^. The participants were contacted again in 2004-2008 for blood sampling after an overnight fast^[4]^. Metabolomics data were measured in the nuclear magnetic resonance (NMR)-based platform. In this study, we included individuals reporting as never or current regular smokers, with complete data on the metabolites used to calculate the smoking-related metabolic signature, without diabetes at the time of blood sampling, and without missing data on body mass index (BMI), leading to a final sample size of 3626.

Metabolites deviating from normal distribution were log-transformed and we regressed each metabolite on age, sex, BMI, and alcohol intake to obtained standardized residuals of metabolites. The metabolic signature in TwinGene was calculated as a weighted sum of the standardized residuals of the metabolites which were selected by the elastic net regression at baseline in the UK Biobank study. The regression coefficients obtained from UK Biobank were used as the weights. We estimated the variance of the metabolic signature explained by smoking status by regressing the metabolic signature on smoking status in TwinGene.

The 3626 TwinGene participants were followed-up for the occurrence of diabetes through linkage to the Swedish National Patient register and for vital status through linkage with the Causes-of-Death Register until the end of 2014. We fitted Cox proportional hazards regression models to estimate the risk of type 2 diabetes in relation to smoking and the smoking-related metabolic signature, with attained age as the timescale, with adjustment for sex, education, BMI, alcohol, and physical activity. Cluster-robust standard errors were applied to account for correlation within twin pairs. The proportion of smoking-diabetes association mediated by the metabolic signature was also calculated.

**eMethod 3. Internal-external cross-validation of smoking-related metabolic signature in UK Biobank**

The internal-external cross-validation approach combines model training with validation and is used to test for the reproducibility (internal validation) and generalizability (external validation) of prediction models in large datasets comprising of several sub-studies^[6,7]^. Typically, in each cycle of internal-external cross-validation, one of the sub-studies was omitted from the training set and used for external validation^[6]^. This approach helps to identify potential heterogeneity across sub-studies^[6]^ and reveals how the developed models are likely to perform in new data, independent of that used for model training^[7]^. In the current study, we performed eight cycles of internal-external cross-validation in UK Biobank, by leaving some assessment centers out from the training set and treating them as the validation set in each cycle (**see Table below**). To increase the comparability across the eight cycles, we made sure that the sample sizes for the validation sets were in general equal when selecting assessment centers to be left out each time.

We applied the elastic net regression model with 10-fold cross-validation in the training set and calculated the smoking-related metabolic signature in the validation set as a linear combination of metabolites identified from the training model, with corresponding regression coefficients as the weights. We then estimated and compared the variance in the metabolic signatures explained by smoking status in the validation set across the eight cycles of internal-external cross-validation.

**Table. Assessment centers left out for external validation in each cycle in UK Biobank**

| **Cycle** | **Assessment centers lelf-out for external validation** | **Sample size of the validation set** |
| --- | --- | --- |
| 1 | Reading and Bury | 7,435 |
| 2 | Newcastle and Stoke | 6,805 |
| 3 | Leeds and Middlesborough | 8,205 |
| 4 | Bristol and Edinburgh | 7,205 |
| 5 | Nottingham and Croydon | 7,105 |
| 6 | Sheffield and Hounslow | 7,164 |
| 7 | Liverpool and Birmingham | 6,960 |
| 8 | Manchester, Oxford, Stockport, Glasgow, and Barts | 9,715 |

**eTable 1. SNPs and weights used in the calculation of genetic susceptibility to insulin resistance or type 2 diabetes**

| **SNP** | **Effect allele** | **Weight** | **Nearest gene** | **Proxy SNP ^a^** |
| --- | --- | --- | --- | --- |
| **Insulin resistance** |  |  |  |  |
| rs459193 | G | 0.012 | ANKRD55 |  |
| rs13389219 | C | 0.012 | *GRB14* | rs1128249 |
| rs2943641 | C | 0.009 | IRS1 |  |
| rs12970134 | A | 0.008 | MC4R |  |
| rs1801282 | C | 0.016 | PPARG |  |
| **Type 2 diabetes** |  |  |  |  |
| rs10401969 | C | 0.122217633 | SUGP1/CILP2 | rs58542926 |
| rs10842994 | C | 0.09531018 | KLHDC5 |  |
| rs10923931 | T | 0.122217633 | NOTCH2 |  |
| rs1153188 | A | 0.076961041 | DCD |  |
| rs11634397 | G | 0.058268908 | ZFAND6 |  |
| rs11708067 | A | 0.113328685 | ADCY5 |  |
| rs12571751 | A | 0.076961041 | ZMIZ1 |  |
| rs12779790 | G | 0.104360015 | CDC123/CAMK1D | rs11257655 |
| rs12970134 | A | 0.076961041 | MC4R |  |
| rs13266634 | C | 0.139761942 | SLC30A8 |  |
| rs13292136 | C | 0.104360015 | CHCHD2P9 |  |
| rs13389219 | C | 0.067658648 | GRB14 | rs58542926 |
| rs1387153 | T | 0.086177696 | SNRPGP16/MTNR1B |  |
| rs1531343 | C | 0.09531018 | HMGA2 |  |
| rs1552224 | A | 0.131028262 | ARAP1 (CENTD2) |  |
| rs17036101 | G | 0.139761942 | SYN2/GSTM5P1 |  |
| rs2191349 | T | 0.058268908 | GTF3AP5 |  |
| rs231362 | G | 0.076961041 | KCNQ1 |  |
| rs243021 | A | 0.076961041 | MIR4432HG |  |
| rs2796441 | G | 0.067658648 | TLE1 |  |
| rs340874 | C | 0.067658648 | PROX1 |  |
| rs4457053 | G | 0.076961041 | ZBED3 |  |
| rs459193 | G | 0.076961041 | ANKRD55 |  |
| rs4607103 | C | 0.086177696 | ADAMTS9-AS2 |  |
| rs516946 | C | 0.086177696 | ANK1 |  |
| rs7177055 | A | 0.076961041 | HMG20A |  |
| rs7202877 | T | 0.113328685 | BCAR1 |  |
| rs7578326 | A | 0.104360015 | LOC646736 |  |
| rs7578597 | T | 0.139761942 | THADA |  |
| rs780094 | C | 0.058268908 | GCKR |  |
| rs7903146 | T | 0.336472237 | TCF7L2 |  |
| rs7957197 | T | 0.067658648 | OASL/TCF1/HNF1A |  |
| rs7961581 | C | 0.086177696 | TSPAN/LGR5 |  |
| rs8042680 | A | 0.067658648 | PRC1 |  |
| rs864745 | T | 0.09531018 | JAZF1 |  |
| rs896854 | T | 0.058268908 | TP53INP1 |  |
| rs9472138 | T | 0.058268908 | VEGFA/LINC02537 |  |
| rs972283 | G | 0.067658648 | H4P1/KLF14 |  |

^a^ Only for SNPs which were not genotyped in UK Biobank.

**eTable 2. Detailed information about the 94 SNPs for smoking in the two-sample MR analysis**

| **SNP** | **Effect allele** | **Other allele** | **SNPs' associations with smoking** | | | **F statistic** |
| --- | --- | --- | --- | --- | --- | --- |
|  |  |  | **BETA** | **SE** | **p** |  |
| rs1004787 | A | G | 0.0336 | 0.0035 | 6.93E-22 | 92 |
| rs10211770 | T | C | 0.0213 | 0.0037 | 5.21E-09 | 33 |
| rs10233018 | G | A | 0.0283 | 0.0035 | 4.64E-16 | 65 |
| rs1029984 | T | G | 0.0200 | 0.0035 | 1.19E-08 | 33 |
| rs10446671 | T | C | 0.0206 | 0.0035 | 3.06E-09 | 35 |
| rs10493500 | A | G | 0.0256 | 0.0043 | 3.74E-09 | 35 |
| rs10922907 | A | T | 0.0228 | 0.0035 | 6.69E-11 | 42 |
| rs11012750 | G | A | 0.0244 | 0.0038 | 7.49E-11 | 41 |
| rs11057005 | A | G | 0.0196 | 0.0035 | 2.13E-08 | 31 |
| rs11125335 | C | T | 0.0241 | 0.0035 | 1.11E-11 | 47 |
| rs11165531 | T | G | 0.0204 | 0.0035 | 4.65E-09 | 34 |
| rs11191487 | G | A | 0.0390 | 0.0048 | 6.11E-16 | 66 |
| rs113617088 | A | G | 0.0302 | 0.0055 | 3.32E-08 | 30 |
| rs11581459 | A | T | 0.0293 | 0.0036 | 1.62E-16 | 66 |
| rs11611651 | A | G | 0.0355 | 0.0062 | 1.01E-08 | 33 |
| rs11720703 | T | C | 0.0198 | 0.0035 | 1.23E-08 | 32 |
| rs11768481 | C | A | 0.0256 | 0.0037 | 2.96E-12 | 48 |
| rs12027999 | T | C | 0.0300 | 0.0053 | 1.26E-08 | 32 |
| rs12186738 | G | T | 0.0278 | 0.0049 | 1.39E-08 | 32 |
| rs12202507 | C | T | 0.0205 | 0.0036 | 8.50E-09 | 32 |
| rs12315033 | A | T | 0.0541 | 0.0092 | 3.34E-09 | 35 |
| rs12474587 | T | G | 0.0250 | 0.0035 | 1.12E-12 | 51 |
| rs12530388 | A | C | 0.0190 | 0.0035 | 3.42E-08 | 29 |
| rs12545053 | G | A | 0.0222 | 0.0036 | 4.20E-10 | 38 |
| rs12632110 | A | G | 0.0237 | 0.0037 | 1.16E-10 | 41 |
| rs12708665 | G | A | 0.0219 | 0.0038 | 1.06E-08 | 33 |
| rs1343432 | C | G | 0.0231 | 0.0041 | 1.49E-08 | 32 |
| rs1346732 | T | A | 0.0234 | 0.0039 | 2.82E-09 | 36 |
| rs1385108 | T | C | 0.0245 | 0.0041 | 2.23E-09 | 36 |
| rs1392510 | C | T | 0.0278 | 0.0050 | 3.10E-08 | 31 |
| rs1518393 | C | A | 0.0196 | 0.0036 | 3.84E-08 | 30 |
| rs1565735 | T | A | 0.0403 | 0.0044 | 2.25E-20 | 84 |
| rs17055603 | G | A | 0.0208 | 0.0038 | 3.77E-08 | 30 |
| rs1901477 | G | A | 0.0289 | 0.0036 | 1.24E-15 | 64 |
| rs1945735 | A | G | 0.0228 | 0.0040 | 1.44E-08 | 32 |
| rs2218439 | A | G | 0.0242 | 0.0035 | 5.78E-12 | 48 |
| rs2237235 | A | G | 0.0228 | 0.0042 | 4.96E-08 | 29 |
| rs2378662 | A | G | 0.0201 | 0.0035 | 9.07E-09 | 33 |
| rs238896 | G | A | 0.0196 | 0.0035 | 1.67E-08 | 31 |
| rs2398737 | T | G | 0.0244 | 0.0035 | 2.85E-12 | 49 |
| rs2435211 | T | C | 0.0223 | 0.0038 | 4.40E-09 | 34 |
| rs2652430 | G | A | 0.0218 | 0.0035 | 4.14E-10 | 39 |
| rs281287 | G | A | 0.0275 | 0.0036 | 3.75E-14 | 58 |
| rs2865303 | C | G | 0.0293 | 0.0048 | 1.41E-09 | 37 |
| rs28717373 | C | T | 0.0263 | 0.0036 | 3.04E-13 | 53 |
| rs3001723 | A | G | 0.0315 | 0.0038 | 1.55E-16 | 69 |
| rs301805 | G | T | 0.0210 | 0.0035 | 2.81E-09 | 36 |
| rs34121288 | T | C | 0.0197 | 0.0036 | 4.92E-08 | 30 |
| rs34626694 | T | C | 0.0208 | 0.0037 | 2.49E-08 | 32 |
| rs36072649 | T | A | 0.0219 | 0.0036 | 1.06E-09 | 37 |
| rs36096261 | A | G | 0.0312 | 0.0054 | 6.27E-09 | 33 |
| rs3741499 | C | T | 0.0205 | 0.0037 | 2.03E-08 | 31 |
| rs3843409 | T | C | 0.0201 | 0.0036 | 2.13E-08 | 31 |
| rs4044321 | A | G | 0.0289 | 0.0036 | 2.15E-15 | 64 |
| rs40590 | G | C | 0.0216 | 0.0035 | 6.08E-10 | 38 |
| rs4255357 | A | G | 0.0228 | 0.0035 | 5.92E-11 | 42 |
| rs4523689 | A | G | 0.0205 | 0.0036 | 8.89E-09 | 32 |
| rs4543592 | C | T | 0.0193 | 0.0035 | 2.42E-08 | 30 |
| rs455650 | C | T | 0.0388 | 0.0045 | 4.05E-18 | 74 |
| rs4571506 | C | T | 0.0227 | 0.0035 | 8.81E-11 | 42 |
| rs4766578 | T | A | 0.0200 | 0.0035 | 9.52E-09 | 33 |
| rs491283 | C | G | 0.0206 | 0.0036 | 9.10E-09 | 33 |
| rs5762749 | C | G | 0.0207 | 0.0036 | 8.57E-09 | 33 |
| rs6119893 | T | G | 0.0238 | 0.0037 | 1.22E-10 | 41 |
| rs61959481 | G | A | 0.0236 | 0.0043 | 3.11E-08 | 30 |
| rs62025923 | T | C | 0.0282 | 0.0043 | 6.04E-11 | 43 |
| rs6265 | C | T | 0.0344 | 0.0045 | 1.29E-14 | 58 |
| rs6438208 | G | A | 0.0251 | 0.0040 | 2.93E-10 | 39 |
| rs6499255 | G | A | 0.0268 | 0.0046 | 4.69E-09 | 34 |
| rs6508144 | C | G | 0.0199 | 0.0035 | 1.83E-08 | 32 |
| rs66632973 | A | T | 0.0281 | 0.0043 | 4.47E-11 | 43 |
| rs6728726 | C | T | 0.0333 | 0.0046 | 6.05E-13 | 52 |
| rs6756212 | C | T | 0.0391 | 0.0035 | 2.90E-29 | 125 |
| rs6948707 | G | T | 0.0236 | 0.0035 | 2.06E-11 | 45 |
| rs7005565 | C | T | 0.0236 | 0.0039 | 1.52E-09 | 37 |
| rs7083526 | T | C | 0.0249 | 0.0035 | 9.10E-13 | 51 |
| rs7110863 | G | A | 0.0443 | 0.0036 | 1.57E-35 | 151 |
| rs7197072 | C | T | 0.0237 | 0.0041 | 7.06E-09 | 33 |
| rs74373890 | C | G | 0.0488 | 0.0085 | 1.14E-08 | 33 |
| rs748832 | G | A | 0.0205 | 0.0036 | 1.17E-08 | 32 |
| rs7505855 | C | T | 0.0203 | 0.0035 | 8.94E-09 | 34 |
| rs7553158 | G | A | 0.0206 | 0.0035 | 5.38E-09 | 35 |
| rs77464064 | T | C | 0.0407 | 0.0070 | 5.60E-09 | 34 |
| rs7787612 | C | T | 0.0233 | 0.0040 | 6.64E-09 | 34 |
| rs7790430 | G | C | 0.0211 | 0.0036 | 5.99E-09 | 34 |
| rs7829715 | T | C | 0.0250 | 0.0035 | 5.69E-13 | 51 |
| rs7901883 | G | A | 0.0264 | 0.0041 | 2.14E-10 | 41 |
| rs7969559 | A | G | 0.0247 | 0.0039 | 1.57E-10 | 40 |
| rs7993498 | C | T | 0.0218 | 0.0035 | 4.72E-10 | 39 |
| rs9375328 | C | T | 0.0265 | 0.0039 | 1.26E-11 | 46 |
| rs9517908 | G | A | 0.0251 | 0.0043 | 4.27E-09 | 34 |
| rs9540731 | C | T | 0.0205 | 0.0035 | 3.99E-09 | 34 |
| rs9922607 | C | T | 0.0238 | 0.0044 | 4.64E-08 | 29 |
| rs993700 | T | C | 0.0261 | 0.0042 | 4.21E-10 | 39 |

SNP: single nucleotide polymorphism; MR: Mendelian randomization; SE: standard error

.

**eTable 3. Metabolites positively associated with current smoking at baseline**

| **Metabolites** | **Baseline analysis ^a^** | | | |  | **Repeat assessment ^a^** | | | |  | **MR analysis ^b^** | | |
| --- | --- | --- | --- | --- | --- | --- | --- | --- | --- | --- | --- | --- | --- |
|  | **Sample size** | **β** | **SE** | ***p*** |  | **Sample size** | **β** | **SE** | ***p*** |  | **No. of SNPs** | **β (95% CI)** | ***p*** |
| **Inflammation** |  |  |  |  |  |  |  |  |  |  |  |  |  |
| Glycoprotein Acetyls | 93722 | 0.301 | 0.010 | <0.001 |  | 3797 | 0.372 | 0.081 | <0.001 |  | 94 | 0.107 (0.06, 0.153) | <0.001 |
| **Fatty acids** |  |  |  |  |  |  |  |  |  |  |  |  |  |
| MUFA | 93659 | 0.218 | 0.010 | <0.001 |  | 3796 | 0.255 | 0.079 | 0.001 |  | 91 | 0.079 (0.035, 0.122) | 0.001 |
| MUFA/FA | 93659 | 0.340 | 0.009 | <0.001 |  | 3796 | 0.319 | 0.073 | <0.001 |  | 92 | 0.145 (0.096, 0.194) | <0.001 |
| SFA | 93659 | 0.136 | 0.010 | <0.001 |  | 3796 | 0.188 | 0.078 | 0.016 |  | 94 | 0.065 (0.019, 0.111) | 0.006 |
| SFA/FA | 93659 | 0.156 | 0.011 | <0.001 |  | 3796 | 0.156 | 0.078 | 0.045 |  | 93 | 0.111 (0.065, 0.158) | <0.001 |
| FA | 93659 | 0.111 | 0.011 | <0.001 |  | 3796 | 0.169 | 0.080 | 0.034 |  | 92 | 0.057 (0.015, 0.099) | 0.010 |
| FAw6/FAw3 | 93659 | 0.190 | 0.010 | <0.001 |  | 3796 | 0.079 | 0.079 | 0.314 |  | 87 | 0.019 (-0.022, 0.06) | 0.357 |
| **Concentrations of lipoproteins** |  |  |  |  |  |  |  |  |  |  |  |  |  |
| VLDL_P | 93722 | 0.143 | 0.010 | <0.001 |  | 3797 | 0.170 | 0.080 | 0.033 |  | 93 | 0.031 (-0.012, 0.073) | 0.160 |
| XS_VLDL_P | 93722 | 0.110 | 0.010 | <0.001 |  | 3797 | 0.122 | 0.076 | 0.109 |  | 93 | 0.003 (-0.039, 0.045) | 0.897 |
| S_VLDL_P | 93722 | 0.163 | 0.011 | <0.001 |  | 3797 | 0.188 | 0.082 | 0.022 |  | 93 | 0.046 (0.004, 0.088) | 0.033 |
| M_VLDL_P | 93722 | 0.089 | 0.010 | <0.001 |  | 3797 | 0.127 | 0.077 | 0.101 |  | 93 | 0.007 (-0.035, 0.048) | 0.747 |
| L_VLDL_P | 93722 | 0.173 | 0.010 | <0.001 |  | 3797 | 0.186 | 0.078 | 0.017 |  | 94 | 0.06 (0.014, 0.107) | 0.011 |
| XL_VLDL_P | 93722 | 0.181 | 0.010 | <0.001 |  | 3797 | 0.185 | 0.077 | 0.017 |  | 93 | 0.076 (0.029, 0.122) | 0.002 |
| XXL_VLDL_P | 93722 | 0.172 | 0.010 | <0.001 |  | 3797 | 0.143 | 0.078 | 0.068 |  | 93 | 0.079 (0.034, 0.124) | 0.001 |
| LDL_P | 93722 | 0.041 | 0.010 | <0.001 |  | 3797 | 0.094 | 0.076 | 0.216 |  | 93 | -0.028 (-0.068, 0.013) | 0.181 |
| XS_LDL_P | 93722 | 0.061 | 0.010 | <0.001 |  | 3797 | 0.117 | 0.077 | 0.132 |  | 93 | -0.021 (-0.062, 0.02) | 0.314 |
| L_LDL_P | 93722 | 0.042 | 0.010 | <0.001 |  | 3797 | 0.097 | 0.075 | 0.199 |  | 93 | -0.035 (-0.075, 0.006) | 0.095 |
| **Diameters of lipoproteins** |  |  |  |  |  |  |  |  |  |  |  |  |  |
| VLDL_D | 93722 | 0.140 | 0.009 | <0.001 |  | 3797 | 0.159 | 0.072 | 0.026 |  | 94 | 0.071 (0.022, 0.119) | 0.004 |
| **Total lipids** |  |  |  |  |  |  |  |  |  |  |  |  |  |
| VLDL_L | 93722 | 0.162 | 0.010 | <0.001 |  | 3797 | 0.187 | 0.078 | 0.017 |  | 93 | 0.053 (0.009, 0.097) | 0.021 |
| XS_VLDL_L | 93722 | 0.117 | 0.010 | <0.001 |  | 3797 | 0.127 | 0.076 | 0.094 |  | 93 | 0.007 (-0.034, 0.049) | 0.736 |
| S_VLDL_L | 93722 | 0.157 | 0.011 | <0.001 |  | 3797 | 0.184 | 0.082 | 0.025 |  | 93 | 0.044 (0.002, 0.086) | 0.041 |
| M_VLDL_L | 93722 | 0.109 | 0.010 | <0.001 |  | 3797 | 0.141 | 0.078 | 0.072 |  | 92 | 0.026 (-0.015, 0.066) | 0.222 |
| L_VLDL_L | 93722 | 0.163 | 0.010 | <0.001 |  | 3797 | 0.173 | 0.077 | 0.025 |  | 93 | 0.062 (0.016, 0.107) | 0.009 |
| XL_VLDL_L | 93722 | 0.174 | 0.010 | <0.001 |  | 3797 | 0.180 | 0.076 | 0.017 |  | 94 | 0.067 (0.02, 0.114) | 0.005 |
| XXL_VLDL_L | 93722 | 0.155 | 0.010 | <0.001 |  | 3797 | 0.147 | 0.077 | 0.055 |  | 93 | 0.073 (0.028, 0.118) | 0.002 |
| S_LDL_L | 93722 | 0.055 | 0.010 | <0.001 |  | 3797 | 0.099 | 0.077 | 0.200 |  | 93 | -0.016 (-0.058, 0.025) | 0.444 |
| M_LDL_L | 93722 | 0.036 | 0.010 | 0.001 |  | 3797 | 0.097 | 0.078 | 0.212 |  | 93 | 0.002 (-0.04, 0.044) | 0.920 |
| **Phospholipids** |  |  |  |  |  |  |  |  |  |  |  |  |  |
| VLDL_PL | 93722 | 0.158 | 0.010 | <0.001 |  | 3797 | 0.188 | 0.079 | 0.017 |  | 93 | 0.045 (0.001, 0.088) | 0.047 |
| XS_VLDL_PL_pct | 93722 | 0.219 | 0.010 | <0.001 |  | 3797 | 0.109 | 0.074 | 0.143 |  | 91 | 0.08 (0.036, 0.124) | 0.001 |
| XS_VLDL_PL | 93722 | 0.142 | 0.011 | <0.001 |  | 3797 | 0.140 | 0.077 | 0.070 |  | 92 | 0.021 (-0.02, 0.061) | 0.319 |
| S_VLDL_PL | 93722 | 0.109 | 0.010 | <0.001 |  | 3797 | 0.147 | 0.080 | 0.066 |  | 92 | 0.02 (-0.019, 0.059) | 0.322 |
| M_VLDL_PL | 93722 | 0.099 | 0.011 | <0.001 |  | 3797 | 0.136 | 0.083 | 0.101 |  | 93 | 0.003 (-0.038, 0.044) | 0.876 |
| L_VLDL_PL_pct | 93719 | 0.204 | 0.011 | <0.001 |  | 3797 | 0.201 | 0.081 | 0.013 |  | 94 | 0.107 (0.059, 0.155) | 0.000 |
| L_VLDL_PL | 93722 | 0.148 | 0.010 | <0.001 |  | 3797 | 0.177 | 0.077 | 0.022 |  | 94 | 0.067 (0.02, 0.114) | 0.005 |
| XL_VLDL_PL | 93722 | 0.151 | 0.010 | <0.001 |  | 3797 | 0.148 | 0.076 | 0.050 |  | 94 | 0.063 (0.017, 0.11) | 0.008 |
| XL_VLDL_PL_pct | 93090 | 0.092 | 0.011 | <0.001 |  | 3776 | 0.080 | 0.082 | 0.329 |  | 94 | -0.017 (-0.062, 0.028) | 0.463 |
| XXL_VLDL_PL_pct | 90945 | 0.137 | 0.011 | <0.001 |  | 3713 | 0.113 | 0.085 | 0.182 |  | 94 | 0.077 (0.04, 0.113) | 0.000 |
| XXL_VLDL_PL | 93722 | 0.158 | 0.011 | <0.001 |  | 3797 | 0.128 | 0.081 | 0.114 |  | 93 | 0.083 (0.038, 0.128) | 0.000 |
| S_LDL_PL | 93722 | 0.070 | 0.010 | <0.001 |  | 3797 | 0.084 | 0.077 | 0.278 |  | 92 | -0.025 (-0.065, 0.015) | 0.224 |
| S_LDL_PL_pct | 93722 | 0.039 | 0.011 | <0.001 |  | 3797 | -0.094 | 0.081 | 0.250 |  | 94 | -0.059 (-0.101, -0.018) | 0.005 |
| M_LDL_PL | 93722 | 0.035 | 0.011 | 0.001 |  | 3797 | 0.093 | 0.079 | 0.239 |  | 93 | -0.002 (-0.044, 0.041) | 0.942 |
| IDL_PL_pct | 93722 | 0.092 | 0.011 | <0.001 |  | 3797 | 0.130 | 0.081 | 0.108 |  | 93 | 0.008 (-0.031, 0.048) | 0.681 |
| S_HDL_PL_pct | 93722 | 0.037 | 0.011 | 0.001 |  | 3797 | 0.013 | 0.083 | 0.876 |  | 92 | 0.048 (0.007, 0.089) | 0.025 |
| M_HDL_PL_pct | 93722 | 0.249 | 0.010 | <0.001 |  | 3797 | 0.234 | 0.072 | 0.001 |  | 93 | 0.098 (0.05, 0.147) | <0.001 |
| L_HDL_PL_pct | 93722 | 0.117 | 0.009 | <0.001 |  | 3797 | 0.153 | 0.064 | 0.016 |  | 93 | 0.157 (0.111, 0.202) | <0.001 |
| **Triglycerides** |  |  |  |  |  |  |  |  |  |  |  |  |  |
| VLDL_TG | 93722 | 0.180 | 0.010 | <0.001 |  | 3797 | 0.186 | 0.078 | 0.017 |  | 94 | 0.065 (0.019, 0.112) | 0.006 |
| XS_VLDL_TG_pct | 93722 | 0.204 | 0.010 | <0.001 |  | 3797 | 0.180 | 0.070 | 0.010 |  | 89 | 0.108 (0.064, 0.151) | <0.001 |
| XS_VLDL_TG | 93722 | 0.236 | 0.011 | <0.001 |  | 3797 | 0.248 | 0.084 | 0.003 |  | 92 | 0.102 (0.06, 0.143) | <0.001 |
| S_VLDL_TG | 93722 | 0.201 | 0.011 | <0.001 |  | 3797 | 0.200 | 0.083 | 0.015 |  | 92 | 0.086 (0.045, 0.128) | <0.001 |
| S_VLDL_TG_pct | 93722 | 0.147 | 0.010 | <0.001 |  | 3797 | 0.087 | 0.073 | 0.231 |  | 91 | 0.084 (0.041, 0.128) | <0.001 |
| M_VLDL_TG_pct | 93722 | 0.141 | 0.009 | <0.001 |  | 3797 | 0.093 | 0.067 | 0.164 |  | 92 | 0.102 (0.057, 0.148) | <0.001 |
| M_VLDL_TG | 93722 | 0.153 | 0.010 | <0.001 |  | 3797 | 0.166 | 0.080 | 0.037 |  | 93 | 0.059 (0.015, 0.103) | 0.010 |
| L_VLDL_TG | 93722 | 0.146 | 0.010 | <0.001 |  | 3797 | 0.153 | 0.077 | 0.047 |  | 94 | 0.054 (0.007, 0.1) | 0.023 |
| XL_VLDL_TG | 93722 | 0.163 | 0.010 | <0.001 |  | 3797 | 0.174 | 0.077 | 0.023 |  | 94 | 0.074 (0.026, 0.121) | 0.002 |
| XL_VLDL_TG_pct | 93090 | 0.136 | 0.011 | <0.001 |  | 3776 | 0.091 | 0.080 | 0.251 |  | 93 | 0.068 (0.023, 0.114) | 0.004 |
| XXL_VLDL_TG | 93722 | 0.087 | 0.010 | <0.001 |  | 3797 | 0.088 | 0.076 | 0.246 |  | 94 | 0.062 (0.017, 0.107) | 0.007 |
| LDL_TG | 93722 | 0.238 | 0.011 | <0.001 |  | 3797 | 0.276 | 0.084 | 0.001 |  | 92 | 0.088 (0.046, 0.13) | <0.001 |
| S_LDL_TG | 93722 | 0.213 | 0.010 | <0.001 |  | 3797 | 0.245 | 0.081 | 0.003 |  | 92 | 0.082 (0.039, 0.125) | <0.001 |
| S_LDL_TG_pct | 93722 | 0.205 | 0.010 | <0.001 |  | 3797 | 0.199 | 0.076 | 0.009 |  | 91 | 0.099 (0.055, 0.144) | <0.001 |
| M_LDL_TG | 93722 | 0.230 | 0.011 | <0.001 |  | 3797 | 0.273 | 0.084 | 0.001 |  | 92 | 0.085 (0.042, 0.127) | <0.001 |
| M_LDL_TG_pct | 93722 | 0.228 | 0.010 | <0.001 |  | 3797 | 0.195 | 0.075 | 0.010 |  | 91 | 0.106 (0.062, 0.149) | <0.001 |
| L_LDL_TG | 93722 | 0.230 | 0.011 | <0.001 |  | 3797 | 0.254 | 0.086 | 0.003 |  | 92 | 0.091 (0.049, 0.133) | <0.001 |
| L_LDL_TG_pct | 93722 | 0.259 | 0.010 | <0.001 |  | 3797 | 0.221 | 0.073 | 0.003 |  | 91 | 0.132 (0.087, 0.178) | <0.001 |
| IDL_TG_pct | 93722 | 0.244 | 0.010 | <0.001 |  | 3797 | 0.225 | 0.071 | 0.001 |  | 91 | 0.129 (0.084, 0.175) | <0.001 |
| IDL_TG | 93722 | 0.240 | 0.011 | <0.001 |  | 3797 | 0.258 | 0.084 | 0.002 |  | 92 | 0.087 (0.046, 0.129) | <0.001 |
| HDL_TG | 93722 | 0.167 | 0.011 | <0.001 |  | 3797 | 0.188 | 0.084 | 0.026 |  | 92 | 0.094 (0.053, 0.135) | <0.001 |
| S_HDL_TG | 93722 | 0.215 | 0.010 | <0.001 |  | 3797 | 0.241 | 0.081 | 0.003 |  | 92 | 0.131 (0.086, 0.175) | <0.001 |
| S_HDL_TG_pct | 93722 | 0.250 | 0.010 | <0.001 |  | 3797 | 0.244 | 0.080 | 0.002 |  | 92 | 0.091 (0.045, 0.136) | <0.001 |
| M_HDL_TG | 93722 | 0.156 | 0.011 | <0.001 |  | 3797 | 0.173 | 0.084 | 0.039 |  | 93 | 0.112 (0.07, 0.154) | <0.001 |
| M_HDL_TG_pct | 93722 | 0.232 | 0.010 | <0.001 |  | 3797 | 0.221 | 0.081 | 0.007 |  | 92 | 0.09 (0.046, 0.134) | <0.001 |
| L_HDL_TG | 93722 | 0.066 | 0.011 | <0.001 |  | 3797 | 0.065 | 0.080 | 0.417 |  | 93 | 0.022 (-0.016, 0.061) | 0.260 |
| L_HDL_TG_pct | 93722 | 0.195 | 0.010 | <0.001 |  | 3797 | 0.251 | 0.077 | 0.001 |  | 92 | 0.093 (0.05, 0.137) | <0.001 |
| XL_HDL_TG_pct | 93715 | 0.172 | 0.009 | <0.001 |  | 3797 | 0.233 | 0.073 | 0.001 |  | 92 | 0.11 (0.063, 0.157) | <0.001 |
| Triglycerides in Very Large HDL | 93722 | 0.142 | 0.011 | <0.001 |  | 3797 | 0.138 | 0.080 | 0.085 |  | 94 | 0.007 (-0.034, 0.048) | 0.746 |
| TG | 93722 | 0.194 | 0.010 | <0.001 |  | 3797 | 0.206 | 0.079 | 0.009 |  | 93 | 0.081 (0.035, 0.126) | 0.001 |
| TG/PG | 93659 | 0.219 | 0.010 | <0.001 |  | 3796 | 0.205 | 0.074 | 0.006 |  | 94 | 0.075 (0.025, 0.124) | 0.003 |
| **Cholesterol** |  |  |  |  |  |  |  |  |  |  |  |  |  |
| VLDL Cholesterol | 93722 | 0.108 | 0.010 | <0.001 |  | 3797 | 0.149 | 0.077 | 0.053 |  | 93 | 0.013 (-0.028, 0.055) | 0.531 |
| Cholesterol in Very Small VLDL | 93722 | 0.037 | 0.010 | <0.001 |  | 3797 | 0.056 | 0.069 | 0.411 |  | 93 | -0.032 (-0.073, 0.01) | 0.139 |
| Cholesterol in Small VLDL | 93722 | 0.101 | 0.010 | <0.001 |  | 3797 | 0.144 | 0.079 | 0.069 |  | 93 | 0.011 (-0.03, 0.052) | 0.597 |
| L_VLDL_C | 93722 | 0.154 | 0.010 | <0.001 |  | 3797 | 0.183 | 0.078 | 0.019 |  | 93 | 0.051 (0.007, 0.095) | 0.024 |
| XL_VLDL_C | 93722 | 0.150 | 0.010 | <0.001 |  | 3797 | 0.179 | 0.076 | 0.018 |  | 94 | 0.049 (0.003, 0.094) | 0.037 |
| XXL_VLDL_C | 93722 | 0.169 | 0.010 | <0.001 |  | 3797 | 0.168 | 0.078 | 0.032 |  | 93 | 0.078 (0.033, 0.122) | 0.001 |
| Remnant Cholesterol (Non-HDL, Non-LDL -Cholesterol) | 93722 | 0.036 | 0.010 | <0.001 |  | 3797 | 0.077 | 0.072 | 0.281 |  | 93 | -0.022 (-0.062, 0.019) | 0.293 |
| **Free Cholesterol** |  |  |  |  |  |  |  |  |  |  |  |  |  |
| VLDL_FC | 93722 | 0.135 | 0.010 | <0.001 |  | 3797 | 0.172 | 0.078 | 0.027 |  | 93 | 0.031 (-0.012, 0.075) | 0.161 |
| XS_VLDL_FC | 93722 | 0.093 | 0.010 | <0.001 |  | 3797 | 0.105 | 0.074 | 0.157 |  | 93 | -0.004 (-0.046, 0.037) | 0.840 |
| S_VLDL_FC | 93722 | 0.065 | 0.010 | <0.001 |  | 3797 | 0.108 | 0.077 | 0.158 |  | 93 | -0.008 (-0.048, 0.033) | 0.711 |
| M_VLDL_FC | 93722 | 0.061 | 0.010 | <0.001 |  | 3797 | 0.106 | 0.074 | 0.152 |  | 93 | -0.012 (-0.053, 0.028) | 0.548 |
| L_VLDL_FC_pct | 93719 | 0.096 | 0.011 | <0.001 |  | 3797 | 0.162 | 0.081 | 0.046 |  | 94 | 0.039 (0.004, 0.075) | 0.031 |
| L_VLDL_FC | 93722 | 0.168 | 0.010 | <0.001 |  | 3797 | 0.191 | 0.077 | 0.014 |  | 94 | 0.06 (0.014, 0.107) | 0.011 |
| XL_VLDL_FC | 93722 | 0.165 | 0.010 | <0.001 |  | 3797 | 0.178 | 0.076 | 0.019 |  | 94 | 0.057 (0.011, 0.104) | 0.016 |
| XXL_VLDL_FC | 93722 | 0.171 | 0.010 | <0.001 |  | 3797 | 0.165 | 0.079 | 0.038 |  | 93 | 0.079 (0.034, 0.123) | 0.001 |
| XL_HDL_FC_pct | 93715 | 0.099 | 0.009 | <0.001 |  | 3797 | 0.194 | 0.064 | 0.002 |  | 91 | 0.078 (0.034, 0.122) | 0.001 |
| **Esterified cholesterol** |  |  |  |  |  |  |  |  |  |  |  |  |  |
| VLDL_CE | 93722 | 0.086 | 0.010 | <0.001 |  | 3797 | 0.127 | 0.075 | 0.092 |  | 93 | -0.001 (-0.041, 0.04) | 0.979 |
| S_VLDL_CE | 93722 | 0.120 | 0.010 | <0.001 |  | 3797 | 0.163 | 0.080 | 0.043 |  | 93 | 0.022 (-0.02, 0.063) | 0.307 |
| L_VLDL_CE | 93722 | 0.136 | 0.010 | <0.001 |  | 3797 | 0.168 | 0.078 | 0.031 |  | 94 | 0.029 (-0.015, 0.074) | 0.191 |
| XL_VLDL_CE | 93722 | 0.130 | 0.010 | <0.001 |  | 3797 | 0.168 | 0.076 | 0.027 |  | 94 | 0.038 (-0.006, 0.082) | 0.093 |
| XXL_VLDL_CE | 93722 | 0.163 | 0.010 | <0.001 |  | 3797 | 0.166 | 0.078 | 0.032 |  | 93 | 0.077 (0.032, 0.122) | 0.001 |
| S_LDL_CE | 93722 | 0.057 | 0.011 | <0.001 |  | 3797 | 0.105 | 0.082 | 0.202 |  | 93 | -0.006 (-0.047, 0.035) | 0.766 |
| M_LDL_CE | 93722 | 0.048 | 0.011 | <0.001 |  | 3797 | 0.095 | 0.083 | 0.252 |  | 93 | 0.008 (-0.033, 0.05) | 0.702 |
| M_LDL_CE_pct | 93722 | 0.055 | 0.011 | <0.001 |  | 3797 | 0.084 | 0.080 | 0.296 |  | 93 | 0.051 (0.011, 0.09) | 0.013 |
| **Apolipoproteins** |  |  |  |  |  |  |  |  |  |  |  |  |  |
| Apolipoprotein B | 93722 | 0.049 | 0.010 | <0.001 |  | 3797 | 0.093 | 0.075 | 0.218 |  | 93 | -0.024 (-0.064, 0.017) | 0.252 |
| ApoB to ApoA1 | 93722 | 0.122 | 0.010 | <0.001 |  | 3797 | 0.142 | 0.078 | 0.069 |  | 93 | -0.031 (-0.074, 0.012) | 0.164 |
| **Energy metabolism:glycolysis & ketone bodies** |  |  |  |  |  |  |  |  |  |  |  |  |  |
| Lactate | 93499 | 0.141 | 0.011 | <0.001 |  | 3789 | 0.110 | 0.084 | 0.187 |  | 92 | -0.013 (-0.057, 0.031) | 0.569 |
| Pyruvate | 93451 | 0.054 | 0.011 | <0.001 |  | 3794 | 0.119 | 0.083 | 0.155 |  | 92 | -0.007 (-0.05, 0.036) | 0.757 |
| Acetone | 93719 | 0.052 | 0.011 | <0.001 |  | 3797 | 0.114 | 0.085 | 0.180 |  | 94 | -0.009 (-0.045, 0.027) | 0.631 |
| 3-Hydroxybutyrate | 92591 | 0.076 | 0.012 | <0.001 |  | 3770 | 0.177 | 0.088 | 0.046 |  | 94 | 0.015 (-0.03, 0.061) | 0.514 |
| Acetoacetate | 93719 | 0.044 | 0.012 | <0.001 |  | 3797 | 0.194 | 0.092 | 0.035 |  | 93 | 0.023 (-0.017, 0.064) | 0.266 |

MR: Mendelian randomization; SE: standard error; SNP: single nucleotide polymorphism; CI: confidence interval; MUFA: monounsaturated fatty acids; FA: total fatty acids; SFA: saturated fatty acids; FAw6: omega−6 fatty acids; FAw3: omega−3 fatty acids; VLDL: very-low-density lipoprotein; LDL: low-density lipoprotein; HDL: high-density lipoprotein; IDL: intermediate density lipoprotein; XS_: very small; S_: small; M_: medium; L_: large; XL_: very large; XXL_: extremely large; _P: particle concentrations of lipoproteins; _PL: phospholipids in lipoproteins; TG: triglycerides; _C: total cholesterol; _CE: esterified cholesterol; _FC: free cholesterol; _pct: percentage of certain lipids to total lipids in lipoproteins.

^a^ Linear regression models were fitted for the analyses of baseline and repeat assessment data, with never smoking as the reference group, with adjustment for age, sex, fasting hours, assessment center, Townsend Deprivation Index, education, ethnicity, body mass index, waist-to-hip ratio, physical activity, and consumption of alcohol, coffee, tea, whole grains, refined grains, fruit, vegetables, unprocessed meat, processed meat, fish, milk, and sugar-sweetened beverages. Metabolites in red were positively associated with current smoking at the repeat assessment analysis.

^b^ MR analyses were performed using 94 SNPs for smoking as the instrumental variables. Estimates from the inverse-variance weighted method were reported if no outlier was detected, otherwise estimates from the MRPRESSO estimator were reported, removing outliers from the calculation. Metabolites highlighted in green were positively associated with smoking **in the MR analyses.**

**eTable 4. Metabolites inversely associated with current smoking at baseline**

| **Metabolites** | **Baseline analysis ^a^** | | | |  | **Repeat assessment ^a^** | | | |  | **MR analysis ^b^** | | |
| --- | --- | --- | --- | --- | --- | --- | --- | --- | --- | --- | --- | --- | --- |
|  | **Sample size** | **β** | **SE** | ***p*** |  | **Sample size** | **β** | **SE** | ***p*** |  | **No. of SNPs** | **β (95% CI)** | ***p*** |
| **Amino acids** |  |  |  |  |  |  |  |  |  |  |  |  |  |
| Alanine | 93718 | -0.101 | 0.011 | <0.001 |  | 3797 | -0.043 | 0.085 | 0.617 |  | 92 | -0.018 (-0.056, 0.02) | 0.344 |
| Tyrosine | 93586 | -0.069 | 0.011 | <0.001 |  | 3791 | -0.018 | 0.084 | 0.829 |  | 91 | 0.055 (0.01, 0.1) | 0.020 |
| Total Concentration of BCAA | 93696 | -0.051 | 0.011 | <0.001 |  | 3797 | 0.082 | 0.082 | 0.322 |  | 91 | 0.007 (-0.031, 0.045) | 0.712 |
| Leucine | 93718 | -0.044 | 0.011 | <0.001 |  | 3797 | 0.087 | 0.082 | 0.286 |  | 91 | 0.013 (-0.025, 0.051) | 0.512 |
| Histidine | 93577 | -0.078 | 0.012 | <0.001 |  | 3789 | -0.018 | 0.085 | 0.829 |  | 94 | -0.048 (-0.085, -0.012) | 0.009 |
| Valine | 93697 | -0.060 | 0.011 | <0.001 |  | 3797 | 0.094 | 0.083 | 0.258 |  | 92 | 0.003 (-0.038, 0.043) | 0.901 |
| Phenylalanine | 93692 | -0.061 | 0.011 | <0.001 |  | 3795 | 0.055 | 0.085 | 0.515 |  | 92 | 0.02 (-0.018, 0.059) | 0.297 |
| **Fatty acids** |  |  |  |  |  |  |  |  |  |  |  |  |  |
| PUFA | 93659 | -0.048 | 0.011 | <0.001 |  | 3796 | 0.032 | 0.078 | 0.683 |  | 92 | -0.014 (-0.054, 0.026) | 0.499 |
| FAw3/FA | 93659 | -0.270 | 0.010 | <0.001 |  | 3796 | -0.158 | 0.078 | 0.043 |  | 86 | -0.06 (-0.102, -0.018) | 0.006 |
| DHA | 93659 | -0.241 | 0.010 | <0.001 |  | 3796 | -0.189 | 0.075 | 0.012 |  | 90 | -0.06 (-0.107, -0.014) | 0.013 |
| PUFA/MUFA | 93659 | -0.343 | 0.009 | <0.001 |  | 3796 | -0.331 | 0.072 | <0.001 | | 92 | -0.157 (-0.207, -0.108) | <0.001 |
| LA/FA | 93659 | -0.193 | 0.010 | <0.001 |  | 3796 | -0.233 | 0.071 | 0.001 |  | 92 | -0.153 (-0.198, -0.107) | <0.001 |
| FAw6/FA | 93659 | -0.237 | 0.010 | <0.001 |  | 3796 | -0.279 | 0.074 | 0.000 |  | 92 | -0.141 (-0.186, -0.096) | <0.001 |
| FAw3 | 93659 | -0.174 | 0.011 | <0.001 |  | 3796 | -0.054 | 0.080 | 0.500 |  | 91 | -0.021 (-0.067, 0.025) | 0.367 |
| DHA/FA | 93659 | -0.307 | 0.010 | <0.001 |  | 3796 | -0.293 | 0.074 | <0.001 | | 90 | -0.09 (-0.138, -0.041) | <0.001 |
| PUFA/FA | 93659 | -0.324 | 0.010 | <0.001 |  | 3796 | -0.318 | 0.073 | <0.001 | | 94 | -0.152 (-0.203, -0.101) | <0.001 |
| Degree of Unsaturation | 93659 | -0.359 | 0.010 | <0.001 |  | 3796 | -0.272 | 0.073 | <0.001 | | 89 | -0.109 (-0.158, -0.059) | <0.001 |
| **Concentrations of lipoproteins** |  |  |  |  |  |  |  |  |  |  |  |  |  |
| HDL_P | 93722 | -0.141 | 0.010 | <0.001 |  | 3797 | -0.079 | 0.074 | 0.287 |  | 91 | 0.003 (-0.042, 0.049) | 0.882 |
| S_HDL_P | 93722 | -0.056 | 0.011 | <0.001 |  | 3797 | 0.030 | 0.082 | 0.718 |  | 92 | 0.077 (0.032, 0.121) | 0.001 |
| M_HDL_P | 93722 | -0.135 | 0.010 | <0.001 |  | 3797 | -0.088 | 0.075 | 0.238 |  | 92 | 0.016 (-0.031, 0.062) | 0.509 |
| L_HDL_P | 93722 | -0.138 | 0.009 | <0.001 |  | 3797 | -0.167 | 0.064 | 0.009 |  | 91 | -0.083 (-0.126, -0.04) | <0.001 |
| XL_HDL_P | 93722 | -0.101 | 0.010 | <0.001 |  | 3797 | -0.119 | 0.072 | 0.099 |  | 90 | -0.122 (-0.162, -0.083) | <0.001 |
| All lipoprotein particles | 93722 | -0.123 | 0.010 | <0.001 |  | 3797 | -0.059 | 0.074 | 0.424 |  | 91 | 0 (-0.045, 0.045) | 0.999 |
| **Diameters of lipoproteins** |  |  |  |  |  |  |  |  |  |  |  |  |  |
| HDL_D | 93722 | -0.136 | 0.009 | <0.001 |  | 3797 | -0.160 | 0.071 | 0.025 |  | 91 | -0.089 (-0.13, -0.047) | <0.001 |
| **Total Lipids** |  |  |  |  |  |  |  |  |  |  |  |  |  |
| HDL_L | 93722 | -0.138 | 0.009 | <0.001 |  | 3797 | -0.113 | 0.071 | 0.112 |  | 91 | -0.019 (-0.063, 0.025) | 0.397 |
| M_HDL_L | 93722 | -0.125 | 0.010 | <0.001 |  | 3797 | -0.082 | 0.074 | 0.268 |  | 91 | 0.026 (-0.02, 0.071) | 0.270 |
| L_HDL_L | 93722 | -0.143 | 0.009 | <0.001 |  | 3797 | -0.188 | 0.067 | 0.005 |  | 91 | -0.075 (-0.117, -0.032) | 0.001 |
| XL_HDL_L | 93722 | -0.080 | 0.009 | <0.001 |  | 3797 | -0.140 | 0.064 | 0.029 |  | 90 | -0.121 (-0.161, -0.081) | <0.001 |
| **Phospholipids** |  |  |  |  |  |  |  |  |  |  |  |  |  |
| S_VLDL_PL_pct | 93722 | -0.180 | 0.010 | <0.001 |  | 3797 | -0.127 | 0.072 | 0.080 |  | 89 | -0.101 (-0.141, -0.062) | <0.001 |
| M_VLDL_PL_pct | 93722 | -0.068 | 0.009 | <0.001 |  | 3797 | -0.010 | 0.067 | 0.881 |  | 93 | -0.069 (-0.114, -0.025) | 0.003 |
| L_LDL_PL_pct | 93722 | -0.062 | 0.011 | <0.001 |  | 3797 | -0.131 | 0.081 | 0.105 |  | 94 | 0.016 (-0.02, 0.052) | 0.388 |
| HDL_PL | 93722 | -0.117 | 0.010 | <0.001 |  | 3797 | -0.092 | 0.072 | 0.199 |  | 91 | -0.003 (-0.046, 0.04) | 0.896 |
| M_HDL_PL | 93722 | -0.088 | 0.010 | <0.001 |  | 3797 | -0.040 | 0.076 | 0.600 |  | 91 | 0.039 (-0.005, 0.084) | 0.087 |
| L_HDL_PL | 93722 | -0.135 | 0.009 | <0.001 |  | 3797 | -0.170 | 0.071 | 0.017 |  | 91 | -0.061 (-0.104, -0.019) | 0.006 |
| XL_HDL_PL | 93722 | -0.107 | 0.010 | <0.001 |  | 3797 | -0.136 | 0.073 | 0.063 |  | 90 | -0.115 (-0.155, -0.075) | 0.000 |
| **Triglycerides** |  |  |  |  |  |  |  |  |  |  |  |  |  |
| L_VLDL_TG_pct | 93719 | -0.082 | 0.011 | <0.001 |  | 3797 | -0.124 | 0.084 | 0.139 |  | 94 | -0.007 (-0.043, 0.029) | 0.696 |
| **Cholesterol** |  |  |  |  |  |  |  |  |  |  |  |  |  |
| XS_VLDL_C_pct | 93722 | -0.209 | 0.009 | <0.001 |  | 3797 | -0.170 | 0.065 | 0.008 |  | 90 | -0.125 (-0.171, -0.079) | <0.001 |
| S_VLDL_C_pct | 93722 | -0.128 | 0.010 | <0.001 |  | 3797 | -0.068 | 0.074 | 0.355 |  | 93 | -0.073 (-0.119, -0.027) | 0.003 |
| M_VLDL_C_pct | 93722 | -0.158 | 0.009 | <0.001 |  | 3797 | -0.115 | 0.067 | 0.089 |  | 92 | -0.108 (-0.154, -0.062) | <0.001 |
| L_VLDL_C_pct | 93719 | -0.053 | 0.011 | <0.001 |  | 3797 | 0.012 | 0.080 | 0.877 |  | 94 | -0.045 (-0.089, -0.002) | 0.040 |
| XL_VLDL_C_pct | 93090 | -0.170 | 0.010 | <0.001 |  | 3776 | -0.119 | 0.075 | 0.112 |  | 94 | -0.084 (-0.132, -0.036) | 0.001 |
| S_LDL_C_pct | 93722 | -0.201 | 0.010 | <0.001 |  | 3797 | -0.085 | 0.074 | 0.254 |  | 94 | -0.035 (-0.077, 0.008) | 0.109 |
| M_LDL_C_pct | 93722 | -0.173 | 0.009 | <0.001 |  | 3797 | -0.122 | 0.069 | 0.078 |  | 92 | -0.072 (-0.115, -0.03) | 0.001 |
| L_LDL_C_pct | 93722 | -0.186 | 0.009 | <0.001 |  | 3797 | -0.131 | 0.064 | 0.040 |  | 92 | -0.139 (-0.184, -0.094) | <0.001 |
| IDL_C | 93722 | -0.051 | 0.009 | <0.001 |  | 3797 | -0.023 | 0.066 | 0.725 |  | 91 | -0.041 (-0.081, -0.002) | 0.044 |
| IDL_C_pct | 93722 | -0.205 | 0.010 | <0.001 |  | 3797 | -0.209 | 0.068 | 0.002 |  | 90 | -0.104 (-0.145, -0.062) | 0.000 |
| HDL_C | 93722 | -0.182 | 0.009 | <0.001 |  | 3797 | -0.165 | 0.069 | 0.017 |  | 91 | -0.047 (-0.092, -0.002) | 0.044 |
| S_HDL_C | 93722 | -0.083 | 0.011 | <0.001 |  | 3797 | 0.004 | 0.082 | 0.964 |  | 91 | 0.062 (0.019, 0.106) | 0.006 |
| S_HDL_C_pct | 93722 | -0.204 | 0.011 | <0.001 |  | 3797 | -0.205 | 0.082 | 0.012 |  | 92 | -0.098 (-0.141, -0.055) | <0.001 |
| M_HDL_C | 93722 | -0.160 | 0.010 | <0.001 |  | 3797 | -0.117 | 0.075 | 0.119 |  | 93 | 0 (-0.048, 0.048) | 0.989 |
| M_HDL_C_pct | 93722 | -0.253 | 0.010 | <0.001 |  | 3797 | -0.267 | 0.076 | <0.001 | | 92 | -0.1 (-0.146, -0.054) | <0.001 |
| L_HDL_C_pct | 93722 | -0.163 | 0.009 | <0.001 |  | 3797 | -0.249 | 0.064 | <0.001 | | 92 | -0.149 (-0.193, -0.104) | <0.001 |
| L_HDL_C | 93722 | -0.150 | 0.009 | <0.001 |  | 3797 | -0.193 | 0.066 | 0.004 |  | 91 | -0.091 (-0.134, -0.047) | <0.001 |
| XL_HDL_C_pct | 93715 | -0.057 | 0.010 | <0.001 |  | 3797 | 0.051 | 0.068 | 0.458 |  | 90 | 0.021 (-0.016, 0.058) | 0.267 |
| XL_HDL_C | 93722 | -0.100 | 0.009 | <0.001 |  | 3797 | -0.147 | 0.064 | 0.020 |  | 91 | -0.134 (-0.177, -0.092) | <0.001 |
| Total Cholesterol | 93722 | -0.046 | 0.010 | <0.001 |  | 3797 | 0.009 | 0.070 | 0.901 |  | 93 | -0.038 (-0.08, 0.005) | 0.085 |
| **Free Cholesterol** |  |  |  |  |  |  |  |  |  |  |  |  |  |
| XS_VLDL_FC_pct | 93722 | -0.132 | 0.010 | <0.001 |  | 3797 | -0.114 | 0.072 | 0.114 |  | 91 | -0.059 (-0.102, -0.016) | 0.009 |
| S_VLDL_FC_pct | 93722 | -0.186 | 0.010 | <0.001 |  | 3797 | -0.139 | 0.071 | 0.051 |  | 90 | -0.095 (-0.136, -0.053) | <0.001 |
| M_VLDL_FC_pct | 93722 | -0.124 | 0.009 | <0.001 |  | 3797 | -0.071 | 0.067 | 0.288 |  | 93 | -0.091 (-0.137, -0.045) | <0.001 |
| XL_VLDL_FC_pct | 93090 | -0.119 | 0.011 | <0.001 |  | 3776 | -0.099 | 0.080 | 0.216 |  | 93 | -0.079 (-0.123, -0.035) | 0.001 |
| LDL_FC | 93722 | -0.056 | 0.011 | <0.001 |  | 3797 | -0.010 | 0.079 | 0.900 |  | 91 | -0.035 (-0.075, 0.004) | 0.081 |
| S_LDL_FC_pct | 93722 | -0.199 | 0.010 | <0.001 |  | 3797 | -0.214 | 0.075 | 0.004 |  | 93 | -0.107 (-0.153, -0.061) | <0.001 |
| M_LDL_FC | 93722 | -0.036 | 0.011 | 0.001 |  | 3797 | 0.013 | 0.082 | 0.875 |  | 92 | -0.028 (-0.069, 0.012) | 0.174 |
| M_LDL_FC_pct | 93722 | -0.214 | 0.010 | <0.001 |  | 3797 | -0.203 | 0.072 | 0.005 |  | 93 | -0.124 (-0.172, -0.077) | <0.001 |
| L_LDL_FC_pct | 93722 | -0.205 | 0.009 | <0.001 |  | 3797 | -0.193 | 0.066 | 0.004 |  | 90 | -0.134 (-0.18, -0.087) | <0.001 |
| L_LDL_FC | 93722 | -0.067 | 0.011 | <0.001 |  | 3797 | -0.018 | 0.077 | 0.815 |  | 92 | -0.05 (-0.091, -0.009) | 0.020 |
| IDL_FC | 93722 | -0.063 | 0.009 | <0.001 |  | 3797 | -0.015 | 0.066 | 0.820 |  | 92 | -0.054 (-0.096, -0.012) | 0.014 |
| IDL_FC_pct | 93722 | -0.203 | 0.010 | <0.001 |  | 3797 | -0.110 | 0.075 | 0.141 |  | 93 | -0.089 (-0.134, -0.044) | <0.001 |
| HDL_FC | 93722 | -0.129 | 0.009 | <0.001 |  | 3797 | -0.115 | 0.068 | 0.093 |  | 91 | -0.044 (-0.086, -0.001) | 0.048 |
| S_HDL_FC_pct | 93722 | -0.034 | 0.010 | 0.001 |  | 3797 | -0.067 | 0.075 | 0.371 |  | 93 | -0.107 (-0.148, -0.065) | <0.001 |
| M_HDL_FC_pct | 93722 | -0.151 | 0.009 | <0.001 |  | 3797 | -0.156 | 0.068 | 0.021 |  | 91 | -0.082 (-0.124, -0.041) | <0.001 |
| M_HDL_FC | 93722 | -0.130 | 0.010 | <0.001 |  | 3797 | -0.093 | 0.074 | 0.207 |  | 91 | -0.004 (-0.048, 0.041) | 0.868 |
| L_HDL_FC | 93722 | -0.108 | 0.009 | <0.001 |  | 3797 | -0.159 | 0.065 | 0.015 |  | 91 | -0.083 (-0.126, -0.041) | <0.001 |
| XL_HDL_FC | 93722 | -0.055 | 0.009 | <0.001 |  | 3797 | -0.090 | 0.067 | 0.180 |  | 91 | -0.139 (-0.181, -0.097) | <0.001 |
| **Esterified cholesterol** |  |  |  |  |  |  |  |  |  |  |  |  |  |
| XS_VLDL_CE_pct | 93722 | -0.199 | 0.009 | <0.001 |  | 3797 | -0.164 | 0.061 | 0.007 |  | 90 | -0.126 (-0.172, -0.08) | <0.001 |
| S_VLDL_CE_pct | 93722 | -0.067 | 0.011 | <0.001 |  | 3797 | -0.005 | 0.078 | 0.944 |  | 94 | -0.049 (-0.095, -0.003) | 0.035 |
| M_VLDL_CE_pct | 93722 | -0.168 | 0.009 | <0.001 |  | 3797 | -0.129 | 0.068 | 0.058 |  | 92 | -0.11 (-0.157, -0.064) | <0.001 |
| L_VLDL_CE_pct | 93719 | -0.100 | 0.010 | <0.001 |  | 3797 | -0.045 | 0.073 | 0.536 |  | 93 | -0.068 (-0.112, -0.023) | 0.004 |
| XL_VLDL_CE_pct | 93090 | -0.178 | 0.010 | <0.001 |  | 3776 | -0.131 | 0.074 | 0.075 |  | 94 | -0.081 (-0.128, -0.033) | 0.001 |
| Large LDL_CE_pct | 93722 | -0.063 | 0.010 | <0.001 |  | 3797 | 0.018 | 0.077 | 0.816 |  | 94 | -0.046 (-0.09, -0.003) | 0.038 |
| IDL_CE | 93722 | -0.047 | 0.009 | <0.001 |  | 3797 | -0.026 | 0.065 | 0.695 |  | 92 | -0.048 (-0.088, -0.007) | 0.023 |
| IDL_CE_pct | 93722 | -0.161 | 0.010 | <0.001 |  | 3797 | -0.201 | 0.070 | 0.004 |  | 92 | -0.084 (-0.126, -0.042) | <0.001 |
| HDL_CE | 93722 | -0.196 | 0.009 | <0.001 |  | 3797 | -0.179 | 0.070 | 0.010 |  | 92 | -0.053 (-0.1, -0.006) | 0.030 |
| S_HDL_CE | 93722 | -0.100 | 0.011 | <0.001 |  | 3797 | -0.008 | 0.083 | 0.926 |  | 92 | 0.068 (0.024, 0.112) | 0.003 |
| S_HDL_CE_pct | 93722 | -0.194 | 0.011 | <0.001 |  | 3797 | -0.176 | 0.084 | 0.036 |  | 93 | -0.072 (-0.115, -0.03) | 0.001 |
| M_HDL_CE | 93722 | -0.167 | 0.010 | <0.001 |  | 3797 | -0.122 | 0.075 | 0.104 |  | 92 | 0.006 (-0.041, 0.053) | 0.806 |
| M_HDL_CE_pct | 93722 | -0.250 | 0.010 | <0.001 |  | 3797 | -0.267 | 0.079 | 0.001 |  | 92 | -0.094 (-0.139, -0.049) | <0.001 |
| L_HDL_CE_pct | 93722 | -0.192 | 0.009 | <0.001 |  | 3797 | -0.272 | 0.067 | <0.001 | | 91 | -0.136 (-0.18, -0.092) | <0.001 |
| L_HDL_CE | 93722 | -0.160 | 0.009 | <0.001 |  | 3797 | -0.203 | 0.066 | 0.002 |  | 91 | -0.093 (-0.137, -0.048) | <0.001 |
| XL_HDL_CE | 93722 | -0.099 | 0.008 | <0.001 |  | 3797 | -0.126 | 0.066 | 0.056 |  | 91 | -0.131 (-0.174, -0.088) | <0.001 |
| XL_HDL_CE_pct | 93715 | -0.167 | 0.010 | <0.001 |  | 3797 | -0.136 | 0.073 | 0.060 |  | 92 | -0.049 (-0.091, -0.007) | 0.026 |
| Total Esterified Cholesterol | 93722 | -0.058 | 0.010 | <0.001 |  | 3797 | -0.002 | 0.069 | 0.975 |  | 93 | -0.039 (-0.081, 0.004) | 0.079 |
| **Apolipoproteins** |  |  |  |  |  |  |  |  |  |  |  |  |  |
| ApoA1 | 93722 | -0.137 | 0.010 | <0.001 |  | 3797 | -0.093 | 0.073 | 0.201 |  | 91 | 0.001 (-0.044, 0.046) | 0.969 |
| **Cholines** |  |  |  |  |  |  |  |  |  |  |  |  |  |
| Total Cholines | 93659 | -0.035 | 0.010 | <0.001 |  | 3796 | 0.017 | 0.072 | 0.816 |  | 92 | -0.002 (-0.045, 0.041) | 0.929 |
| Phosphatidylcholines | 93659 | -0.033 | 0.010 | 0.001 |  | 3796 | 0.007 | 0.072 | 0.927 |  | 92 | 0.006 (-0.038, 0.05) | 0.785 |
| Sphingomyelins | 93659 | -0.033 | 0.010 | 0.001 |  | 3796 | 0.013 | 0.070 | 0.857 |  | 92 | -0.027 (-0.069, 0.015) | 0.214 |
| **Energy metabolism:glycolysis & ketone bodies** |  |  |  |  |  |  |  |  |  |  |  |  |  |
| Glucose | 93552 | -0.147 | 0.009 | <0.001 |  | 3790 | 0.089 | 0.067 | 0.183 |  | 92 | 0.008 (-0.033, 0.05) | 0.695 |
| Citrate | 93711 | -0.214 | 0.011 | <0.001 |  | 3796 | 0.025 | 0.083 | 0.766 |  | 92 | -0.108 (-0.149, -0.068) | 0.000 |
| Acetate | 93691 | -0.072 | 0.011 | <0.001 |  | 3795 | -0.073 | 0.083 | 0.376 |  | 92 | -0.064 (-0.101, -0.028) | 0.001 |
| **Fluid balance** |  |  |  |  |  |  |  |  |  |  |  |  |  |
| Creatinine | 89464 | -0.054 | 0.009 | <0.001 |  | 3632 | 0.004 | 0.072 | 0.956 |  | 92 | 0.001 (-0.036, 0.039) | 0.940 |
| Albumin | 93710 | -0.140 | 0.011 | <0.001 |  | 3796 | -0.090 | 0.084 | 0.282 |  | 91 | -0.045 (-0.088, -0.002) | 0.045 |

MR: Mendelian randomization; SE: standard error; SNP: single nucleotide polymorphism; CI: confidence interval; BCAA: branched-chain amino acids; PUFA: polyunsaturated fatty acids; MUFA: monounsaturated fatty acids; DHA: docosahexaenoic acid; FAw6: omega−6 fatty acids; FAw3: omega−3 fatty acids; VLDL: very-low-density lipoprotein; LDL: low-density lipoprotein; HDL: high-density lipoprotein; IDL: intermediate density lipoprotein; XS_: very small; S_: small; M_: medium; LA: linoleic acid; L_: large; XL_: very large; XXL_: extremely large; _P: particle concentrations of lipoproteins; _PL: phospholipids in lipoproteins; TG: triglycerides; _C: total cholesterol; _CE: esterified cholesterol; _FC: free cholesterol; _pct: percentage of certain lipids to total lipids in lipoproteins.

^a^ Linear regression models were fitted for the analyses of baseline and repeat assessment data, with never smoking as the reference group, with adjustment for age, sex, fasting hours, body mass index, waist-to-hip ratio, physical activity, and consumption of alcohol, coffee, tea, whole grains, refined grains, fruit, vegetables, unprocessed meat, processed meat, fish, milk, and sugar-sweetened beverages. Metabolites in red were inversely associated with current smoking at the repeat assessment analysis.

^b^ MR analyses were performed using 94 SNPs for smoking as the instrumental variables. Estimates from the inverse-variance weighted method were reported if no outlier was detected, otherwise estimates from the MRPRESSO estimator were reported, excluding outliers from the calculation. Metabolites highlighted in green were inversely associated with smoking in the MR analyses.

**eTable 5. Associations of individual metabolites increased by smoking a with type 2 diabetes**

| **Metabolites** | **Cases** | **HR (95% CI) per SD increase in metabolites** | **p** |
| --- | --- | --- | --- |
| **Inflammation** |  |  |  |
| Glycoprotein Acetyls | 1869 | 1.42 (1.35, 1.50) | <0.001 |
| **Fatty acids** |  |  |  |
| SFA | 1869 | 1.44 (1.37, 1.50) | <0.001 |
| SFA/FA | 1869 | 1.45 (1.39, 1.52) | <0.001 |
| Total Fatty Acids | 1869 | 1.36 (1.30, 1.43) | <0.001 |
| MUFA | 1869 | 1.48 (1.41, 1.55) | <0.001 |
| MUFA/FA | 1869 | 1.64 (1.56, 1.74) | <0.001 |
| **Concentrations of lipoproteins** |  |  |  |
| S_VLDL_P | 1869 | 1.43 (1.35, 1.51) | <0.001 |
| L_VLDL_P | 1869 | 1.64 (1.54, 1.75) | <0.001 |
| XL_VLDL_P | 1869 | 1.81 (1.69, 1.95) | <0.001 |
| XXL_VLDL_P | 1869 | 1.85 (1.71, 2.01) | <0.001 |
| **Sizes of lipoproteins** |  |  |  |
| VLDL_D | 1869 | 1.57 (1.48, 1.66) | <0.001 |
| **Total Lipids** |  |  |  |
| VLDL_L | 1869 | 1.54 (1.46, 1.63) | <0.001 |
| S_VLDL_L | 1869 | 1.42 (1.35, 1.50) | <0.001 |
| L_VLDL_L | 1869 | 1.62 (1.52, 1.72) | <0.001 |
| XL_VLDL_L | 1869 | 1.78 (1.66, 1.91) | <0.001 |
| XXL_VLDL_L | 1869 | 1.76 (1.64, 1.89) | <0.001 |
| **Phospholipids** |  |  |  |
| VLDL_PL | 1869 | 1.49 (1.41, 1.58) | <0.001 |
| XS_VLDL_PL_pct | 1869 | 1.38 (1.31, 1.46) | <0.001 |
| L_VLDL_PL | 1869 | 2.15 (1.93, 2.38) | <0.001 |
| L_VLDL_PL_pct | 1869 | 1.75 (1.60, 1.92) | <0.001 |
| XL_VLDL_PL | 1869 | 2.07 (1.88, 2.29) | <0.001 |
| XXL_VLDL_PL | 1869 | 2.01 (1.82, 2.23) | <0.001 |
| XXL_VLDL_PL_pct | 1869 | 1.13 (1.06, 1.21) | <0.001 |
| S_HDL_PL_pct | 1869 | 1.11 (1.06, 1.17) | <0.001 |
| M_HDL_PL_pct | 1869 | 1.55 (1.48, 1.62) | <0.001 |
| L_HDL_PL_pct | 1869 | 1.50 (1.43, 1.57) | <0.001 |
| **Triglycerides** |  |  |  |
| VLDL_TG | 1869 | 1.62 (1.53, 1.71) | <0.001 |
| XS_VLDL_TG | 1869 | 1.53 (1.46, 1.60) | <0.001 |
| XS_VLDL_TG_pct | 1869 | 1.61 (1.53, 1.69) | <0.001 |
| S_VLDL_TG_pct | 1869 | 1.46 (1.39, 1.54) | <0.001 |
| S_VLDL_TG | 1869 | 1.56 (1.48, 1.64) | <0.001 |
| M_VLDL_TG_pct | 1869 | 1.50 (1.43, 1.58) | <0.001 |
| M_VLDL_TG | 1869 | 1.50 (1.42, 1.59) | <0.001 |
| L_VLDL_TG | 1869 | 1.59 (1.50, 1.69) | <0.001 |
| XL_VLDL_TG | 1869 | 1.89 (1.76, 2.04) | <0.001 |
| XL_VLDL_TG_pct | 1869 | 1.55 (1.46, 1.64) | <0.001 |
| XXL_VLDL_TG | 1869 | 2.01 (1.81, 2.22) | <0.001 |
| LDL_TG | 1869 | 1.51 (1.45, 1.58) | <0.001 |
| S_LDL_TG_pct | 1869 | 1.51 (1.44, 1.58) | <0.001 |
| S_LDL_TG | 1869 | 1.55 (1.48, 1.62) | <0.001 |
| M_LDL_TG | 1869 | 1.53 (1.46, 1.60) | <0.001 |
| M_LDL_TG_pct | 1869 | 1.46 (1.40, 1.52) | <0.001 |
| L_LDL_TG_pct | 1869 | 1.50 (1.44, 1.57) | <0.001 |
| L_LDL_TG | 1869 | 1.40 (1.35, 1.45) | <0.001 |
| IDL_TG_pct | 1869 | 1.55 (1.48, 1.62) | <0.001 |
| IDL_TG | 1869 | 1.48 (1.42, 1.55) | <0.001 |
| HDL_TG | 1869 | 1.46 (1.39, 1.53) | <0.001 |
| S_HDL_TG_pct | 1869 | 1.73 (1.63, 1.83) | <0.001 |
| S_HDL_TG | 1869 | 1.67 (1.58, 1.76) | <0.001 |
| M_HDL_TG | 1869 | 1.45 (1.38, 1.52) | <0.001 |
| M_HDL_TG_pct | 1869 | 1.64 (1.55, 1.73) | <0.001 |
| L_HDL_TG_pct | 1869 | 1.56 (1.48, 1.64) | <0.001 |
| XL_HDL_TG_pct | 1869 | 1.60 (1.52, 1.68) | <0.001 |
| TG | 1869 | 1.60 (1.52, 1.68) | <0.001 |
| TG/PG | 1869 | 1.74 (1.64, 1.85) | <0.001 |
| **Cholesterol** |  |  |  |
| L_VLDL_C | 1869 | 1.56 (1.46, 1.67) | <0.001 |
| XL_VLDL_C | 1869 | 1.64 (1.53, 1.76) | <0.001 |
| XXL_VLDL_C | 1869 | 1.71 (1.59, 1.83) | <0.001 |
| **Free Cholesterol** |  |  |  |
| L_VLDL_FC | 1869 | 1.66 (1.56, 1.78) | <0.001 |
| L_VLDL_FC_pct | 1869 | 1.04 (0.97, 1.11) | 0.280 |
| XL_VLDL_FC | 1869 | 1.74 (1.62, 1.86) | <0.001 |
| XXL_VLDL_FC | 1869 | 1.74 (1.62, 1.86) | <0.001 |
| XL_HDL_FC_pct | 1869 | 1.31 (1.25, 1.39) | <0.001 |
| **Esterified cholesterol** |  |  |  |
| XXL_VLDL_CE | 1869 | 1.69 (1.57, 1.81) | <0.001 |
| M_LDL_CE_pct | 1869 | 1.18 (1.11, 1.24) | <0.001 |

HR: hazard ratio; CI: confidence interval; MUFA: monounsaturated fatty acids; FA: total fatty acids; SFA: saturated fatty acids; FAw6: omega−6 fatty acids; FAw3: omega−3 fatty acids; VLDL: very-low-density lipoprotein; LDL: low-density lipoprotein; HDL: high-density lipoprotein; IDL: intermediate density lipoprotein; XS_: very small; S_: small; M_: medium; L_: large; XL_: very large; XXL_: extremely large; _P: particle concentrations of lipoproteins; _PL: phospholipids in lipoproteins; TG: triglycerides; _C: total cholesterol; _CE: esterified cholesterol; _FC: free cholesterol; _pct: percentage of certain lipids to total lipids in lipoproteins.

Cox models were fitted with attained age as the time scale, and with adjustment for age groups (through stratification), sex (through stratification), assessment center, education, Townsend deprivation index, body mass index, alcohol intake, physical activity, and consumption of vegetable oil, oily fish, non-oily fish, coffee, tea, fruits, vegetable, unprocessed meat, processed meat, sugar or foods/drinks containing sugar, whole grain, refined grain, and family history of diabetes.

^a^ Metabolites identified by both baseline analysis and Mendelian randomization analysis.

**eTable 6. Associations of individual metabolites decreased by smoking a with type 2 diabetes**

| **Metabolites** | **Cases** | **HR (95% CI) per SD increase in metabolites** | **p** |
| --- | --- | --- | --- |
| **Amino acids** |  |  |  |
| Histidine | 1869 | 0.95 (0.90, 0.99) | 0.019 |
| **Fatty acids** |  |  |  |
| PUFA/FA | 1869 | 0.61 (0.58, 0.64) | <0.001 |
| Degree of unsaturation | 1869 | 0.63 (0.60, 0.66) | <0.001 |
| LA/FA | 1869 | 0.66 (0.63, 0.69) | <0.001 |
| FAw3/FA | 1869 | 0.91 (0.87, 0.96) | <0.001 |
| Faw6/FA | 1869 | 0.64 (0.61, 0.67) | <0.001 |
| DHA/FA | 1869 | 0.76 (0.73, 0.79) | <0.001 |
| DHA | 1869 | 0.89 (0.85, 0.93) | <0.001 |
| PUFA/MUFA | 1869 | 0.60 (0.57, 0.63) | <0.001 |
| **Concentrations of lipoproteins** |  |  |  |
| L_HDL_P | 1869 | 0.72 (0.69, 0.76) | <0.001 |
| XL_HDL_P | 1869 | 0.76 (0.71, 0.82) | <0.001 |
| **Sizes of lipoproteins** |  |  |  |
| HDL_D | 1869 | 0.70 (0.66, 0.75) | <0.001 |
| **Total Lipids** |  |  |  |
| L_HDL_L | 1869 | 0.72 (0.68, 0.76) | <0.001 |
| XL_HDL_L | 1869 | 0.76 (0.71, 0.80) | <0.001 |
| **Phospholipids** |  |  |  |
| S_VLDL_PL_pct | 1869 | 0.66 (0.62, 0.69) | <0.001 |
| M_VLDL_PL_pct | 1869 | 0.76 (0.73, 0.80) | <0.001 |
| L_HDL_PL | 1869 | 0.86 (0.83, 0.88) | <0.001 |
| XL_HDL_PL | 1869 | 0.71 (0.66, 0.76) | <0.001 |
| **Cholesterol** |  |  |  |
| XS_VLDL_C_pct | 1869 | 0.63 (0.60, 0.66) | <0.001 |
| S_VLDL_C_pct | 1869 | 0.70 (0.67, 0.74) | <0.001 |
| M_VLDL_C_pct | 1869 | 0.65 (0.62, 0.68) | <0.001 |
| L_VLDL_C_pct | 1869 | 0.73 (0.69, 0.77) | <0.001 |
| XL_VLDL_C_pct | 1869 | 0.62 (0.59, 0.66) | <0.001 |
| M_LDL_C_pct | 1869 | 0.87 (0.85, 0.88) | <0.001 |
| L_LDL_C_pct | 1869 | 0.87 (0.85, 0.89) | <0.001 |
| IDL_C | 1869 | 0.89 (0.84, 0.94) | <0.001 |
| IDL_C_pct | 1869 | 0.71 (0.68, 0.74) | <0.001 |
| HDL_C | 1869 | 0.71 (0.67, 0.75) | <0.001 |
| S_HDL_C_pct | 1869 | 0.74 (0.72, 0.77) | <0.001 |
| M_HDL_C_pct | 1869 | 0.68 (0.65, 0.70) | <0.001 |
| L_HDL_C | 1869 | 0.77 (0.74, 0.79) | <0.001 |
| L_HDL_C_pct | 1869 | 0.68 (0.65, 0.70) | <0.001 |
| XL_HDL_C | 1869 | 0.70 (0.66, 0.74) | <0.001 |
| **Free Cholesterol** |  |  |  |
| XS_VLDL_FC_pct | 1869 | 0.76 (0.73, 0.79) | <0.001 |
| S_VLDL_FC_pct | 1869 | 0.65 (0.62, 0.69) | <0.001 |
| M_VLDL_FC_pct | 1869 | 0.70 (0.66, 0.73) | <0.001 |
| XL_VLDL_FC_pct | 1869 | 0.65 (0.62, 0.70) | <0.001 |
| S_LDL_FC_pct | 1869 | 0.74 (0.72, 0.77) | <0.001 |
| M_LDL_FC_pct | 1869 | 0.69 (0.66, 0.71) | <0.001 |
| L_LDL_FC | 911 | 0.86 (0.80, 0.93) | <0.001 |
| L_LDL_FC_pct | 911 | 0.71 (0.67, 0.75) | <0.001 |
| IDL_FC_pct | 911 | 0.78 (0.73, 0.83) | <0.001 |
| IDL_FC | 911 | 0.86 (0.80, 0.93) | <0.001 |
| HDL_FC | 911 | 0.82 (0.75, 0.90) | <0.001 |
| S_HDL_FC_pct | 911 | 1.02 (0.94, 1.10) | 0.657 |
| M_HDL_FC_pct | 911 | 0.77 (0.71, 0.84) | <0.001 |
| L_HDL_FC | 911 | 0.85 (0.81, 0.90) | <0.001 |
| XL_HDL_FC | 911 | 0.85 (0.78, 0.93) | <0.001 |
| **Esterified cholesterol** |  |  |  |
| XS_VLDL_CE_pct | 911 | 0.66 (0.62, 0.70) | <0.001 |
| S_VLDL_CE_pct | 911 | 0.80 (0.74, 0.86) | <0.001 |
| M_VLDL_CE_pct | 911 | 0.66 (0.61, 0.71) | <0.001 |
| L_VLDL_CE_pct | 911 | 0.73 (0.68, 0.78) | <0.001 |
| XL_VLDL_CE_pct | 908 | 0.64 (0.59, 0.69) | <0.001 |
| L_LDL_CE_pct | 911 | 0.91 (0.87, 0.97) | 0.001 |
| IDL_CE_pct | 911 | 0.72 (0.68, 0.77) | <0.001 |
| IDL_CE | 911 | 0.86 (0.79, 0.92) | <0.001 |
| HDL_CE | 911 | 0.68 (0.62, 0.73) | <0.001 |
| S_HDL_CE_pct | 911 | 0.71 (0.67, 0.76) | <0.001 |
| M_HDL_CE_pct | 911 | 0.69 (0.65, 0.73) | <0.001 |
| L_HDL_CE_pct | 911 | 0.68 (0.64, 0.72) | <0.001 |
| L_HDL_CE | 911 | 0.77 (0.74, 0.81) | <0.001 |
| XL_HDL_CE | 911 | 0.86 (0.83, 0.90) | <0.001 |
| XL_HDL_CE_pct | 911 | 0.76 (0.72, 0.81) | <0.001 |
| **Energy metabolism:glycolysis & ketone bodies** |  |  |  |
| Citrate | 911 | 1.01 (0.94, 1.08) | 0.780 |
| Acetate | 911 | 0.93 (0.89, 0.98) | 0.009 |
| **Fluid balance** |  |  |  |
| Albumin | 911 | 0.95 (0.89, 1.01) | 0.126 |

PUFA: polyunsaturated fatty acids; MUFA: monounsaturated fatty acids; DHA: docosahexaenoic acid; Faw6: omega−6 fatty acids; Faw3: omega−3 fatty acids; VLDL: very-low-density lipoprotein; LDL: low-density lipoprotein; HDL: high-density lipoprotein; IDL: intermediate density lipoprotein; XS_: very small; S_: small; M_: medium; L_: large; XL_: very large; XXL_: extremely large; _P: particle concentrations of lipoproteins; _PL: phospholipids in lipoproteins; TG: triglycerides; _C: total cholesterol; _CE: esterified cholesterol; _FC: free cholesterol; _pct: percentage of certain lipids to total lipids in lipoproteins.

Cox models were fitted with attained age as the time scale, and with adjustment for age groups (through stratification), sex (through stratification), assessment center, education, Townsend deprivation index, body mass index, alcohol intake, physical activity, and consumption of vegetable oil, oily fish, non-oily fish, coffee, tea, fruits, vegetable, unprocessed meat, processed meat, sugar or foods/drinks containing sugar, whole grain, refined grain, and family history of diabetes.

^a^ Metabolites identified by both baseline analysis and Mendelian randomization analysis.

**eTable 7. Metabolites positively associated with former smoking at baseline**

| **Metabolites** | **Baseline analysis ^a^** | | | |  | **Repeat assessment ^a^** | | | |
| --- | --- | --- | --- | --- | --- | --- | --- | --- | --- |
|  | **Sample size** | **β** | **SE** | ***p*** |  | **Sample size** | **β** | **SE** | ***p*** |
| **Inflammation** |  |  |  |  |  |  |  |  |  |
| Glycoprotein Acetyls | 93722 | 0.031 | 0.007 | <0.001 |  | 3797 | 0.029 | 0.034 | 0.396 |
| **Fatty acids** |  |  |  |  |  |  |  |  |  |
| SFA/FA | 93659 | 0.031 | 0.007 | <0.001 |  | 3796 | 0.004 | 0.032 | 0.902 |
| MUFA | 93659 | 0.024 | 0.007 | <0.001 |  | 3796 | 0.019 | 0.033 | 0.570 |
| MUFA/FA | 93659 | 0.036 | 0.006 | <0.001 |  | 3796 | 0.022 | 0.030 | 0.469 |
| **Concentrations of lipoproteins** |  |  |  |  |  |  |  |  |  |
| HDL_P | 93722 | 0.021 | 0.006 | 0.001 |  | 3797 | -0.006 | 0.031 | 0.846 |
| S_HDL_P | 93722 | 0.031 | 0.007 | <0.001 |  | 3797 | 0.040 | 0.034 | 0.240 |
| M_HDL_P | 93722 | 0.025 | 0.006 | <0.001 |  | 3797 | -0.026 | 0.031 | 0.404 |
| **Total Lipids** |  |  |  |  |  |  |  |  |  |
| S_HDL_L | 93722 | 0.040 | 0.007 | <0.001 |  | 3797 | 0.031 | 0.033 | 0.362 |
| M_HDL_L | 93722 | 0.026 | 0.006 | <0.001 |  | 3797 | -0.024 | 0.031 | 0.445 |
| **Phospholipids** |  |  |  |  |  |  |  |  |  |
| S_HDL_PL | 93722 | 0.042 | 0.007 | <0.001 |  | 3797 | 0.023 | 0.033 | 0.488 |
| M_HDL_PL | 93722 | 0.034 | 0.006 | <0.001 |  | 3797 | -0.021 | 0.032 | 0.510 |
| L_HDL_PL_pct | 93722 | 0.041 | 0.006 | <0.001 |  | 3797 | -0.004 | 0.026 | 0.887 |
| **Triglycerides** |  |  |  |  |  |  |  |  |  |
| XS_VLDL_TG_pct | 93722 | 0.054 | 0.006 | <0.001 |  | 3797 | 0.005 | 0.029 | 0.858 |
| S_VLDL_TG_pct | 93722 | 0.046 | 0.006 | <0.001 |  | 3797 | -0.012 | 0.030 | 0.683 |
| M_VLDL_TG_pct | 93722 | 0.043 | 0.006 | <0.001 |  | 3797 | 0.004 | 0.028 | 0.888 |
| L_VLDL_TG_pct | 93719 | 0.034 | 0.007 | <0.001 |  | 3797 | -0.029 | 0.035 | 0.410 |
| XL_VLDL_TG_pct | 93090 | 0.040 | 0.007 | <0.001 |  | 3776 | 0.026 | 0.033 | 0.430 |
| LDL_TG | 93722 | 0.027 | 0.007 | <0.001 |  | 3797 | 0.017 | 0.035 | 0.619 |
| S_LDL_TG_pct | 93722 | 0.034 | 0.007 | <0.001 |  | 3797 | -0.016 | 0.032 | 0.611 |
| M_LDL_TG | 93722 | 0.028 | 0.007 | <0.001 |  | 3797 | 0.019 | 0.035 | 0.594 |
| M_LDL_TG_pct | 93722 | 0.047 | 0.007 | <0.001 |  | 3797 | -0.029 | 0.031 | 0.356 |
| L_LDL_TG | 93722 | 0.029 | 0.007 | <0.001 |  | 3797 | 0.012 | 0.036 | 0.733 |
| L_LDL_TG_pct | 93722 | 0.049 | 0.006 | <0.001 |  | 3797 | -0.012 | 0.030 | 0.688 |
| IDL_TG_pct | 93722 | 0.047 | 0.006 | <0.001 |  | 3797 | -0.005 | 0.029 | 0.857 |
| XL_HDL_TG_pct | 93715 | 0.020 | 0.006 | 0.001 |  | 3797 | 0.023 | 0.030 | 0.458 |
| **Cholesterol** |  |  |  |  |  |  |  |  |  |
| S_HDL_C | 93722 | 0.036 | 0.007 | <0.001 |  | 3797 | 0.041 | 0.034 | 0.231 |
| M_HDL_C | 93722 | 0.025 | 0.006 | <0.001 |  | 3797 | -0.023 | 0.031 | 0.464 |
| **Free Cholesterol** |  |  |  |  |  |  |  |  |  |
| S_HDL_FC | 93722 | 0.030 | 0.007 | <0.001 |  | 3797 | 0.016 | 0.033 | 0.633 |
| M_HDL_FC | 93722 | 0.022 | 0.006 | <0.001 |  | 3797 | -0.028 | 0.031 | 0.362 |
| **Esterified cholesterol** |  |  |  |  |  |  |  |  |  |
| S_HDL_CE | 93722 | 0.035 | 0.007 | <0.001 |  | 3797 | 0.047 | 0.034 | 0.167 |
| M_HDL_CE | 93722 | 0.025 | 0.006 | <0.001 |  | 3797 | -0.021 | 0.031 | 0.494 |
| **Energy metabolism: glycolysis & ketone bodies** |  |  |  |  |  |  |  |  |  |
| Acetone | 93719 | 0.031 | 0.007 | <0.001 |  | 3797 | -0.024 | 0.035 | 0.491 |

SE: standard error; MUFA: monounsaturated fatty acids; SFA: saturated fatty acid; FA: fatty acids; VLDL: very-low-density lipoprotein; LDL: low-density lipoprotein; HDL: high-density lipoprotein; IDL: intermediate density lipoprotein; XS_: very small; S_: small; M_: medium; L_: large; XL_: very large; XXL_: extremely large; _P: particle concentrations of lipoproteins; _PL: phospholipids in lipoproteins; TG: triglycerides; _C: total cholesterol; _CE: esterified cholesterol; _FC: free cholesterol; _pct: percentage of certain lipids to total lipids in lipoproteins.

^a^ Linear regression models were fitted for the analyses of baseline and repeat assessment data, with never smoking as the reference group, and with adjustment for age, sex, fasting hours, body mass index, waist-to-hip ratio, physical activity, and consumption of alcohol, coffee, tea, whole grains, refined grains, fruit, vegetables, unprocessed meat, processed meat, fish, milk, and sugar-sweetened beverages. Metabolites in red were positively associated with former smoking at the repeat assessment analysis.

**eTable 8. Metabolites inversely associated with former smoking at baseline**

| **Metabolites** | **Baseline analysis ^a^** | | | |  | **Repeat assessment ^a^** | | | |
| --- | --- | --- | --- | --- | --- | --- | --- | --- | --- |
|  | **Sample size** | **β** | **SE** | ***p*** |  | **Sample size** | **β** | **SE** | ***p*** |
| **Amino acids** | | | | | | | | | |
| Alanine | 93718 | -0.028 | 0.007 | <0.001 |  | 3797 | 0.003 | 0.036 | 0.932 |
| **Fatty acids** | | | | | | | | | |
| LA/FA | 93659 | -0.048 | 0.006 | <0.001 |  | 3796 | -0.022 | 0.030 | 0.453 |
| PUFA/FA | 93659 | -0.043 | 0.006 | <0.001 |  | 3796 | -0.015 | 0.030 | 0.615 |
| PUFA/MUFA | 93659 | -0.040 | 0.006 | <0.001 |  | 3796 | -0.018 | 0.030 | 0.537 |
| FAw6/FA | 93659 | -0.037 | 0.006 | <0.001 |  | 3796 | -0.017 | 0.031 | 0.589 |
| Degree of Unsaturation | 93659 | -0.038 | 0.006 | <0.001 |  | 3796 | -0.002 | 0.030 | 0.938 |
| **Concentrations of lipoproteins** | | | | | | | | | |
| XS_VLDL_P | 93722 | -0.025 | 0.007 | <0.001 |  | 3797 | 0.014 | 0.032 | 0.647 |
| LDL_P | 93722 | -0.026 | 0.007 | <0.001 |  | 3797 | 0.032 | 0.032 | 0.308 |
| S_LDL_P | 93722 | -0.027 | 0.007 | <0.001 |  | 3797 | 0.027 | 0.032 | 0.394 |
| M_LDL_P | 93722 | -0.029 | 0.007 | <0.001 |  | 3797 | 0.032 | 0.032 | 0.318 |
| L_LDL_P | 93722 | -0.025 | 0.007 | <0.001 |  | 3797 | 0.032 | 0.031 | 0.306 |
| IDL_P | 93722 | -0.033 | 0.006 | <0.001 |  | 3797 | 0.027 | 0.029 | 0.350 |
| XL_HDL_P | 93722 | -0.022 | 0.006 | <0.001 |  | 3797 | -0.055 | 0.030 | 0.067 |
| **Total Lipids** | | | | | | | | | |
| XS_VLDL_L | 93722 | -0.023 | 0.007 | 0.001 |  | 3797 | 0.012 | 0.032 | 0.713 |
| IDL_L | 93722 | -0.028 | 0.006 | <0.001 |  | 3797 | 0.014 | 0.028 | 0.607 |
| XL_HDL_L | 93722 | -0.023 | 0.006 | <0.001 |  | 3797 | -0.048 | 0.027 | 0.073 |
| **Phospholipids** | | | | | | | | | |
| S_VLDL_PL_pct | 93722 | -0.039 | 0.006 | <0.001 |  | 3797 | 0.004 | 0.030 | 0.886 |
| M_VLDL_PL_pct | 93722 | -0.040 | 0.006 | <0.001 |  | 3797 | 0.009 | 0.028 | 0.746 |
| XL_VLDL_PL_pct | 93090 | -0.038 | 0.007 | <0.001 |  | 3776 | 0.006 | 0.034 | 0.859 |
| L_LDL_PL_pct | 93722 | -0.029 | 0.007 | <0.001 |  | 3797 | 0.040 | 0.034 | 0.236 |
| L_LDL_PL | 93722 | -0.024 | 0.006 | <0.001 |  | 3797 | 0.033 | 0.030 | 0.282 |
| IDL_PL | 93722 | -0.031 | 0.006 | <0.001 |  | 3797 | 0.008 | 0.028 | 0.770 |
| XL_HDL_PL | 93722 | -0.021 | 0.006 | 0.001 |  | 3797 | -0.057 | 0.031 | 0.060 |
| **Cholesterol** | | | | | | | | | |
| VLDL_C | 93722 | -0.024 | 0.006 | <0.001 |  | 3797 | 0.030 | 0.032 | 0.348 |
| XS_VLDL_C | 93722 | -0.036 | 0.006 | <0.001 |  | 3797 | 0.012 | 0.029 | 0.683 |
| XS_VLDL_C_pct | 93722 | -0.048 | 0.006 | <0.001 |  | 3797 | 0.003 | 0.027 | 0.912 |
| S_VLDL_C_pct | 93722 | -0.048 | 0.006 | <0.001 |  | 3797 | 0.016 | 0.031 | 0.611 |
| S_VLDL_C | 93722 | -0.026 | 0.007 | <0.001 |  | 3797 | 0.038 | 0.033 | 0.251 |
| M_VLDL_C_pct | 93722 | -0.043 | 0.006 | <0.001 |  | 3797 | -0.007 | 0.028 | 0.792 |
| M_VLDL_C | 93722 | -0.035 | 0.006 | <0.001 |  | 3797 | 0.029 | 0.028 | 0.309 |
| L_VLDL_C_pct | 93719 | -0.043 | 0.007 | <0.001 |  | 3797 | 0.007 | 0.033 | 0.840 |
| XL_VLDL_C_pct | 93090 | -0.034 | 0.006 | <0.001 |  | 3776 | -0.015 | 0.031 | 0.621 |
| S_LDL_C_pct | 93722 | -0.040 | 0.006 | <0.001 |  | 3797 | 0.045 | 0.031 | 0.148 |
| M_LDL_C_pct | 93722 | -0.040 | 0.006 | <0.001 |  | 3797 | 0.023 | 0.029 | 0.420 |
| L_LDL_C_pct | 93722 | -0.027 | 0.006 | <0.001 |  | 3797 | -0.000 | 0.027 | 0.999 |
| IDL_C | 93722 | -0.031 | 0.006 | <0.001 |  | 3797 | 0.017 | 0.027 | 0.536 |
| IDL_C_pct | 93722 | -0.032 | 0.006 | <0.001 |  | 3797 | 0.025 | 0.028 | 0.373 |
| L_HDL_C_pct | 93722 | -0.026 | 0.006 | <0.001 |  | 3797 | -0.011 | 0.027 | 0.670 |
| XL_HDL_C | 93722 | -0.025 | 0.005 | <0.001 |  | 3797 | -0.041 | 0.026 | 0.117 |
| Total Cholesterol Minus HDL-C | 93722 | -0.025 | 0.006 | <0.001 |  | 3797 | 0.029 | 0.030 | 0.348 |
| Clinical LDL Cholesterol | 93722 | -0.026 | 0.007 | <0.001 |  | 3797 | 0.042 | 0.033 | 0.206 |
| Remnant Cholesterol (Non-HDL, Non-LDL -Cholesterol) | 93722 | -0.029 | 0.006 | <0.001 |  | 3797 | 0.025 | 0.030 | 0.402 |
| **Free Cholesterol** | | | | | | | | | |
| XS_VLDL_FC_pct | 93722 | -0.048 | 0.006 | <0.001 |  | 3797 | 0.040 | 0.030 | 0.181 |
| XS_VLDL_FC | 93722 | -0.029 | 0.006 | <0.001 |  | 3797 | 0.017 | 0.031 | 0.587 |
| S_VLDL_FC | 93722 | -0.026 | 0.007 | <0.001 |  | 3797 | 0.036 | 0.032 | 0.261 |
| S_VLDL_FC_pct | 93722 | -0.038 | 0.006 | <0.001 |  | 3797 | 0.004 | 0.030 | 0.898 |
| M_VLDL_FC | 93722 | -0.028 | 0.006 | <0.001 |  | 3797 | 0.034 | 0.031 | 0.269 |
| M_VLDL_FC_pct | 93722 | -0.046 | 0.006 | <0.001 |  | 3797 | 0.005 | 0.028 | 0.865 |
| L_VLDL_FC_pct | 93719 | -0.024 | 0.007 | 0.001 |  | 3797 | 0.035 | 0.034 | 0.306 |
| XL_VLDL_FC_pct | 93090 | -0.039 | 0.007 | <0.001 |  | 3776 | -0.024 | 0.033 | 0.475 |
| LDL_FC | 93722 | -0.027 | 0.007 | <0.001 |  | 3797 | 0.036 | 0.033 | 0.274 |
| S_LDL_FC_pct | 93722 | -0.026 | 0.006 | <0.001 |  | 3797 | 0.023 | 0.031 | 0.465 |
| S_LDL_FC | 93722 | -0.025 | 0.007 | <0.001 |  | 3797 | 0.049 | 0.034 | 0.149 |
| M_LDL_FC_pct | 93722 | -0.026 | 0.006 | <0.001 |  | 3797 | -0.006 | 0.030 | 0.837 |
| L_LDL_FC | 93722 | -0.029 | 0.007 | <0.001 |  | 3797 | 0.030 | 0.032 | 0.351 |
| L_LDL_FC_pct | 93722 | -0.042 | 0.006 | <0.001 |  | 3797 | 0.010 | 0.028 | 0.724 |
| IDL_FC_pct | 93722 | -0.048 | 0.006 | <0.001 |  | 3797 | 0.028 | 0.031 | 0.373 |
| IDL_FC | 93722 | -0.036 | 0.006 | <0.001 |  | 3797 | 0.019 | 0.028 | 0.488 |
| S_HDL_FC_pct | 93722 | -0.023 | 0.007 | 0.001 |  | 3797 | -0.035 | 0.031 | 0.264 |
| XL_HDL_FC | 93722 | -0.031 | 0.006 | <0.001 |  | 3797 | -0.044 | 0.028 | 0.112 |
| Total Free Cholesterol | 93722 | -0.022 | 0.006 | <0.001 |  | 3797 | 0.017 | 0.029 | 0.556 |
| **Esterified cholesterol** | | | | | | | | | |
| VLDL_CE | 93722 | -0.030 | 0.006 | <0.001 |  | 3797 | 0.028 | 0.031 | 0.365 |
| XS_VLDL_CE | 93722 | -0.038 | 0.006 | <0.001 |  | 3797 | 0.010 | 0.028 | 0.728 |
| XS_VLDL_CE_pct | 93722 | -0.043 | 0.005 | <0.001 |  | 3797 | 0.000 | 0.025 | 0.986 |
| S_VLDL_CE | 93722 | -0.025 | 0.007 | <0.001 |  | 3797 | 0.039 | 0.033 | 0.248 |
| S_VLDL_CE_pct | 93722 | -0.051 | 0.007 | <0.001 |  | 3797 | 0.023 | 0.032 | 0.472 |
| M_VLDL_CE_pct | 93722 | -0.042 | 0.006 | <0.001 |  | 3797 | -0.012 | 0.028 | 0.681 |
| M_VLDL_CE | 93722 | -0.045 | 0.007 | <0.001 |  | 3797 | 0.036 | 0.032 | 0.271 |
| L_VLDL_CE_pct | 93719 | -0.046 | 0.006 | <0.001 |  | 3797 | 0.001 | 0.030 | 0.973 |
| XL_VLDL_CE_pct | 93090 | -0.032 | 0.006 | <0.001 |  | 3776 | -0.012 | 0.031 | 0.701 |
| IDL_CE | 93722 | -0.028 | 0.006 | <0.001 |  | 3797 | 0.016 | 0.027 | 0.558 |
| L_HDL_CE_pct | 93722 | -0.025 | 0.006 | <0.001 |  | 3797 | -0.006 | 0.028 | 0.823 |
| XL_HDL_CE | 93722 | -0.022 | 0.005 | <0.001 |  | 3797 | -0.026 | 0.027 | 0.344 |
| **Apolipoproteins** | | | | | | | | | |
| ApoB to ApoA1 | 93722 | -0.034 | 0.006 | <0.001 |  | 3797 | 0.046 | 0.032 | 0.159 |
| Apolipoprotein B | 93722 | -0.027 | 0.006 | <0.001 |  | 3797 | 0.032 | 0.031 | 0.304 |
| **Energy metabolism:glycolysis & ketone bodies** | | | | | | | | | |
| Glucose | 93552 | -0.022 | 0.006 | <0.001 |  | 3790 | -0.072 | 0.028 | 0.010 |
| Citrate | 93711 | -0.071 | 0.007 | <0.001 |  | 3796 | -0.145 | 0.034 | <0.001 |

SE: standard error; PUFA: polyunsaturated fatty acids; FAw6: omega−6 fatty acids; FA: fatty acids; LA: linoleic acid; VLDL: very-low-density lipoprotein; LDL: low-density lipoprotein; HDL: high-density lipoprotein; IDL: intermediate density lipoprotein; XS_: very small; S_: small; M_: medium; L_: large; XL_: very large; XXL_: extremely large; _P: particle concentrations of lipoproteins; _PL: phospholipids in lipoproteins; TG: triglycerides; _C: total cholesterol; _CE: esterified cholesterol; _FC: free cholesterol; _pct: percentage of certain lipids to total lipids in lipoproteins.

^a^ Linear regression models were fitted for the analyses of baseline and repeat assessment data, with never smoking as the reference group, with adjustment for age, sex, fasting hours, body mass index, waist-to-hip ratio, physical activity, and consumption of alcohol, coffee, tea, whole grains, refined grains, fruit, vegetables, unprocessed meat, processed meat, fish, milk, and sugar-sweetened beverages. Metabolites in red were inversely associated with former smoking at the repeat assessment analysis.

**eTable 9. Metabolites and corresponding coefficients estimated by elastic net regression in the full cohort and internal-external cross-validation**

| **Metabolites** | **beta (full cohort)** | **Coefficients in each cycle of internal-external cross-validation ^a^** | | | | | | | |
| --- | --- | --- | --- | --- | --- | --- | --- | --- | --- |
|  |  | **beta (cycle 1)** | **beta (cycle 2)** | **beta (cycle 3)** | **beta (cycle 4)** | **beta (cycle 5)** | **beta (cycle 6)** | **beta (cycle 7)** | **beta (cycle 8)** |
| **Amino acids** |  |  |  |  |  |  |  |  |  |
| Histidine | -0.0112574 | -0.0100947 | -0.0164151 | -0.0050032 | -0.0097478 | 0.0012021 | -0.0048532 | -0.0021701 | 0.0016364 |
| **Inflammation** |  |  |  |  |  |  |  |  |  |
| Glycoprotein Acetyls | 0.4627117 | 0.5169141 | 0.4809902 | 0.4724527 | 0.5157970 | 0.5278648 | 0.5056690 | 0.5058823 | 0.5228119 |
| **Fatty acids** |  |  |  |  |  |  |  |  |  |
| FAw6/FA | 0.2588597 | 0.2114714 | 0.2282466 | 0.1980674 | 0.2419968 | 0.2578637 | 0.2499026 | 0.1460151 | 0.2783222 |
| FAw3/FA | -0.4239405 | -0.3790313 | -0.3879003 | -0.4325653 | -0.3787511 | -0.3980110 | -0.4064264 | -0.4027468 | -0.3936854 |
| Degree of unsaturation | -0.6284664 | -1.0736030 | -0.8258637 | -0.8092635 | -1.0710070 | -0.9879171 | -0.9555390 | -1.0456953 | -1.1437280 |
| MUFA/FA | - | - | -0.0519812 | -0.1141912 | -0.1952667 | -0.1351615 | -0.0001029 | -0.0301246 | -0.1055065 |
| PUFA/FA | - | 0.0723538 | - | 0.0185530 | 0.0443711 | 0.0002334 | 0.0740682 | 0.4135375 | 0.3538274 |
| PUFA/MUFA | 0.0247087 | 0.5695259 | 0.2169963 | 0.1537423 | 0.3029011 | 0.2933186 | 0.3548556 | 0.2801565 | 0.2326738 |
| SFA | -0.0120990 | -0.0000454 | -0.0005727 | 0.0702784 | -0.0000016 | -0.0450218 | -0.0037787 | 0.0812191 | -0.0000501 |
| SFA/FA | -0.3428209 | -0.4993369 | -0.4537842 | -0.4552090 | -0.6009595 | -0.5309676 | -0.4432345 | -0.4397553 | -0.4991359 |
| DHA | - | - | 0.0000307 | 0.0758398 | 0.0151972 | 0.0002285 | - | 0.0072393 | - |
| Total Fatty Acids | - | -0.0114733 | -0.0288151 | 0.0013814 | - | - | - | 0.0113112 | -0.0000395 |
| DHA/FA | 0.0528453 | 0.0000735 | 0.0221905 | - | 0.0038250 | 0.0202568 | 0.0388479 | - | 0.0116987 |
| MUFA | - | - | - | - | -0.1002003 | -0.1377172 | - | -0.0017998 | -0.0946682 |
| LA/FA | -0.8312700 | -1.1116150 | -0.9377854 | -0.9356231 | -1.1096540 | -1.0948823 | -1.0352667 | -1.0873212 | -1.2264610 |
| **Concentrations of lipoproteins** | | | | | | | | | |
| S_VLDL_P | 0.2105693 | -0.0058994 | 0.0385248 | 0.2047930 | -0.1314191 | -0.0044597 | - | -0.2138166 | -0.1521263 |
| L_VLDL_P | 0.0004129 | 0.6514727 | 0.3112738 | 0.2617532 | 0.4941271 | 0.3647219 | 0.2635105 | 0.6696447 | 0.4117239 |
| XL_VLDL_P | - | 0.0799475 | - | - | 0.2627077 | - | - | 0.2602035 | 0.3505651 |
| XXL_VLDL_P | - | -0.0011373 | - | - | 0.0406292 | 0.0567605 | - | 0.0386203 | -0.1337872 |
| L_HDL_P | 0.0509082 | 0.5519137 | 0.4144095 | 0.1796098 | 0.4333041 | 0.5354185 | 0.0780319 | 0.5448552 | 1.0055130 |
| XL_HDL_P | 0.0005745 | -0.0369344 | 0.1359554 | 0.0103330 | -0.0913089 | - | - | -0.0927511 | 0.0552565 |
| **Diameters of lipoproteins** |  |  |  |  |  |  |  |  |  |
| VLDL_D | - | 0.0088110 | - | 0.0115272 | 0.1049887 | 0.1008185 | - | 0.2394458 | 0.0034543 |
| HDL_D | -0.2016228 | -0.6213340 | -0.4613477 | -0.4220758 | -0.7246630 | -0.6111286 | -0.4765631 | -0.7360913 | -0.8913694 |
| **Total Lipids** |  |  |  |  |  |  |  |  |  |
| VLDL_L | -0.1563732 | -1.0200180 | -0.3680797 | -0.4631289 | -0.9113357 | -1.6659121 | -0.3434959 | -0.9286087 | -1.1127850 |
| S_VLDL_L | 0.0000562 | 1.0412460 | 0.5155419 | 0.3866508 | 1.3158630 | 1.7204613 | 0.3411306 | 1.0371046 | 1.0805280 |
| L_VLDL_L | - | -0.0000712 | - | - | -0.0190012 | -0.0001718 | - | -0.0549451 | -0.0046606 |
| XL_VLDL_L | - | - | - | -0.0297200 | 0.0472988 | - | - | 0.1140396 | - |
| XXL_VLDL_L | 0.0000788 | 0.2643755 | 0.1377935 | 0.1755203 | 0.3186581 | 0.3177290 | 0.2216963 | 0.3207401 | 0.4384295 |
| L_HDL_L | - | 0.2813030 | - | 0.3206026 | 0.6381521 | 0.1573113 | 0.3275717 | 0.4660943 | 0.1897837 |
| XL_HDL_L | - | -0.0652803 | - | -0.0304862 | -0.1737177 | -0.0529375 | - | - | -0.0962804 |
| **Phospholipids** |  |  |  |  |  |  |  |  |  |
| VLDL_PL | - | -0.0214919 | -0.0204659 | -0.3146178 | -0.5044263 | -0.3331494 | -0.2161408 | -0.4747591 | -0.3258831 |
| XS_VLDL_PL_pct | 0.0004867 | 0.1182722 | 0.0215056 | 0.0285596 | 0.1292087 | 0.1431822 | 0.0419377 | 0.0755479 | 0.1450366 |
| S_VLDL_PL_pct | - | 0.0044116 | - | - | 0.0278351 | - | 0.0064471 | 0.0216475 | 0.0193982 |
| M_VLDL_PL_pct | -0.0001976 | -0.2814348 | -0.1517590 | -0.1110364 | -0.2711523 | -0.2322840 | -0.1494366 | -0.2361456 | -0.2865284 |
| L_VLDL_PL_pct | 0.3748746 | 0.6018543 | 0.5164744 | 0.5845627 | 0.5805032 | 0.6504451 | 0.5373235 | 0.6182241 | 0.6867547 |
| L_VLDL_PL | -0.0728226 | -0.2754539 | -0.1434966 | -0.1449960 | -0.3207500 | -0.2648269 | -0.1598923 | -0.2753608 | -0.3258522 |
| XL_VLDL_PL | - | 0.0640620 | 0.0043699 | -0.0067468 | 0.0806437 | 0.0830402 | - | 0.1798671 | 0.1148931 |
| XXL_VLDL_PL | -0.0733526 | -0.1679811 | -0.1051347 | -0.1282498 | -0.1895554 | -0.2645858 | -0.0890751 | -0.2400377 | -0.1278257 |
| XXL_VLDL_PL_pct | 0.0321623 | 0.0234624 | 0.0246802 | 0.0357508 | 0.0569489 | 0.0625075 | 0.0255834 | 0.0509278 | 0.0362398 |
| S_HDL_PL_pct | 0.4853080 | 0.5250498 | 0.5060123 | 0.5113290 | 0.3551322 | 0.3802297 | 0.6181529 | 0.3245076 | 0.5663384 |
| M_HDL_PL_pct | -0.2864102 | -0.1057663 | -0.1935700 | -0.1861834 | -0.1830213 | -0.0130297 | -0.2049827 | -0.1332319 | -0.1423814 |
| L_HDL_PL_pct | -0.1014668 | - | -0.0261486 | -0.1159780 | 0.0122535 | -0.0262671 | -0.1285575 | 0.0149775 | 0.0492015 |
| L_HDL_PL | - | -0.2225821 | -0.1975346 | -0.0954204 | -0.6893108 | -0.2086282 | -0.0455250 | -0.2434892 | -0.3862935 |
| XL_HDL_PL | 0.1571344 | 0.5348271 | 0.2894094 | 0.4226147 | 0.7651685 | 0.5337323 | 0.3356783 | 0.5640976 | 0.5011903 |
| **Triglycerides** |  |  |  |  |  |  |  |  |  |
| VLDL_TG | - | 0.3740013 | - | - | 0.3856122 | 1.1058405 | - | 0.4582679 | 0.6007654 |
| XS_VLDL_TG | -0.0000835 | -0.6688486 | -0.4814518 | -0.4097414 | -0.6497594 | -0.7431005 | -0.5263241 | -0.3454299 | -0.7752836 |
| XS_VLDL_TG_pct | - | 0.6026963 | 0.2750202 | 0.3145701 | 0.4667192 | 0.5465289 | 0.3620892 | 0.2731806 | 0.4495748 |
| S_VLDL_TG_pct | - | - | - | - | -0.0000066 | - | - | - | - |
| S_VLDL_TG | 0.4962705 | 0.5454886 | 0.8757836 | 0.4723705 | 0.6276498 | 0.4000330 | 0.8501068 | 0.8459259 | 0.6985571 |
| M_VLDL_TG | 0.2380335 | -0.0304698 | - | 0.3199297 | -0.1287746 | -0.3107993 | 0.2716044 | 0.1412519 | 0.2546748 |
| M_VLDL_TG_pct | - | 0.0350026 | - | - | 0.0200410 | - | 0.0094294 | - | - |
| L_VLDL_TG | 0.1864305 | 0.3779696 | 0.2008731 | 0.5670264 | 0.4677134 | 0.5901871 | 0.3717457 | 0.3845149 | 0.4746459 |
| XL_VLDL_TG_pct | 0.2580892 | 0.3448143 | 0.1755375 | 0.2256952 | 0.3143036 | 0.2379399 | 0.2111171 | 0.1904390 | 0.2580934 |
| XL_VLDL_TG | - | -0.2975150 | -0.0005364 | - | -0.3785230 | -0.3214450 | - | -0.1496672 | -0.3232773 |
| XXL_VLDL_TG | 0.0094521 | -0.0531664 | -0.0082975 | -0.0190672 | -0.0466445 | -0.0666039 | -0.0046172 | -0.0317896 | -0.0326533 |
| LDL_TG | 0.4464650 | 1.5756600 | 0.5827077 | 0.8276931 | 1.2775730 | 1.7156304 | 1.2281099 | 0.9885065 | 1.2141300 |
| S_LDL_TG | - | 0.0817869 | - | -0.0759696 | 0.3125438 | 0.0103962 | - | 0.3399112 | 0.3020789 |
| S_LDL_TG_pct | -1.2352390 | -1.3562120 | -1.4058530 | -1.1720123 | -1.4665280 | -1.4742618 | -1.1674684 | -1.4980033 | -1.4293920 |
| M_LDL_TG | 0.4302295 | -0.0363901 | 0.3259993 | 0.3560945 | -0.0078129 | -0.0085818 | 0.1193146 | -0.0971719 | 0.1388269 |
| M_LDL_TG_pct | 0.2273903 | 0.6960566 | 0.3513988 | 0.3597508 | 0.6803085 | 0.6090503 | 0.3865799 | 0.7231119 | 0.3815478 |
| L_LDL_TG | -0.2641925 | -0.2811715 | -0.2179289 | -0.2424191 | -0.3807287 | -0.2191328 | -0.2926374 | -0.2390602 | -0.3600371 |
| L_LDL_TG_pct | 0.5961881 | 0.1819770 | 0.8398361 | 0.5714920 | 0.4537666 | 0.1767155 | 0.3368118 | 0.6974770 | 0.5434885 |
| IDL_TG | - | -0.0791471 | -0.0033573 | - | -0.0010978 | - | - | -0.0605032 | - |
| IDL_TG_pct | - | 0.0010310 | - | - | 0.1137003 | 0.0040632 | - | 0.0469912 | 0.2394824 |
| HDL_TG | - | -0.5556856 | -0.0685617 | -0.0888301 | -0.7207812 | -0.4296892 | -0.1938974 | -0.8171706 | -0.3805447 |
| S_HDL_TG_pct | - | -0.0003598 | 0.0041913 | 0.1412988 | -0.0108336 | -0.0008605 | 0.0961708 | -0.0336702 | -0.0000048 |
| S_HDL_TG | - | 0.2964347 | 0.0008693 | 0.0330078 | 0.3628126 | 0.2254241 | - | 0.2922609 | 0.2358527 |
| M_HDL_TG_pct | 0.4112930 | 2.3565900 | 1.1327950 | 0.8595354 | 2.1234030 | 2.8632463 | 1.4599034 | 1.9768916 | 2.2823300 |
| M_HDL_TG | -0.5687585 | -2.0335440 | -1.1951710 | -0.9261262 | -1.6237700 | -2.4750734 | -1.3431101 | -1.4235476 | -1.9956030 |
| L_HDL_TG_pct | 0.0732162 | 0.0297589 | 0.0571432 | 0.0871114 | -0.1423704 | 0.0608114 | - | -0.0166648 | 0.0177381 |
| XL_HDL_TG_pct | 0.0000105 | 0.6114109 | 0.2976419 | 0.2465647 | 0.7701537 | 0.6159345 | 0.3776624 | 0.8889713 | 0.6763343 |
| TG | -0.0874367 | - | -0.0013434 | -0.5059627 | -0.1760024 | - | -0.0014431 | -0.4721532 | - |
| TG/PG | -1.1652930 | -2.6849900 | -1.7510270 | -1.4654928 | -2.4137580 | -2.6303178 | -2.0253613 | -2.3269754 | -2.8702130 |
| **Cholesterol** |  |  |  |  |  |  |  |  |  |
| XS_VLDL_C_pct | - | 0.1727994 | - | 0.0664729 | 0.2123748 | 0.1602058 | - | 0.1911835 | 0.3100436 |
| S_VLDL_C_pct | - | -0.0008733 | - | -0.1014971 | -0.0049489 | - | - | -0.0252407 | -0.0012900 |
| M_VLDL_C_pct | - | -0.0035719 | - | -0.0055997 | -0.0718505 | -0.1034639 | - | -0.1159197 | -0.0001345 |
| L_VLDL_C_pct | - | -0.0975442 | -0.0434884 | -0.0722329 | 0.0116362 | 0.0076306 | - | 0.0210430 | - |
| L_VLDL_C | 0.2713024 | 0.9925362 | 0.6127102 | 0.3061908 | 0.7560212 | 0.9254320 | 0.7059635 | 0.6842247 | 0.6544781 |
| XL_VLDL_C_pct | - | 0.2653528 | 0.0000526 | 0.0671996 | 0.3455383 | 0.2416921 | - | 0.1478134 | 0.4615998 |
| XL_VLDL_C | -0.0255308 | -0.3636946 | -0.3747281 | -0.3218085 | -0.2870158 | -0.1994160 | -0.4491061 | -0.7749522 | -0.0343092 |
| XXL_VLDL_C | - | -0.4870668 | - | -0.1917809 | -0.4805730 | -0.6483293 | -0.3001605 | -0.1599987 | -0.3426301 |
| M_LDL_C_pct | - | 0.0513293 | -0.0316310 | 0.0141159 | 0.0223016 | 0.0080944 | - | 0.0806248 | - |
| L_LDL_C_pct | 0.1103367 | - | 0.2135500 | 0.0833252 | 0.0549522 | - | 0.0855331 | 0.1418930 | 0.0492540 |
| IDL_C_pct | - | - | - | 0.0360800 | 0.1148940 | - | - | 0.0785451 | - |
| IDL_C | - | - | - | - | - | - | - | - | - |
| HDL_C | -0.2133664 | - | -0.1959872 | -0.3594377 | -0.0527033 | 0.0005377 | -0.0722247 | -0.0814724 | - |
| S_HDL_C_pct | - | 0.0040412 | - | - | 0.1270785 | - | - | 0.0769079 | - |
| M_HDL_C_pct | 0.2388064 | 0.5178304 | 0.3762604 | 0.3100960 | 0.2235452 | 0.7689518 | 0.5228433 | 0.2962465 | 0.0213918 |
| L_HDL_C | - | -0.1053163 | - | -0.0027008 | -0.1323507 | -0.2372391 | - | -0.3852681 | -0.2336917 |
| L_HDL_C_pct | 0.1028964 | -0.2607283 | - | - | -0.2148743 | -0.1492981 | - | -0.1572960 | -0.3731916 |
| XL_HDL_C | - | 0.6417727 | 0.2046788 | 0.0953815 | 1.0704910 | 0.7368169 | 0.2118617 | 0.7710034 | 0.9027832 |
| **Cholesterol** |  |  |  |  |  |  |  |  |  |
| XS_VLDL_FC_pct | - | -0.1472421 | - | -0.0203679 | -0.0717534 | -0.0949720 | -0.0183917 | -0.1236296 | -0.1181804 |
| S_VLDL_FC_pct | 0.2056781 | 0.6959579 | 0.4781339 | 0.4433002 | 0.7910181 | 0.7781136 | 0.5700259 | 0.9813473 | 0.6034283 |
| M_VLDL_FC_pct | 0.4899974 | 0.9192459 | 0.7948765 | 0.7795999 | 0.9028717 | 0.9357834 | 0.7716918 | 0.9974130 | 1.0392730 |
| L_VLDL_FC_pct | 0.0389973 | -0.0793340 | - | -0.0430501 | -0.1266714 | -0.1199167 | -0.0091562 | -0.1516383 | -0.1660158 |
| L_VLDL_FC | - | 0.0106863 | - | 0.2343586 | 0.2457559 | -0.0173611 | - | 0.1601759 | 0.0379587 |
| XL_VLDL_FC | - | - | - | - | -0.1306241 | - | - | -0.2103520 | -0.1843842 |
| XL_VLDL_FC_pct | 0.2571651 | 0.2271930 | 0.2397504 | 0.3016152 | 0.2355154 | 0.2743259 | 0.2610459 | 0.2340191 | 0.2230474 |
| XXL_VLDL_FC | 0.0452421 | 0.4924679 | 0.1262799 | 0.2317530 | 0.3665172 | 0.4867872 | 0.1943174 | 0.2951739 | 0.3053630 |
| S_LDL_FC_pct | -0.1248165 | -0.3544411 | -0.2470946 | -0.2609476 | -0.3799702 | -0.3492747 | -0.2922401 | -0.3356945 | -0.3811481 |
| M_LDL_FC_pct | - | 0.1741347 | - | 0.0413596 | 0.1420963 | 0.0911947 | 0.0269893 | 0.1070092 | 0.1089998 |
| L_LDL_FC_pct | 0.1620061 | 0.0979796 | 0.1277643 | 0.0202732 | 0.1227304 | 0.1043828 | 0.1130566 | 0.0632465 | 0.0932940 |
| L_LDL_FC | 0.0927806 | -0.0094750 | 0.0706185 | 0.1887168 | 0.0342875 | -0.0862134 | 0.0449557 | 0.0457452 | -0.0616225 |
| IDL_FC | - | -0.2467972 | - | - | -0.3725590 | -0.3775300 | -0.0163247 | -0.2695461 | -0.2670095 |
| IDL_FC_pct | -0.1373885 | 0.0079721 | -0.1207521 | -0.1053203 | 0.0115057 | 0.0256313 | -0.0682361 | 0.0152913 | 0.0033409 |
| HDL_FC | - | 0.1055166 | - | -0.1063726 | 0.0824432 | 0.0273808 | - | 0.0380051 | 0.0032814 |
| S_HDL_FC_pct | -0.5296550 | -1.0265160 | -0.8294908 | -0.8258816 | -1.1301140 | -1.2084682 | -0.8258941 | -1.0711710 | -0.9290140 |
| M_HDL_FC_pct | 0.2031060 | 0.5680949 | 0.4227110 | 0.4594013 | 0.7087379 | 0.6528269 | 0.3867781 | 0.6200008 | 0.6093647 |
| L_HDL_FC | - | -0.0757243 | -0.0078066 | -0.0024901 | 0.0342382 | - | - | -0.0893239 | 0.0312345 |
| XL_HDL_FC | 0.0473983 | -0.2035988 | - | -0.1199166 | -0.4422700 | -0.2958361 | - | -0.2739145 | -0.3158589 |
| XL_HDL_FC_pct | 0.0126378 | 0.0974747 | 0.0285129 | 0.0781614 | 0.1067384 | 0.1483948 | - | 0.1244422 | 0.1589931 |
| **Esterified cholesterol** |  |  |  |  |  |  |  |  |  |
| XS_VLDL_CE_pct | 0.0898190 | 0.3577847 | 0.2333643 | 0.2122106 | 0.3526728 | 0.3864173 | 0.1615678 | 0.1906064 | 0.3224961 |
| S_VLDL_CE_pct | -0.7902670 | -0.8933971 | -0.7676883 | -0.8134381 | -0.8167800 | -0.9111540 | -0.8525125 | -0.7878415 | -0.8965036 |
| M_VLDL_CE_pct | -0.0786875 | -0.3508948 | -0.3230146 | -0.1441955 | -0.4182585 | -0.3570528 | -0.1870451 | -0.4457130 | -0.3301344 |
| L_VLDL_CE_pct | 0.0650737 | 0.0806889 | - | 0.1602051 | 0.0473546 | -0.0042513 | 0.0681308 | 0.0904650 | 0.0173476 |
| XL_VLDL_CE_pct | -0.2044423 | -0.3293772 | -0.1721432 | -0.2272924 | -0.4215220 | -0.4452456 | -0.1636934 | -0.2166201 | -0.5565309 |
| XXL_VLDL_CE | -0.1958407 | -0.0952279 | -0.2651970 | -0.1797056 | -0.0697428 | 0.0019821 | -0.1084778 | -0.2166534 | -0.1452012 |
| M_LDL_CE_pct | -0.0400766 | - | -0.0019611 | - | - | - | -0.0307155 | -0.0242198 | -0.0248064 |
| L_LDL_CE_pct | - | -0.0186270 | - | - | 0.0000899 | 0.0112641 | - | - | 0.0000485 |
| IDL_CE_pct | 0.0165388 | 0.1158848 | 0.0789564 | 0.0708274 | 0.0465006 | 0.1585860 | 0.0868236 | 0.1189931 | 0.1022320 |
| IDL_CE | - | 0.0000752 | - | - | 0.1856828 | 0.0146764 | - | 0.1058578 | 0.1307119 |
| HDL_CE | -0.5606141 | -0.6984216 | -0.5972472 | -0.5944546 | -0.6371877 | -0.2442114 | -0.8187152 | -0.6865887 | -0.5947486 |
| S_HDL_CE_pct | - | -0.2158667 | -0.0007439 | -0.0521769 | -0.4957237 | -0.4126535 | - | -0.5558973 | -0.1257776 |
| M_HDL_CE_pct | - | 0.0865390 | 0.0002934 | 0.2007307 | 0.2591675 | 0.0132950 | 0.0001016 | 0.4005506 | 0.4546138 |
| L_HDL_CE_pct | 0.0090001 | 0.3395003 | 0.0553830 | 0.0363222 | 0.1708625 | 0.1520480 | - | 0.2905842 | 0.4694658 |
| L_HDL_CE | 0.0955285 | 0.0430437 | 0.2073490 | 0.0039064 | 0.1699485 | 0.1621139 | 0.1181739 | 0.1503281 | - |
| XL_HDL_CE | - | 0.0890611 | -0.0418468 | 0.2781286 | 0.0143677 | 0.1007374 | 0.0004798 | 0.2263569 | 0.0810099 |
| XL_HDL_CE_pct | -0.1345214 | -0.2119481 | -0.1492581 | -0.1705346 | -0.2713254 | -0.2621523 | -0.1388145 | -0.2928623 | -0.2385387 |
| **Energy metabolism:glycolysis & ketone bodies** | | | | | | | | | |
| Acetate | -0.0622517 | -0.0625981 | -0.0740600 | -0.0656953 | -0.0721019 | -0.0652362 | -0.0609438 | -0.0653888 | -0.0839664 |
| Citrate | -0.3142111 | -0.3289949 | -0.3084347 | -0.3140166 | -0.3199384 | -0.3215013 | -0.3213657 | -0.3259407 | -0.3179614 |
| **Fluid balance** |  |  |  |  |  |  |  |  |  |
| Albumin | 0.0111212 | 0.0215886 | 0.0140249 | 0.0050843 | 0.0158466 | 0.0069672 | 0.0137590 | 0.0171169 | 0.0175359 |

PUFA: polyunsaturated fatty acids; MUFA: monounsaturated fatty acids; FA: total fatty acids; SFA: saturated fatty acids; DHA: docosahexaenoic acid; LA: linoleic acid; FAw6: omega−6 fatty acids; FAw3: omega−3 fatty acids; VLDL: very-low-density lipoprotein; LDL: low-density lipoprotein; HDL: high-density lipoprotein; IDL: intermediate density lipoprotein; XS_: very small; S_: small; M_: medium; L_: large; XL_: very large; XXL_: extremely large; _P: particle concentrations of lipoproteins; _PL: phospholipids in lipoproteins; TG: triglycerides; _C: total cholesterol; _CE: esterified cholesterol; _FC: free cholesterol; _pct: percentage of certain lipids to total lipids in lipoproteins.

^a^ Assessment centers left out from the testing set and treated as validation set: Bury and Reading (cycle 1); Newcastle and Stoke (cycle 2); Leeds and Middlesborough (cycle 3); Bristol and Edinburgh (cycle 4); Nottingham and Croydon (cycle 5); Sheffield and Hounslow (cycle 6); Liverpool and Birmingham (cycle 7); Manchester, Oxford, Stockport, Glasgow, and Barts (cycle 8).

**eTable 10. HR (95% CI) of type 2 diabetes in relation to smoking and smoking-related metabolic signature in UK Biobank**

| **Sex** | **Smoking status** | |  | **Metabolic signature** | | |
| --- | --- | --- | --- | --- | --- | --- |
|  | **Never** | **Current** |  | **Low** | **High** | **Per SD increase** |
| Overall |  |  |  |  |  |  |
| Cases | 1,415 | 454 |  | 715 | 1,154 | 1,869 |
| Person years | 653,081 | 116,290 |  | 387,442 | 381,929 | 769,371 |
| Incidence rate (per 1000 person-years) | 2.2 | 3.9 |  | 1.8 | 3.0 | 2.4 |
| HR (95% CI) ^a^ | 1.00 | 1.73 (1.54-1.94) |  | 1.00 | 1.61 (1.46-1.77) | 1.31 (1.26-1.37) |
| HR (95% CI) ^b^ | 1.00 | 1.46 (1.29-1.65) |  | - | - | - |
| Smoking-diabetes association mediated by metabolic signature: 38.3%  38.3% | | | | | | |
| Men |  |  |  |  |  |  |
| Cases | 658 | 454 |  | 350 | 577 | 1,869 |
| Person years | 257,493 | 116,290 |  | 162,123 | 154,704 | 769,371 |
| Incidence rate (per 1000 person-years) | 2.6 | 3.9 |  | 2.2 | 3.7 | 2.4 |
| HR (95% CI) ^a^ | 1.00 | 1.62 (1.38-1.90) |  | 1.00 | 1.68 (1.47-1.93) | 1.32 (1.24-1.39) |
| HR (95% CI) ^b^ | 1.00 | 1.36 (1.15-1.60) |  | - | - | - |
| Smoking-diabetes association mediated by metabolic signature: 43.6% | | | | | | |
| Women |  |  |  |  |  |  |
| Cases | 757 | 454 |  | 365 | 577 | 1,869 |
| Person years | 395,588 | 116,290 |  | 225,319 | 227,225 | 769,371 |
| Incidence rate (per 1000 person-years) | 1.9 | 3.9 |  | 1.6 | 2.5 | 2.4 |
| HR (95% CI) ^a^ | 1.00 | 1.89 (1.58-2.27) |  | 1.00 | 1.52 (1.33-1.74) | 1.32 (1.24-1.42) |
| HR (95% CI) ^b^ | 1.00 | 1.61 (1.33-1.94) |  | - | - | - |
| Smoking-diabetes association mediated by metabolic signature: 30.4% | | | | | | |

SD: standard deviation; HR: hazard ratio; CI: confidence interval.

^a^ Cox models were fitted with attained age as the time scale, and with adjustment for assessment center, education, ethnicity, Townsend deprivation index, body mass index, alcohol intake, physical activity, consumption of vegetable oil, oily fish, non-oily fish, coffee, tea, fruits, vegetable, unprocessed meat, processed meat, sugar or foods/drinks containing sugar, whole grain, refined grain, and family history of diabetes. The model in women were also adjusted for menopausal status.

^b^ Cox models were further adjusted for metabolic signature on the basis of model a.

**eTable 11. HR (95% CI) of type 2 diabetes in relation to smoking and smoking-related metabolic signature in TwinGene**

|  | **Smoking status** | |  | **Metabolic signature** | | |
| --- | --- | --- | --- | --- | --- | --- |
|  | **Never** | **Current** |  | **Low ^c^** | **High ^c^** | **Per SD increase** |
| Cases | 149 | 95 |  | 103 | 141 | 244 |
| Person years | 18,051 | 11,327 |  | 14,766 | 14,611 | 29,378 |
| Incidence rate (per 1000 person-years) | 8.3 | 8.4 |  | 7.0 | 9.7 | 8.3 |
| HR (95% CI) ^a^ | 1.00 | 1.29 (0.97-1.72) |  | 1.00 | 1.34 (1.04-1.74) | 1.26 (1.11-1.42) |
| HR (95% CI) ^b^ | 1.00 | 1.12 (0.83-1.52) |  | - | - | - |
| Smoking-diabetes association mediated by metabolic signature: 55.1%  38.3% | | | | | | |

SD: standard deviation; HR: hazard ratio; CI: confidence interval.

^a^ Cox models were fitted with attained age as the time scale, and with adjustment for education, ethnicity, body mass index, alcohol intake, and physical activity. Cluster-robust standard errors were applied to account for the correlation within Twin pairs.

^b^ Cox models were further adjusted for metabolic signature based on model a.

^c^ Above (high) or below (low) the median level.

**eTable 12. Summary of previous observational studies on smoking and metabolites based on metabolomics data**

| **Year, author** | **Country** | **Population** | **Sample size** | **Men,%** | **Age, years** | **Baseline year** | **Exposure** | **No. of metabolites** | **Metabolite measurement** | **Model, covariates and correction for multiple testing** | **Associations** |
| --- | --- | --- | --- | --- | --- | --- | --- | --- | --- | --- | --- |
| 2022, Maitre L^[8]^ | UK, France, Spain, Greece, Lithuania, and Norway | The Human Early Life Exposome (HELIX) study (children); population-based cohort study | 1301 |  | 6-11 |  | Childhood exposure to second-hand smoking and cotinine biomarker of tobacco exposure) | 177 metabolites (amino acids, biogenic amines, acylcarnitines, glycerophospholipids, sphingolipids and sum of hexoses | LC-MS/MS | linear: chort, age, sex, BMI, ancestry, maternal education; significance level: divided the nominal p value by the effective number of tests | Childhood exposure to cotinine was associated with lower levels of sphingomyelin (OH) C16:1, sphingomyelin (OH) C22:2, sphingomyelin (OH) C14:1, sphingomyelin (OH) C24:1, PC aa C38:0, PC aa C42:0, PC ae C38:0, PC ae C38:3, PC ae C36:1, PC ae C42:0, and PC ae C40:6. |
| 2022, R. Zhang^[9]^ | US | the Bogalusa Heart Study (residents from Bogalusa, Louisiana) | 1252 (654 in MR) | 41.3 | mean 48.2 | 2013-2016 | Current smoking, former smoking | 956 known + 510 unknown | HPLC | Linear regression (metabolites as the outcome): age, sex, race, education, drinking and physical activity | A total of 116 metabolites of known identities and 52 unknown metabolites showed significant associations with current cigarette smoking. The 116 known metabolites included 56 lipids, 22 xenobiotics, 14 amino acids, 10 cofactors and vitamins, 6 carbohydrates, 6 nucleotides, and 2 peptides. Although the levels were much lower in former smokers than current smokers, eight of the xenobiotics were also associated with former smoking. |
| 2021, Wang Q^[10]^ | US | 6 smokers and 6 healthy controls enrolled at the General Clinical Research Center of the University of Rochester Medical Center | 12 | 50 | 21-65 |  | Cigarette Smokers |  | UPLC-MS |  | cigarette smoking with increased concentrations of cotinine, cotinine N-oxide, (S)-nicotine, and (R)-6-hydroxynicotine |
| 2020, Vives-Usano M^[11]^ | UK, France, Spain, Greece, Lithuania, and Norway | The Human Early Life Exposome (HELIX) study (children) | 1203 | 54.5 | mean 8.1 |  | Childhood exposure to second-hand smoking | 177 | LC-MS/MS | linear: age, sex, batch, fasting time and hours of blood collection, childhood BMI, maternal smoking during pregnancy; divided the nominal p value by the effective number of tests | Childhood exposure to second-hand smoking was associated with lower levels of sphingomyelin (OH) C16:1, carnitine C9, and cotinine: PC ae C38.0 |
| 2017, Goettel M^[12]^ |  | Healthy male individuals who were followed over the course of three months after quitting smoking | 39 | 100 |  |  | smoking cessation | 44 (fatty acids) | untargeted: GC-TOF-MS | Wilcoxon signed rank test: Bonferroni correction | SFAs showed an increase while MUFAs declined over the time of smoking cessation |
| 2016, Lacruz ME^[13]^ | Germany | The CARLA (Cardiovascular disease, Living and Ageing in Halle) study: a population-based cohort study in an elderly population of the city of Halle/Saale in eastern Germany. | 1030 | 52 | 45-83 | 2002-2006 | pack-years of smoking | 163 (28 acylcarnitines, 14 amino acids, 9 lysophosphocholines, 72 phosphocholines, 10 sphingomyelins and sum of hexoses) | Targeted: AbsoluteIDQ kit p150 (Biocrates) | Multilevel or simple linear regression modeling adjusted for relevant covariates was used for the evaluation of cross-sectional or longitudinal associations, respectively; FDR | **Cross-sectional**: Pack-years of tobacco were positively associated with 7 acylC predominantly in men, and negatively associated with a lyso PC. Seven diacyl PCs were positively associated with tobacco in the total population and in men only; 6 acyl-alkyl PCs were negatively associated with tobacco in women. Smoking showed positive associations with acylcarnitines and arginine. **Longitudinal**: Pack-years of tobacco were associated with an increase in levels of PC aa C32:1. |
| 2016, Gu F^[14]^ | Italy, USA, China and Finland | Participants from the EAGLE study (population controls), the PLCO Cancer Screening Trial (incident colorectal cancer cases and controls), the ATBC study (smokers randomily assined to different vitamin groups), and Shanghai Physical Activity Study | 892 | 80% in EAGLE, 56.2% in PLCO, 100% in ATBC, 100% in SPAS |  | 1993-2008 | current smoking status and cigarettes per day |  | Untargeted: HPLC-MS | Linear regression with adjustment for sex and coffeee consumption (age, education, BMI, alcohol intake, and other dietary factors were also adjusted for in sensitivity analysis); Bonferroni correction | 24 metabolites were associated with current smoking status and eight with cig/day. In addition to three well-established nicotine metabolites, we found an additional 12 xenobiotic metabolites involved in benzoatic or xanthine metabolism, three amino acids (o-cresol sulphate, serotonin, indolepropionate), two lipids, four vitamins or cofactors, and one carbohydrate (oxalate) |
| 2014, Müller DC^[15]^ |  | Healthy male smokers and nonsmokers from a nutrition controlled (24 h) in-house clinical study | 50 | 100 |  |  | current smoking vs never smoking | fatty acids | GC–TOF–MS, GC–FID, and HILIC–ESI–MS/MS | Mann–Whitney U test: no covariate | Smokers had higher levels of phosphatidylcholine (PC) and phosphatidylethanolamine (PE) species containing MUFA than nonsmokers |
| 2014, Cross AJ^[16]^ | US | Participants from a nested case-control study within the screening arm of the Prostate, Lung, Colorectal and Ovarian cancer screening trial | 509 | 56 | 55-74 | 1993-2001 | Current smoking vs never smoking | 446 | HPLC-MS | linear regression: age, gender, race, study center and BMI; Bonferroni correction | Self-reported current smoking was associated with serum cotinine, O-cresol sulfate and hydroxycotinine |
| 2013, Xu T^[17]^ | Germany | KORA (the population-based Cooperative Health Research in the Region of Augsburg human cohort) | 1241 | 47.9 | 55-74 | 1999-2001 | Current smoking vs never smoking, smoking cessation | 140 [one hexose, 21 amino acids, eight biogenic amines, 21 acylcarnitines, 13 sphingo myelins (SMs), eight lysoPCs, 33 diacyl-PCs (PC aa Cx:y) and 35 acyl-alkyl-PCs (PC ae Cx:y)] | targeted, the AbsoluteIDQ P180 and P150 kits | linear regresssion (metabolites as outcomes): age, BMI and alcohol; FDR p<0.05 | **Cross-sctional:**  In men, comapred to never smoking or former smoking, current smoking was associated with higher concentrations of four unsaturated diacyl-PCs and five amino acids (arginine, aspartate, glutamate, ornithine and serine), but lower levels of three saturated diacyl-PCs, one lysoPC and four acyl-alkyl-PCs,and kynurenine. In women, current smoking was associated with higher levels of carnitine and PC aa C32:1, and a lower level of hydroxysphingomyeline (SM (OH)) C22:2. Among the 21 smoking-related metabolites (18 in men and six in women), 19 were found to be reversible (that is, significant difference between former smokers and current smokers but without significant difference between former smokers and never smokers. No irreversible metabolite was observed  **Longitudinal**:  10 of the 13 reversible metabolites in men were confirmed to be reversible in male quitters. |
| 2013, Hsu PC^[18]^ | US | Not detailed information | 655 |  |  |  | Current smoking vs nonsmoking |  | UPLC-QTOF-MS | ANOVA, | Current smokers had higher levels of Cotinine, 3-Hydroxycotinine, Cotinine N-oxide, Pseudooxynicotine, Nicotine, Cysteine-S-sulfate, 7a,12a-dihydroxy-3-oxo-4-cholenoic acid, Trans-3-hydroxycotinine glucuronide, and lower levels of Aminoparathion, 1,11-Undecanedicarboxylic acid, 3-Hydroxycoumarin, Alpha-CEHC than nonsmokers |
| 2010, Beauchamp A^[19]^ |  |  | 612 | 41.2 | 40-69 | 1990-1994 |  |  | NMR | linear: alcohol and dietary intake, physical activity, and weight | mean total LDL particle concentration was greater for female smokers than nonsmokers. Both medium- and small-LDL particle concentrations contributed to this difference. Total HDL and large-HDL particle concentrations were lower for female smokers than nonsmokers. For men, there were few smoking-related differences in lipoprotein measures. |
| 2008, Wang-Sattler R^[20]^ | Germany | KORA (the population-based Cooperative Health Research in the Region of Augsburg human cohort) | 283 |  |  |  | current smoking vs never smoking, former smoking | 198 |  |  | The levels of 23 lipid metabolites were up-regulated in smokers compared to those in former and non-smokers, except for three acyl-alkyl-phosphatidylcholines (e.g. plasmalogens). Consistently significant results were further found for the ratios of plasmalogens to diacyl-phosphatidylcolines, which are reduced in smokers |


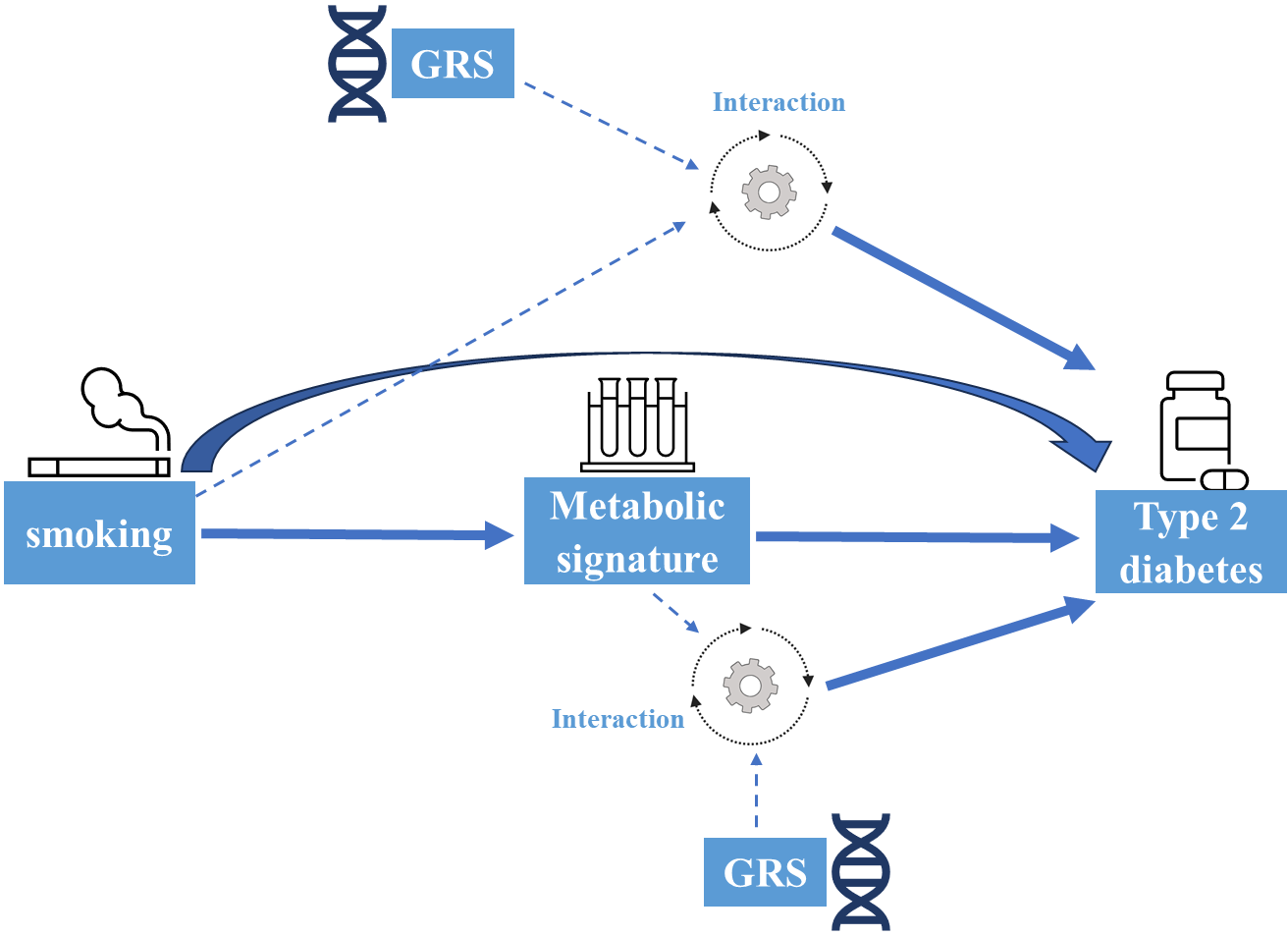


**eFigure 1. Conceptual figure for the role of smoking, metabolic signature on the risk of type 2 diabetes and the interaction with genetic susceptibility**

GRS genetic risk scores for insulin resistance or type 2 diabetes.

**eFigure 2. Variance in 131 smoking-related metabolites explained by smoking status and other factors**

BMI: body mass index; WHR: waist-to-hip ratio; PUFA: polyunsaturated fatty acids; MUFA: monounsaturated fatty acids; LA: linoleic acid; FA: total fatty acids; SFA: saturated fatty acids; FAw6: omega−6 fatty acids; FAw3: omega−3 fatty acids; VLDL: very-low-density lipoprotein; LDL: low-density lipoprotein; HDL: high-density lipoprotein; IDL: intermediate density lipoprotein; XS_: very small; S_: small; M_: medium; L_: large; XL_: very large; XXL_: extremely large; _P: particle concentrations of lipoproteins; _PL: phospholipids in lipoproteins; _TG: triglycerides; _C: total cholesterol; _CE: esterified cholesterol; _FC: free cholesterol; _pct: percentage of certain lipids to total lipids in lipoproteins.


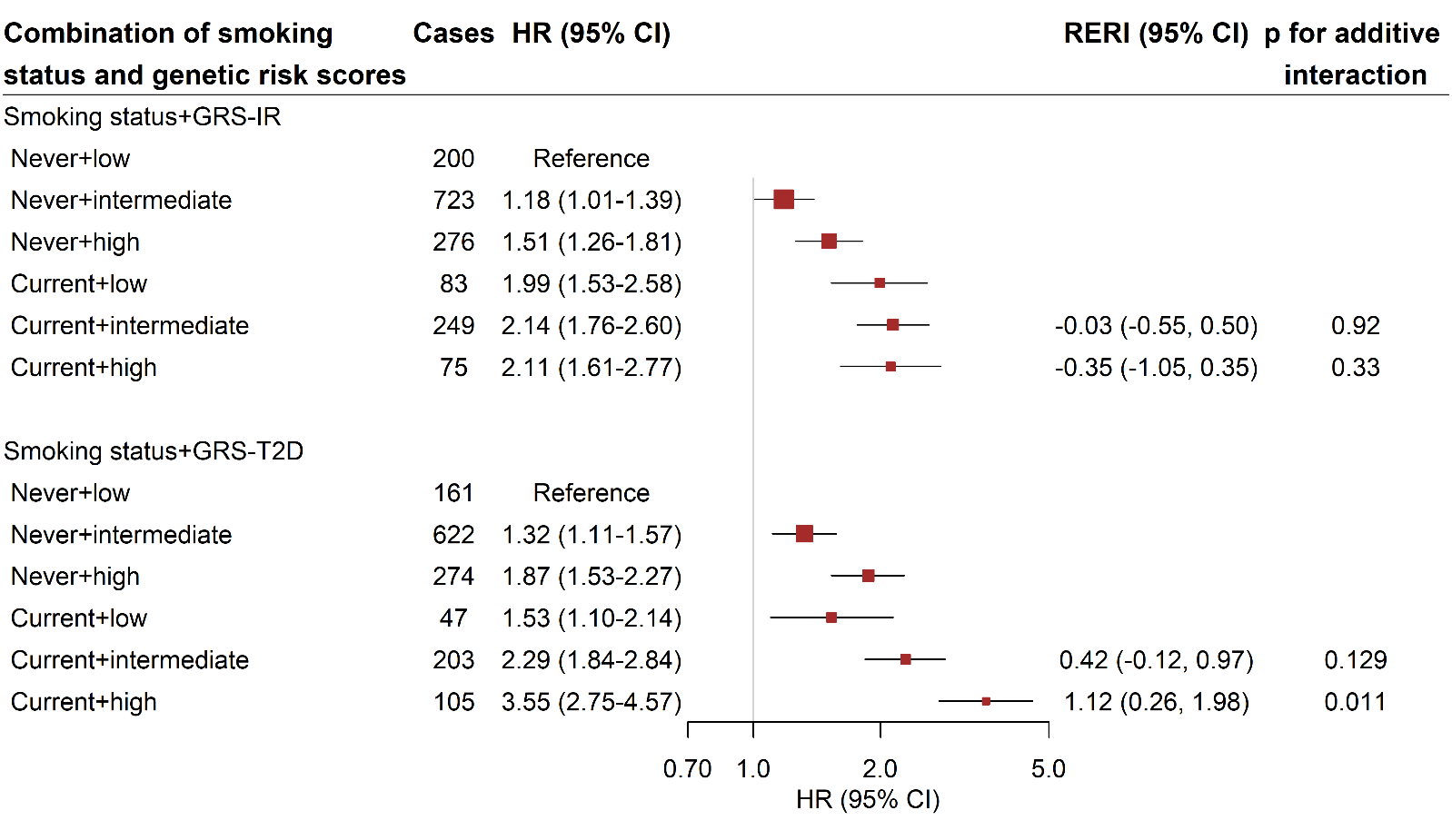


**eFigure 3. Joint analysis of type 2 diabetes in relation to different combinations of smoking status and genetic susceptibility in UK Biobank**

GRS-IR: genetic risk score for insulin resistance; GRS_T2D: genetic risk score for type 2 diabetes; HR: hazard ratio; CI: confidence interval. Cox models were fitted with attained age as the time scale, and with adjustment for age groups (through stratification), sex (through stratification), genetic batch, the first 10 genetic principal components, assessment center, education, Townsend deprivation index, body mass index , alcohol intake, physical activity, and consumption of vegetable oil, oily fish, non-oily fish, coffee, tea, fruits, vegetable, unprocessed meat, processed meat, sugar or foods/drinks containing sugar, whole grain, and refined grain.


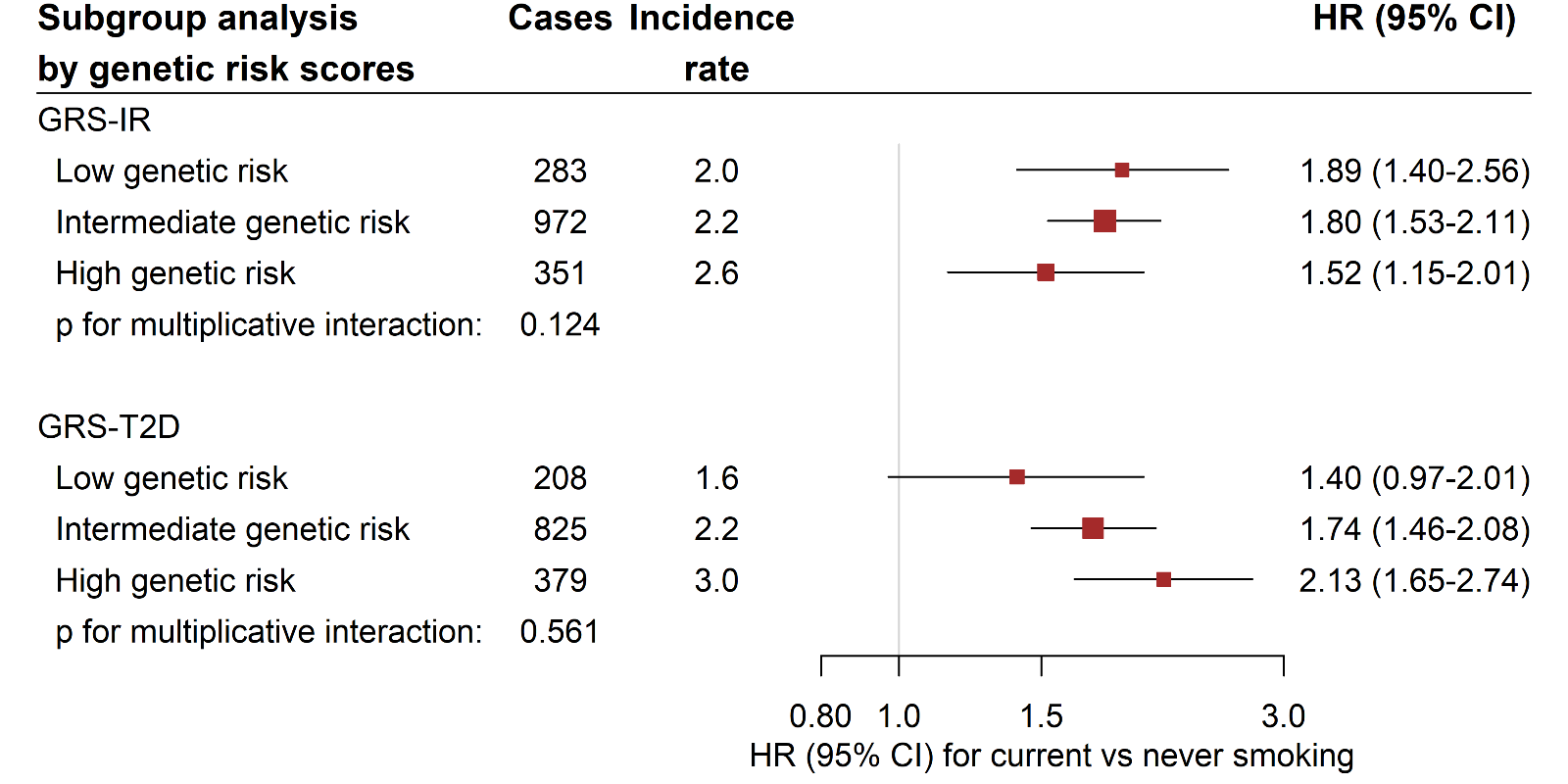


**eFigure 4. Smoking status and type 2 diabetes incidence by genetic susceptibility subgroups in UK Biobank**

GRS-IR: genetic risk score for insulin resistance; GRS_T2D: genetic risk score for type 2 diabetes; HR: hazard ratio; CI: confidence interval. Cox models were fitted with attained age as the time scale, and with adjustment for age groups (through stratification), sex (through stratification), genetic batch, the first 10 genetic principal components, assessment center, education, Townsend deprivation index, body mass index , alcohol intake, physical activity, and consumption of vegetable oil, oily fish, non-oily fish, coffee, tea, fruits, vegetable, unprocessed meat, processed meat, sugar or foods/drinks containing sugar, whole grain, and refined grain.


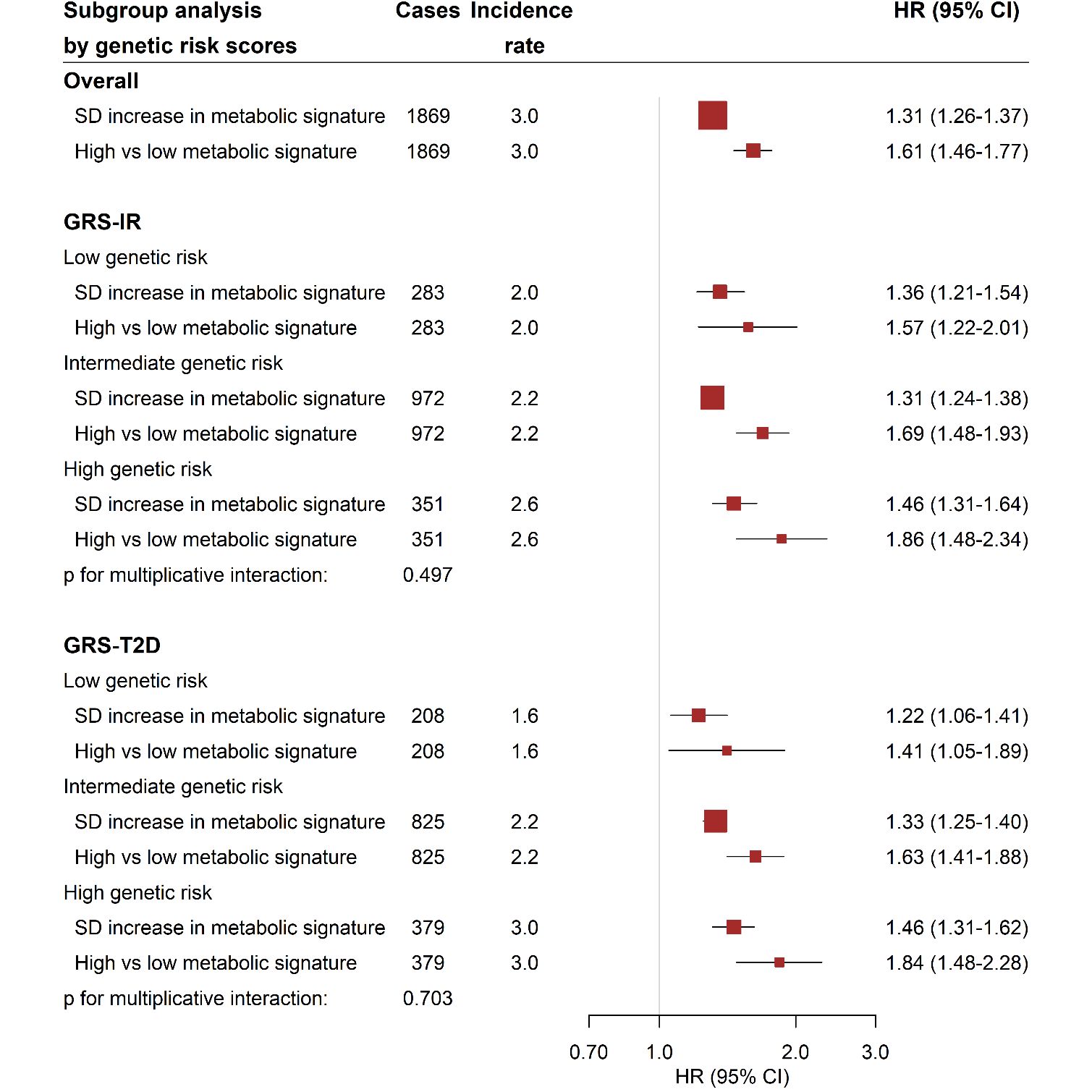


**eFigure 5. Smoking-related metabolic signature and type 2 diabetes incidence by genetic susceptibility subgroups in UK Biobank**

GRS-IR: genetic risk score for insulin resistance; GRS_T2D: genetic risk score for type 2 diabetes; SD: standard deviation; HR: hazard ratio; CI: confidence interval. Cox models were fitted with attained age as the time scale, and with adjustment for age groups (through stratification), sex (through stratification), genetic batch, the first 10 genetic principal components, assessment center, education, Townsend deprivation index, body mass index , alcohol intake, physical activity, and consumption of vegetable oil, oily fish, non-oily fish, coffee, tea, fruits, vegetable, unprocessed meat, processed meat, sugar or foods/drinks containing sugar, whole grain, and refined grain.


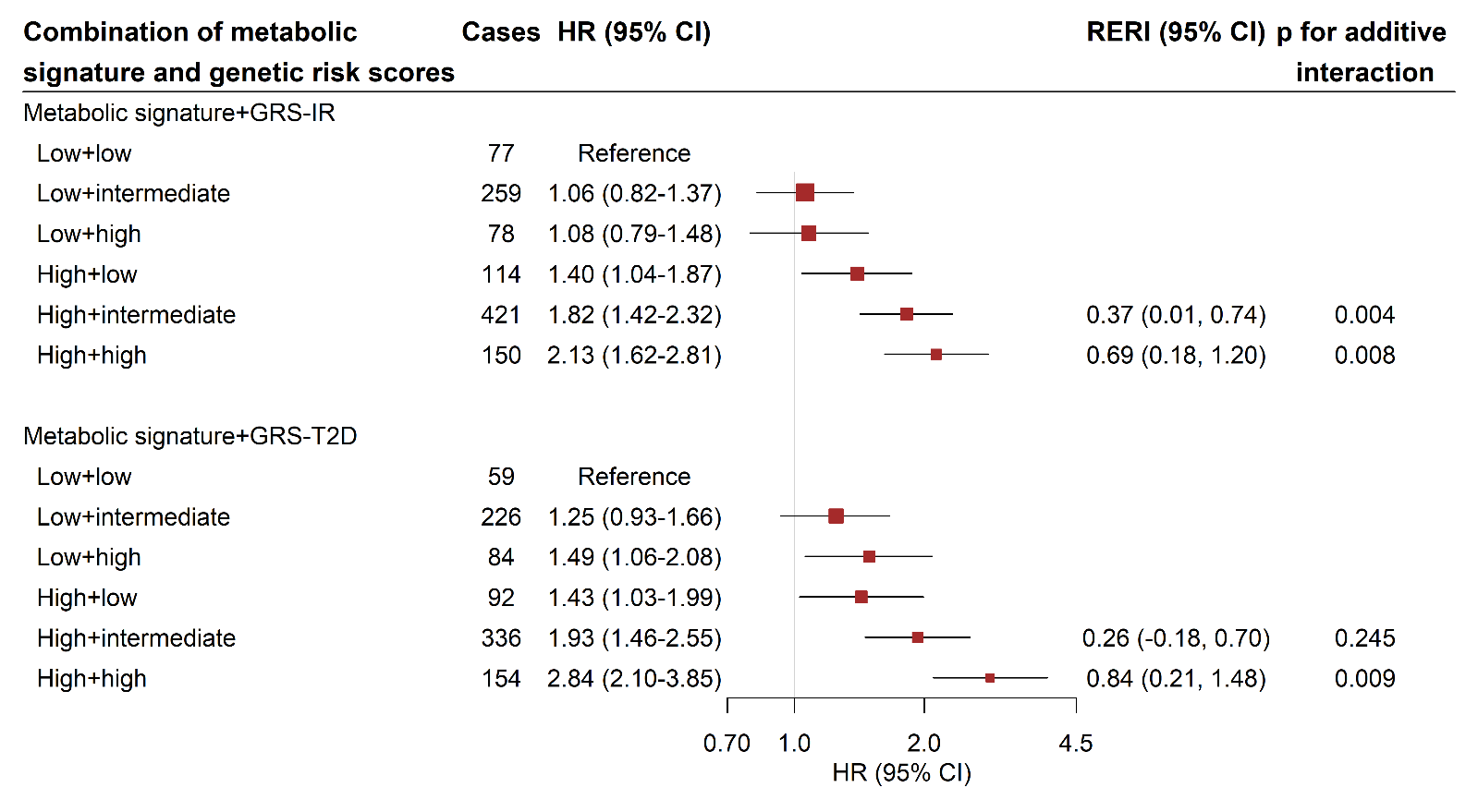


**eFigure 6. Joint analysis of type 2 diabetes in relation to different combinations of metabolic signature and genetic susceptibility in unrelated participants in UK Biobank**

GRS: genetic risk score; GRS-IR: genetic risk score for insulin resistance; GRS_T2D: genetic risk score for type 2 diabetes; HR: hazard ratio; CI: confidence interval; RERI: relative excess risk due to interaction (additive); SD: standard deviation.

Cox models were fitted with attained age as the time scale, and with adjustment for age groups (through stratification), sex (through stratification), genetic batch, the first 10 genetic principal components, assessment center, education, Townsend deprivation index, body mass index, alcohol intake, physical activity, and consumption of vegetable oil, oily fish, non-oily fish, coffee, tea, fruits, vegetable, unprocessed meat, processed meat, sugar or foods/drinks containing sugar, whole grain, refined grain.


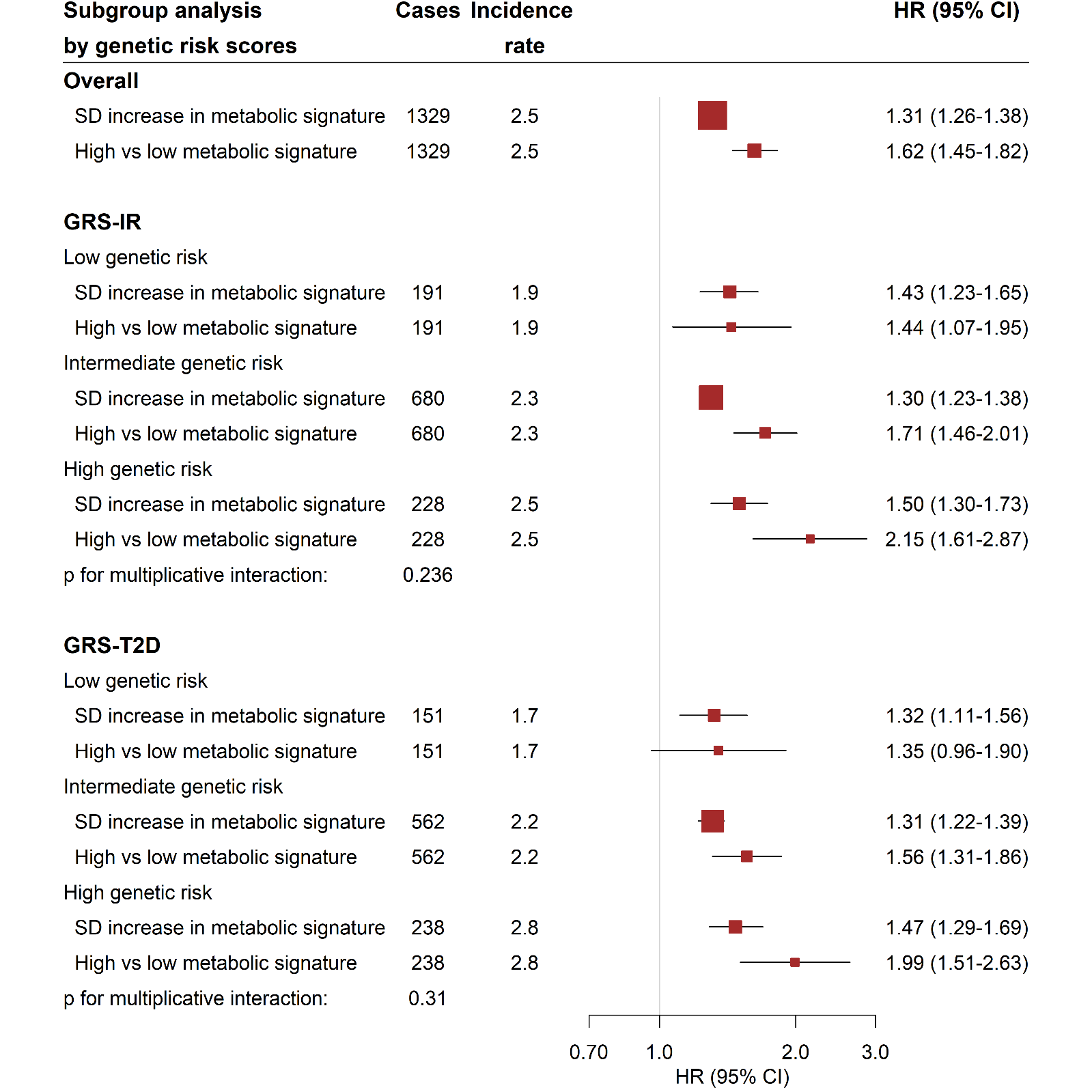


**eFigure 7. Smoking-related metabolic signature and type 2 diabetes incidence by genetic susceptibility subgroups in unrelated participants in UK Biobank**

GRS-IR: genetic risk score for insulin resistance; GRS_T2D: genetic risk score for type 2 diabetes; SD: standard deviation; HR: hazard ratio; CI: confidence interval. Cox models were fitted with attained age as the time scale, and with adjustment for age groups (through stratification), sex (through stratification), genetic batch, the first 10 genetic principal components, assessment center, education, Townsend deprivation index, body mass index , alcohol intake, physical activity, and consumption of vegetable oil, oily fish, non-oily fish, coffee, tea, fruits, vegetable, unprocessed meat, processed meat, sugar or foods/drinks containing sugar, whole grain, and refined grain.

### **References**

1. Xu K, Li B, McGinnis KA, et al. Genome-wide association study of smoking trajectory and meta-analysis of smoking status in 842,000 individuals. Nat Commun 2020;11(1):5302. (In eng). DOI: 10.1038/s41467-020-18489-3.

2. Burgess S, Butterworth A, Thompson SG. Mendelian randomization analysis with multiple genetic variants using summarized data. Genet Epidemiol 2013;37(7):658-65. (In eng). DOI: 10.1002/gepi.21758.

3. Verbanck M, Chen CY, Neale B, Do R. Detection of widespread horizontal pleiotropy in causal relationships inferred from Mendelian randomization between complex traits and diseases. Nat Genet 2018;50(5):693-698. (In eng). DOI: 10.1038/s41588-018-0099-7.

4. Ganna A, Fall T, Salihovic S, et al. Large-scale non-targeted metabolomic profiling in three human population-based studies. Metabolomics 2015;12(1):4. DOI: 10.1007/s11306-015-0893-5.

5. Dumanski JP, Rasi C, Lönn M, et al. Mutagenesis. Smoking is associated with mosaic loss of chromosome Y. Science 2015;347(6217):81-3. (In eng). DOI: 10.1126/science.1262092.

6. Riley RD, Ensor J, Snell KI, et al. External validation of clinical prediction models using big datasets from e-health records or IPD meta-analysis: opportunities and challenges. BMJ 2016;353:i3140. (In eng). DOI: 10.1136/bmj.i3140.

7. Yu D, Jordan KP, Snell KIE, et al. Development and validation of prediction models to estimate risk of primary total hip and knee replacements using data from the UK: two prospective open cohorts using the UK Clinical Practice Research Datalink. Ann Rheum Dis 2019;78(1):91-99. (In eng). DOI: 10.1136/annrheumdis-2018-213894.

8. Maitre L, Bustamante M, Hernández-Ferrer C, et al. Multi-omics signatures of the human early life exposome. Nat Commun 2022;13(1):7024. (In eng). DOI: 10.1038/s41467-022-34422-2.

9. Zhang R, Sun X, Huang Z, et al. Examination of serum metabolome altered by cigarette smoking identifies novel metabolites mediating smoking-BMI association. Obesity (Silver Spring) 2022;30(4):943-952. (In eng). DOI: 10.1002/oby.23386.

10. Wang Q, Ji X, Rahman I. Dysregulated Metabolites Serve as Novel Biomarkers for Metabolic Diseases Caused by E-Cigarette Vaping and Cigarette Smoking. Metabolites 2021;11(6) (In eng). DOI: 10.3390/metabo11060345.

11. Vives-Usano M, Hernandez-Ferrer C, Maitre L, et al. In utero and childhood exposure to tobacco smoke and multi-layer molecular signatures in children. BMC Med 2020;18(1):243. (In eng). DOI: 10.1186/s12916-020-01686-8.

12. Goettel M, Niessner R, Pluym N, Scherer G, Scherer M. A fully validated GC-TOF-MS method for the quantification of fatty acids revealed alterations in the metabolic profile of fatty acids after smoking cessation. J Chromatogr B Analyt Technol Biomed Life Sci 2017;1041-1042:141-150. (In eng). DOI: 10.1016/j.jchromb.2016.12.035.

13. Lacruz ME, Kluttig A, Tiller D, et al. Cardiovascular Risk Factors Associated With Blood Metabolite Concentrations and Their Alterations During a 4-Year Period in a Population-Based Cohort. Circ Cardiovasc Genet 2016;9(6):487-494. (In eng). DOI: 10.1161/circgenetics.116.001444.

14. Gu F, Derkach A, Freedman ND, et al. Cigarette smoking behaviour and blood metabolomics. Int J Epidemiol 2016;45(5):1421-1432. (In eng). DOI: 10.1093/ije/dyv330.

15. Müller DC, Degen C, Scherer G, Jahreis G, Niessner R, Scherer M. Metabolomics using GC-TOF-MS followed by subsequent GC-FID and HILIC-MS/MS analysis revealed significantly altered fatty acid and phospholipid species profiles in plasma of smokers. J Chromatogr B Analyt Technol Biomed Life Sci 2014;966:117-26. (In eng). DOI: 10.1016/j.jchromb.2014.02.044.

16. Cross AJ, Boca S, Freedman ND, et al. Metabolites of tobacco smoking and colorectal cancer risk. Carcinogenesis 2014;35(7):1516-22. (In eng). DOI: 10.1093/carcin/bgu071.

17. Xu T, Holzapfel C, Dong X, et al. Effects of smoking and smoking cessation on human serum metabolite profile: results from the KORA cohort study. BMC Med 2013;11:60. (In eng). DOI: 10.1186/1741-7015-11-60.

18. Hsu PC, Zhou B, Zhao Y, et al. Feasibility of identifying the tobacco-related global metabolome in blood by UPLC-QTOF-MS. J Proteome Res 2013;12(2):679-91. (In eng). DOI: 10.1021/pr3007705.

19. Beauchamp A, Tonkin A, Peeters A, et al. Associations among smoking status, lifestyle and lipoprotein subclasses. J Clin Lipidol 2010;4(6):522-30. (In eng). DOI: 10.1016/j.jacl.2010.09.003.

20. Wang-Sattler R, Yu Y, Mittelstrass K, et al. Metabolic profiling reveals distinct variations linked to nicotine consumption in humans--first results from the KORA study. PLoS One 2008;3(12):e3863. (In eng). DOI: 10.1371/journal.pone.0003863.
